# Unveiling asymmetric topological photonic states in anisotropic 2D perovskite microcavities

**DOI:** 10.1038/s41377-025-01852-8

**Published:** 2025-05-29

**Authors:** Emmanouil G. Mavrotsoupakis, Leonidas Mouchliadis, Junhui Cao, Minoas C. Chairetis, Marios E. Triantafyllou-Rundell, Eleni C. P. Macropulos, Giannis G. Paschos, Apostolos Pantousas, Huaying Liu, Alexey V. Kavokin, Hamid Ohadi, Constantinos C. Stoumpos, Pavlos G. Savvidis

**Affiliations:** 1 Institute of Electronic Structure and Laser, Foundation for Research and Technology-Hellas, Heraklion, 71110 Crete Greece; 2 Department of Materials Science and Engineering, University of Crete, Heraklion, Crete 70013 Greece; 3 Key Laboratory for Quantum Materials of Zhejiang Province, Department of Physics, Westlake University, Hangzhou, People’s Republic of China; 4 Westlake University, 600 Dunyu Rd, Xihu District, Hangzhou, Zhejiang 310030 People’s Republic of China; 5 Center for Quantum Science & Technologies, FORTH-QuTech, Crete 70013 Heraklion, Greece; 6 Abrikosov Center for Theoretical Physics, MIPT, Dolgoprudny, Moscow Region, 141701 Russia; 7 Shanghai Key Laboratory of Special Artificial Microstructure Materials and Technology, School of Physics Science and Engineering, Tongji University, Shanghai, 200092 China; 8 SUPA, School of Physics and Astronomy, University of St. Andrews, St. Andrews, KY16 9SS UK; 9 Photonics of Crystals Laboratory, Saint Petersburg State University, Ulyanovskaya d.1, St. Petersburg, 198504 Russia

**Keywords:** Polaritons, Microresonators

## Abstract

Photonic Rashba-Dresselhaus coupling in anisotropic microcavities offers a compelling platform for realizing unconventional topological states with non-zero Berry curvature. In this study, we explore a self-assembled two-dimensional hybrid structure composed of anisotropically oriented organic/inorganic halide perovskite layers confined within a microcavity. The strong optical anisotropies of these perovskite systems, driven by significant refractive index contrasts and robust excitonic resonances at room temperature, enable the emergence of synthetic magnetic fields that mediate photonic and polaritonic interactions. The interplay between polarization-dependent modes and spatial inversion symmetry breaking gives rise to strong photonic Rashba-Dresselhaus spin-orbit coupling, leading to distinct modifications in band topology and energy dispersions. These effects result in the formation of unconventional topological features, including non-zero Berry curvature and off-axis diabolical points, within the photonic and polaritonic bands at room temperature. Our findings reveal the critical role of optical and geometric anisotropies in engineering synthetic gauge fields for light, providing a versatile approach for designing photonic systems with novel topological properties. By leveraging the unique properties of halide perovskites and their ability to support both room-temperature excitons and large birefringence, this work advances the development of polaritonic platforms for applications in topological photonics and spinoptronics.

## Abstract (web summary)

Unconventional topological states emerge in anisotropic perovskite microcavities via strong photonic Rashba-Dresselhaus coupling, enabling synthetic gauge fields and non-zero Berry curvature for advanced topological photonics and spinoptronics.

## Graphical abstract

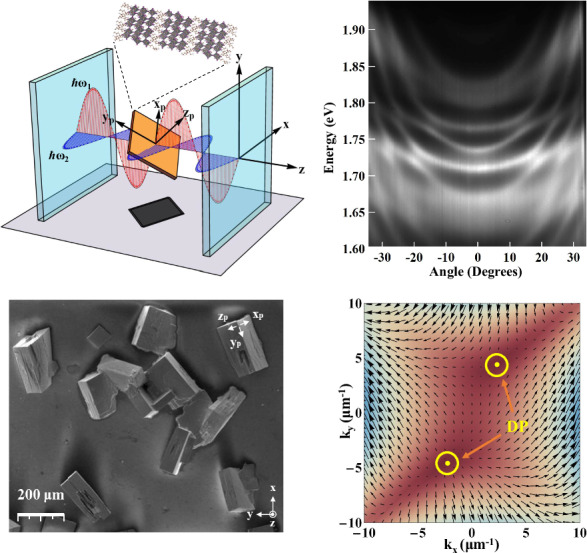

## Introduction

Artificial gauge fields have found applications across many disciplines, including solid-state physics, photonics, and quantum simulations^1^. These synthesized fields emulate the dynamics of charged particles in the presence of a real magnetic field. In condensed matter physics, this concept becomes prominent through spin-orbit coupling (SOC), where the coupling between spin and orbital degrees of freedom is described by a wavevector-dependent effective magnetic field. This is particularly significant in structures lacking inversion symmetry, where characteristic Rashba^2^ and Dresselhaus^3^ (RD) SOC occur. Non-Abelian gauge fields due to RD SOC play a central role in spintronics, valleytronics and topological insulators^4,5^ and are fundamental to phenomena such as the spin-Hall effect^6,7^ and the quantum spin-Hall effect^8–10^.

These effects are intricately linked to topological concepts and are related to time-reversal symmetry and topological invariants^11,12^. The Berry curvature, resembling a pseudomagnetic artificial field, is a key factor in the study of topological materials that plays a role in various phenomena, including the anomalous Hall effect and topological insulators. Topological effects in photonics enable light-based structures enduring perturbations and defects^13^, crucial for advancements in spinoptronics, signal processing and quantum optics. The challenge lies in controlling photons within scalable platforms operating at room temperature^14,15^. To meet this goal, recent attempts have focused on the design of new materials, geometries and artificial lattices^16–19^.

A promising approach towards topological photonics involves the exploration of exciton-polaritons in microcavities (MCs). An exciton-polariton is a quasiparticle resulting from the strong coupling of cavity photons with semiconductor quantum well excitons^20–22^. In dielectric microcavities, the intrinsic TE-TM splitting varies with the wavevector and acts as an effective magnetic field on the light pseudospin (polarization), analogous to the electronic RD SOC, a phenomenon known as the optical spin Hall effect^23,24^ (OSHE). To establish artificial gauge fields and topological states in polaritonic microcavities, the system Hamiltonian requires additional terms beyond TE-TM splitting, that couple momentum and pseudospin degrees of freedom, thus breaking the spatial or temporal inversion symmetry. Symmetry breaking can be achieved either by inserting the microcavity into an external magnetic field which acts on the excitonic component of the polariton^25,26^ and/or by designing microscale structures with reduced symmetry^27–29^. Using optically anisotropic materials such as liquid crystals or perylenes is another way for the realization of topological polaritons^30–33^.

Exciton-polaritons emerging from perovskite crystal microcavities hold immense potential for realizing polaritonic circuitry^34^, XY Hamiltonian^35,36^, topologically protected states^37^, and photonic artificial gauge fields^38–45^. In many of these works, non-trivial topological phenomena are achieved by mixing the birefringent liquid crystals with light emitting perovskite crystals, separating the functionalities of emission and structural anisotropy. In this study, we exploit the properties of the homologous ${\left({{\rm{CH}}}_{3}{\left({{\rm{CH}}}_{2}\right)}_{3}{\rm{N}}{{\rm{H}}}_{3}\right)}_{2}{\left({{\rm{CH}}}_{3}{\rm{N}}{{\rm{H}}}_{3}\right)}_{n-1}{{\rm{Pb}}}_{n}{{\rm{I}}}_{3n+1}$ halide perovskite series, crystallizing into two-dimensional (2D) periodic lattice structures formed by alternating organic and multiple (*n*) inorganic layers. In these crystalline materials, strong quantum and dielectric confinement provides robust exciton binding energy, typically exceeding several hundred ${\rm{meV}}$^46,47^, overcoming room temperature thermal fluctuations. They can be easily synthesized, and their chemical composition can be precisely controlled and periodically expanded to the macroscale^48,49^ making them highly promising for optoelectronic applications^50^ (see Supplementary Material (SM) S.1 for an overview of halide perovskites in microcavities). Furthermore, due to their orthorhombic structure, these crystals exhibit intrinsic optical anisotropy, functioning as uniaxial materials with very large linear birefringence.

Herein, we report on the first evidence of an exciton-polariton mode observed at room temperature for the $n=3$ perovskite compound, a material featuring both excellent optoelectronic properties and large anisotropy^51,52^. At the same time, by implementing a facile solution processing method, without the need of a separate birefringent material, the crystals self-assemble and orient freely in a fully anisotropic distributed Bragg reflector (DBR) microcavity, resulting in strong photonic RD SOC that leads to the emergence of non-zero Berry curvature and unconventional diabolical points. Such generation of non-trivial and tunable topological features in the strong coupling regime offers the means for designing and engineering topological insulators in a polaritonic platform^53^.

## Results

### Hybrid perovskite microcavities

**Fig. 1:**
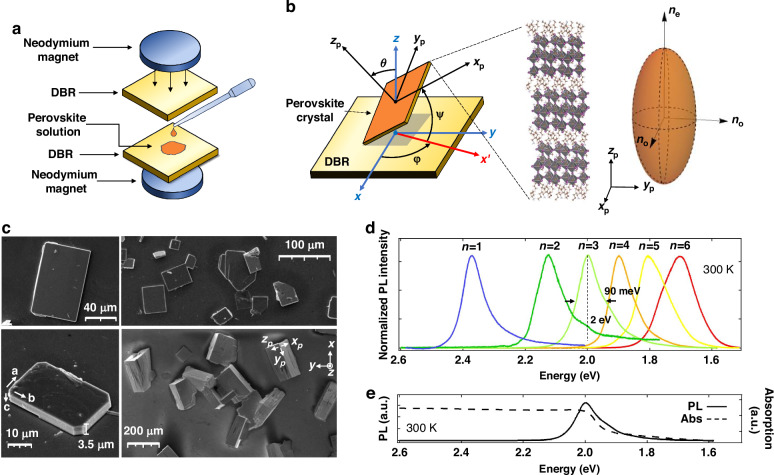
Microcavities with embedded perovskite crystals. **a** The fabrication process involves drop casting a heated perovskite supersaturated solution between two DBRs. **b** The crystals acquire a random orientation with respect to the DBRs surface. The rotation of their crystallographic system $({x}_{p},{y}_{p},{z}_{p})$ relative to that of the cavity $\left(x,y,z\right)$ is described by the Euler angles $(\theta ,\varphi ,\psi )$. The crystal structure is layered and anisotropic, with the ${x}_{p}$ and ${y}_{p}$ plane axes exhibiting an ordinary refractive index ${(n}_{o})$, while the stacking axis ${z}_{p}$ has an extraordinary refractive index ${(n}_{e})$. **c** SEM images of the cavity grown perovskite crystals show widths of about 4 μm and lateral dimensions ranging from a few micrometers to 100 μm. Right-down image of free grown crystals with larger dimensions demonstrating their 3D orientation. **d** PL spectra of perovskite crystals with various inorganic layer number $(n=1-6)$ at room temperature. As $n$ increases, the excitonic emission redshifts to higher wavelengths. **e** Absorption and PL emission of the $n=3$ perovskite compound

**Fig. 2:**
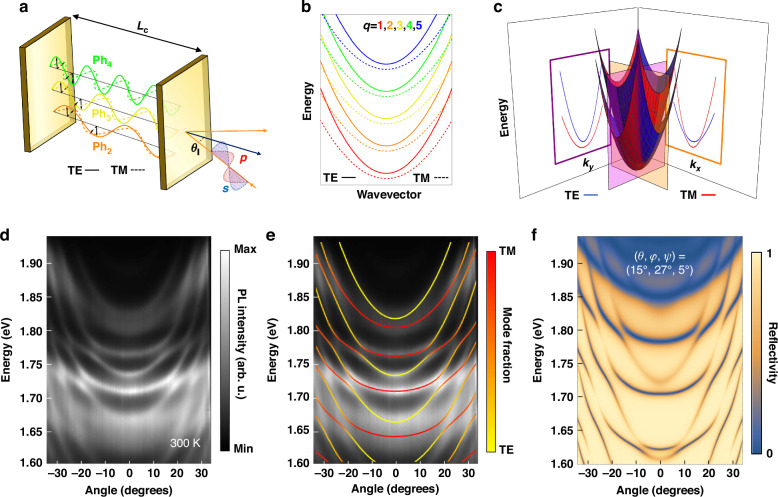
Interaction of photonic modes in a microcavity. **a** Schematic of confined optical cavity modes in a microcavity. **b** Dispersion of multiple photonic cavity modes, with quantum number $q=1-5$, subject to TE-TM and XY splitting. **c** Calculated interaction of two modes with different polarization and opposite parity. Along the ${k}_{y}$ axis for ${k}_{x}=0$, the modes do not intersect, while along the ${k}_{x}$ axis for ${k}_{y}=0$, they exhibit anti-crossing. **d** Angle-resolved photoluminescence of MC1 showing the anti-crossing of multiple cavity modes with RD interaction at room temperature. **e** Simulation of the interacting cavity modes from (**d**) as described by the effective Hamiltonian Eq. (1). **f** Transfer matrix simulation of the reflectivity, showing the same interactions along with the calculated crystal Euler angles

The ${\rm{Si}}{{\rm{O}}}_{2}/{{\rm{Ta}}}_{2}{{\rm{O}}}_{5}$ DBR mirror based microcavities are fabricated by drop casting hot, supersaturated solutions containing halide perovskite precursors, between the DBRs, that are subsequently pressed uniformly by permanent magnets (Fig. 1a). The chosen process involves casting of the homologous perovskite series ${\left({\rm{BA}}\right)}_{2}{\left({\rm{MA}}\right)}_{n-1}{{\rm{Pb}}}_{n}{{\rm{I}}}_{3n+1}$ with $n$ denoting the number of ${{\rm{Pb}}}_{n}{{\rm{I}}}_{3n+1}$ layers separated by organic butylammonium (${{\rm{BA}}}^{+}$) cations and templated from methylammonium (${{\rm{MA}}}^{+}$) cations, mimicking an artificial multiple quantum well structure. The cavity thickness depends on the magnets field strength, resulting in widths of a few μm. As the solution cools down, anisotropically oriented perovskite crystals, defined by Euler angles $(\theta ,\varphi ,\psi )$, are formed (Fig. 1b, c), progressively growing to conform to the shape and dimensions of the confining cavity and the requirements of the crystallographic space group. The lateral dimensions of the crystals span from a few micrometers to 100 μm (Fig. 1c). The large birefringence of these crystals is confirmed by density functional theory simulation, with a disparity ratio of $\frac{\Delta n}{{n}_{o}}=\frac{{n}_{o}-{n}_{e}}{{n}_{o}}=0.48$ at $1.91\,{\rm{eV}}$ between the ordinary $({n}_{o})$ and extraordinary ${(n}_{e})$ refractive indices for the $n=3$ compound (Fig. 1b). Notably, depending on the initial perovskite precursor solution concentrations, several different crystals with $n=\mathrm{1,2},\ldots 6$ can be formed with their emission wavelength spanning from $1.69\,{\rm{eV}}$ to $2.36\,{\rm{eV}}$ as shown in photoluminescence (PL) spectra in Fig. 1d. For our studies we choose the $n=3$ compound, emitting at 2 eV with linewidth $\sim 90\,{\rm{meV}}$ (see SM S.2 for more detailed optical characterization). Two distinct microcavities were fabricated, namely MC1 and MC2, with 6 μm and 4 μm cavity lengths, respectively. Owing to their relatively large thickness, they support multiple cavity modes of even and odd parity quantum number $q$ (Fig. 2a, SM S.4B, S.5).

#### Coupling of optical modes due to the effective spin-orbit coupling

For non-zero angle of incidence, polarization-dependent boundary conditions induce *k*-dependent TE-TM (s-p polarization) splitting and distinct dispersion curves for modes with different polarizations. Additionally, the significant size of the perovskite crystals, combined with their orientation and inherent birefringence, introduces anisotropy. This anisotropy causes different polarizations of light to experience different effective refractive indices, leading to a further TE-TM-like splitting and a notable shift in the normal incidence energy of their dispersion curves, a phenomenon known as XY splitting (SM S.5).

The interplay of this TE-TM-like splitting (henceforth referred to as simply TE-TM) and XY splitting can result in coupling and anti-crossing between cavity modes of different polarization and opposite parity quantum number $q$, leading to the emergence of a distinct spin texture, reminiscent of RD SOC effects (Fig. 2b, SM S.5). Notably, for several wavelength sized cavities, such energy degeneracy between different parity modes is only achievable for materials with a giant birefringence such as the perovskite crystals used in this study. Figure 2c shows the simulated dispersion and anti-crossing of two cavity modes with different polarization and subsequent $q$ number, in a microcavity with similar characteristics to MC1. The dispersion profile is asymmetric in the momentum plane. Along the ${k}_{y}$ axis (for ${k}_{x}=0$) the modes appear uncoupled, while along ${k}_{x}$ axis (for ${k}_{y}=0$) they show anti-crossing. For a general birefringent cavity—where the perovskite crystals are randomly oriented—the system can be described by an effective Hamiltonian in the circular polarization basis given by (see also SM S.9):

1 $${H}_{{ph}}=\mathop{\overbrace{\left(\frac{{\hslash }^{2}{k}_{x}^{2}}{2{m}_{x}}+\frac{{\hslash }^{2}{k}_{y}^{2}}{2{m}_{y}}+\frac{{\hslash }^{2}{k}_{x}{k}_{y}}{2{m}_{{xy}}}\right)I}}\limits^{{\rm{K}}{\rm{inetic\; energy}}}+\mathop{\overbrace{\left({\delta }_{x}{k}_{x}^{2}+{\delta }_{y}{k}_{y}^{2}+{\delta }_{{xy}}{k}_{x}{k}_{y}\right){\hat{\sigma }}_{x}}}\limits^{{\rm{T}}{\rm{E}}-{\rm{TM\; splitting}}}-\mathop{\overbrace{2\left({a}_{x}{k}_{x}+{a}_{y}{k}_{y}\right){\hat{\sigma }}_{z}}}\limits^{{\rm{R}}{\rm{D\; SOC}}}+\mathop{\overbrace{\frac{1}{2}\left({E}_{X}-{E}_{Y}\right){\hat{\sigma }}_{x}}}\limits^{{\rm{X}}{\rm{Y}}{\rm{splitting}}}$$

The first term represents the kinetic energy of a photon inside the cavity, while the second term describes the quadratic dependence of the TE-TM splitting on the wavevector. The third term embodies the RD SOC for each momentum axis, and the last term encapsulates the XY splitting. The effective photon masses are denoted by ${m}_{x}$, ${m}_{y}$ and ${m}_{{xy}}$, while ${\delta }_{x}$, ${\delta }_{y}$, and ${\delta }_{{xy}}$ determine the magnitude of the TE-TM splitting. The parameters ${a}_{x}$ and ${a}_{y}$ indicate the strength of the RD coupling, while ${E}_{X}$ and ${E}_{Y}$ are the zero-momentum energies for modes with linear polarization along the $x$ and $y$ axes, respectively. Here, we generalize the Hamiltonian, with the introduction of two RD parameters ${a}_{x}$ and ${a}_{y}$, instead of the single parameter $a$ used in previous works^31–33,38,44,45^, to account for the 3D orientation of the crystals inside the microcavity. All parameters can be calculated from the cavity length (${L}_{c}$), the elements of the dielectric tensor (${\varepsilon }_{{xp}}$, ${\varepsilon }_{{yp}}$, ${\varepsilon }_{{zp}}$) and the orientation angles ($\theta ,\varphi ,\psi$) of the perovskite crystals, which vary across different points on the sample. Consequently, each crystal exhibits unique effects and responses to incident light, depending on its specific properties. This constitutes an important peculiarity of perovskite microcavities as compared to conventional GaAs-based microcavities. The flexibility of engineering spin-orbit interactions by probing different spots at the surface of the samples offers an important advantage of perovskite-based structures for spinoptronic applications.

Angle-resolved PL of MC1 at room temperature (Fig. 2d, e) shows a broad emission arising from perovskite crystal defect states near its edges^54^, due to degradation of the perovskite crystals filtered by the microcavity modes. The relatively large cavity thickness accommodates a series of cavity modes within the stopband, whereas its pronounced anisotropy leads to significant TE-TM and XY splitting and multiple mode anti-crossings that, in the absence of excitonic emission, are solely due to RD SOC.

Using the effective Hamiltonian (Eq. 1), the dispersion of the cavity can be simulated and fitted with a coupled oscillator model (Fig. 2e). The coupling between these modes at the anti-crossing points leads to their hybridization and a mixing of their polarization. Using a transfer matrix method, similar to the Berreman model (SM S.10), the orientation of the crystal at this point of the microcavity can be calculated through its Euler angles as $\theta =15^{\circ} ,\,\varphi =27^{\circ} ,\,\psi =5^{\circ}$ with respect to the DBR surface (Fig. 2f).

#### Switching on the strong coupling regime

The integration of a resonant media within the cavity couples the excitonic and photonic modes. Photonic modes with opposite parity and different polarization can couple not only with each other (RD coupling) but also with the exciton, producing polaritonic states (strong coupling). The total Hamiltonian of the system, including strong coupling and RD coupling is presented in SM S.5C.

**Fig. 3:**
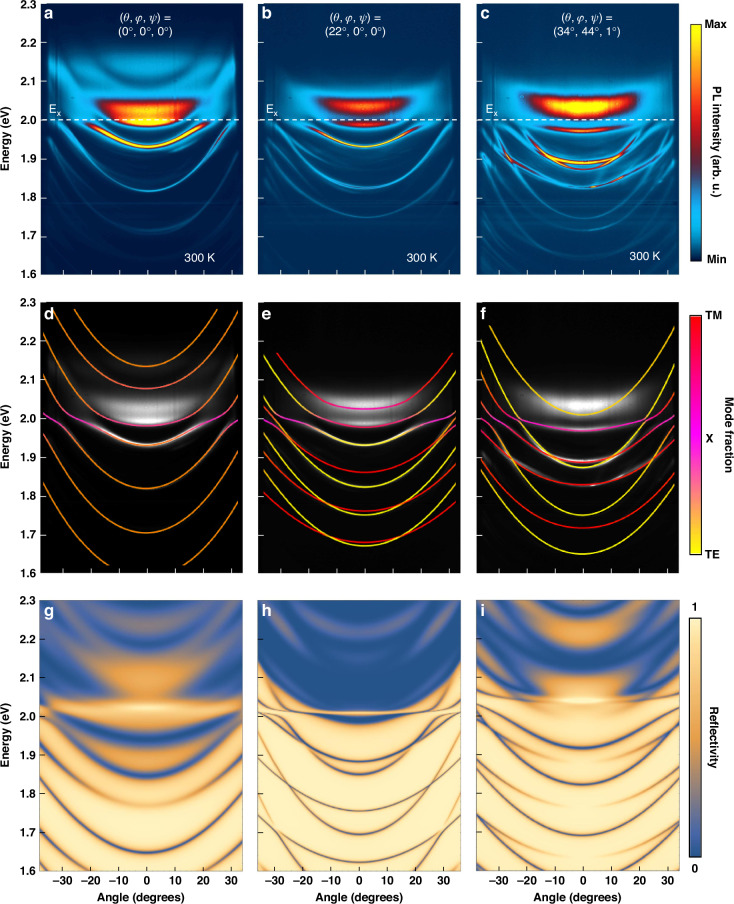
Combined strong coupling and RD SOC. **a**–**c** Angle-resolved photoluminescence at room temperature for three crystals with different orientation in MC2. Many anti-crossings and interactions are visible due to the two main effects, namely strong light-matter coupling and RD SOC. **d**–**f** Dispersion simulations of the three sample points using a coupled oscillator model and the total effective Hamiltonian of the system. The simulations reveal maximum Rabi splittings of $97\,{\rm{meV}}$, $64\,{\rm{meV}}$ and $60\,{\rm{meV}}$, respectively. **g**–**i** Reflectivity calculated with a 4 × 4 transfer matrix model, revealing the crystal orientation (Euler angles) of the three crystals

The PL emission of MC2 at room temperature (Fig. 3) shows a clear excitonic resonance at 2 eV (E_x_), consistent with the PL measurements of bare crystals with layer number $n=3$. Notably, the emission is very close to the edge of the stopband and the first Bragg mode of the DBRs at around 2.03 eV. Angle-resolved PL measurements at three different points in the microcavity (Fig. 3a–c) reveal multiple mode couplings stemming from polaritonic strong coupling, typically marked by sharper anti-crossings, RD coupling occurring far from the excitonic emission, or a combination of both effects.

Utilizing the transfer matrix method (Fig. 3g–i) in conjunction with the coupled oscillator model derived from the total system Hamiltonian (Fig. 3d–f), we have identified and simulated the properties of the specific crystal points shown in the dispersion images. Figure 3a, d, g shows indistinct TE-TM and XY splittings indicating the presence of solely strong coupling between the cavity modes and the perovskite exciton, with the crystals lying on the plane, i.e., oriented at $\theta =0^{\circ} ,\,\varphi =0^{\circ} ,\,\psi =0^{\circ}$ Euler angles. The polariton branches and the uncoupled cavity modes are accurately fitted, revealing a maximum Rabi splitting of $\sim 97\,{\rm{meV}}$ close to the exciton resonance. Conversely, at the positions illustrated in Fig. 3b, e, h and Fig. 3c, f, i, the splittings and energy shifts between the different polarizations are more pronounced. Photonic and polaritonic modes with the same parity cross, whereas modes of different polarization and opposite parity interact, exhibiting characteristic anti-crossings. The perovskite exciton mode further influences the curvature of the optical modes near its resonance, forming polaritonic branches with Rabi splittings of $\sim 64\,{\rm{meV}}$ for the point shown in Fig. 3b, and $\sim 60\,{\rm{meV}}$ for the point in Fig. 3c, respectively. The transfer matrix simulations have determined the crystal orientations for these two points to be $\theta =22^{\circ} ,\,\varphi =0^{\circ} ,\,\psi =0^{\circ}$ and $\theta =34^{\circ} ,\,\varphi =44^{\circ} ,\,\psi =1^{\circ}$, respectively. These findings indicate that the splittings and the photonic SOC effects become more pronounced at higher crystal angles as the anisotropy of the system increases.

#### Effective magnetic field

In the OSHE, polarized light interacts with a material characterized by a wavevector-dependent effective magnetic field induced by the TE-TM splitting. In our case, the combination of TE-TM and XY splittings along with the RD-like interaction arising from the microcavity anisotropy, can be expressed as the sum of two wavevector-dependent effective magnetic fields ${B}_{{TE}-{TM},{XY}}$ and ${B}_{{RD}}$, acting on the pseudospin of light (polarization). Although these fields coexist inside the microcavity, they may cancel each other at certain momenta, corresponding to specific points in the momentum plane known as *diabolical points* (DP).

Our effective Hamiltonian can be decomposed into a linear combination of Pauli matrices, representing a Zeeman-like interaction between the photon pseudospin and the effective magnetic field. This way, the effective Hamiltonian and the associated momentum-dependent effective magnetic field can be written in the circularly polarized basis as follows (SM S.6):

2 $${H}_{{ph}}=\left(\frac{{{{\hslash }}}^{2}{k}_{x}^{2}}{2{m}_{x}}+\frac{{{{\hslash }}}^{2}{k}_{y}^{2}}{2{m}_{y}}+\frac{{{{\hslash }}}^{2}{k}_{x}{k}_{y}}{2{m}_{{xy}}}\right)I+{\mu }_{{\rm B}}g{\boldsymbol{B}}\,\cdot\, {\boldsymbol{\sigma }}$$

3 $${\boldsymbol{B}}=({\delta }_{x}{k}_{x}^{2}+{\delta }_{y}{k}_{y}^{2}+{\delta }_{{xy}}{k}_{x}{k}_{y}+\frac{1}{2}\left({E}_{X}-{E}_{Y}\right),0,\,-2\,{a}_{x}{k}_{x}-2\,{a}_{y}{k}_{y}\,)$$

**Fig. 4:**
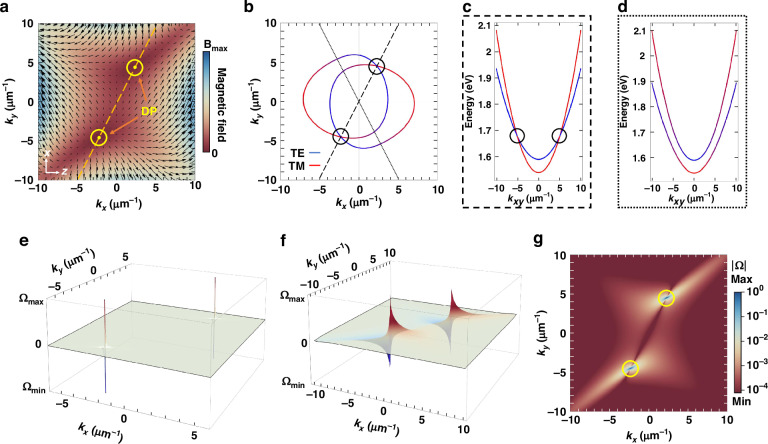
Effective magnetic field, 2D dispersion, Berry curvature, and diabolical points. **a** Total effective magnetic field for the system of two interacting cavity modes of Fig. 2c. The diabolical points (DP) occur where the magnetic field is cancelled and are positioned on a line on the ${k}_{x},{k}_{y}$ plane with a slope proportional to ${a}_{x}/{a}_{y}$. **b** Cross-section of the simulated dispersion from Fig. 2c. **c** Energy dispersion along the line connecting the diabolical points (${k}_{{xy}}$ on the dashed line). The modes do not interact at the diabolical points and cross. **d** Energy dispersion along a line mirrored on the ${k}_{y}$ axis (${k}_{{xy}}$ on the dotted line) where the two modes anti-cross. **e** Calculated Berry curvature $\Omega$ showing two ill-defined divergent points for momentum values of the DP. **f** Same depiction as (**e**) at a logarithmic scale. **g** Absolute value of the Berry curvature on the ${k}_{x}$, ${k}_{y}$ plane, illustrating the divergence at the DP

For MC1 and the two interacting modes of Fig. 2c, the parameters of our calculation are ${a}_{x}=-4.006\,{\rm{meV}}{{\upmu }}{\rm{m}}$, $\,{a}_{y}=2.039\,{\rm{meV}}{{\upmu }}{\rm{m}}$, ${\delta }_{x}=1.622\,{\rm{meV}}{{{\upmu }}{\rm{m}}}^{2}$, ${\delta }_{y}=-1.897\,{\rm{meV}}{{{\upmu }}{\rm{m}}}^{2}$, ${\delta }_{{xy}}=-0.004\,{\rm{meV}}{{{\upmu }}{\rm{m}}}^{2}$ and ${E}_{X}-{E}_{Y}=46\,{\rm{meV}}$. The resulting theoretically calculated effective magnetic field is shown in Fig. 4a.

Diabolical points emerge at specific planar momentum values of $({k}_{x},{k}_{y})=\pm \left(2.02,\,3.97\right)\,{{{\upmu }}{\rm{m}}}^{-1}$, where the vector sum of the two fields ${B}_{{TE}-{TM},{XY}}$ and ${B}_{{RD}}$ is zero (see SM S.6). Interestingly, unlike previous works^35,38,41,45^ where the DP occur only parallel to the ${k}_{x}=0$ or ${k}_{y}=0$ direction, in our system, they appear along ${k}_{y}=-({a}_{x}/{a}_{y}){k}_{x}$. Figure 4b shows the simulated cross-section of the interacting modes dispersion at the energy corresponding to the DP values of planar momentum. At the DP, the modes merely cross without any interaction (Fig. 4c). Conversely, at points mirrored on the ${k}_{y}$ axis, the modes anti-cross and display maximum interaction (Fig. 4d). At these points, the transverse magnetic field ${B}_{x}$, corresponding to TE-TM and XY splitting, is zero due to its inversion symmetry. However, ${B}_{z}$, corresponding to RD interaction, is only zero across the dashed line of Fig. 4b, leading to a non-zero contribution to the effective magnetic field in other directions. Along the dotted line of Fig. 4b, and at the points mirrored to the diabolical with respect to the ${k}_{y}$ axis, the two eigenenergies of the Hamiltonian are ${E}_{\pm }={E}_{i}\pm \sqrt{{B}_{z}^{2}}$, resulting in the gap opening with width of ${2{|B}}_{z}|$ due to the non-zero inversion-asymmetric effective magnetic field and RD SOC.

The distinctive warped dispersion cross section shown in Fig. 4b, differs from the circular one observed in other RD microcavity systems^31,33,35,39–41,44^, and originates from the presence of two RD terms in the effective Hamiltonian due to tilting of the perovskite crystals inside the microcavity. This is analogous to the trigonal warping of the energy bands in certain two-dimensional materials due to SOC and asymmetric structure^55,56^.

#### Berry curvature

When a quantum system evolves around a closed path in parameter space that encircles a DP, a non-Abelian geometric phase can accumulate, associated with the topological changes of the parameter space and closely related to concepts like the Berry curvature. The Berry curvature can become singular or exhibit unusual behavior depending on the specific characteristics of the system and the geometry of the DP. The presence of non-zero Berry curvature arising from material anisotropy can have consequences for topological invariants and non-trivial topological transitions, leading to the generation of artificial non-Abelian gauge fields for light.

The calculated Berry curvature $\Omega$ for two interacting modes of Fig. 2c and Fig. 4a–d (SM S.7), is presented in Fig. 4e–g. The Berry curvature is asymmetric with respect to the ${k}_{x}$ axis, an odd function in the momentum plane and has two ill-defined divergent values for two specific points in the momentum plane. These points are indeed the DP, with $\Omega \to +\infty$ for ${k}_{x}=2.02\,{{{\upmu }}{\rm{m}}}^{-1}$ and ${k}_{y}=3.97\,{{{\upmu }}{\rm{m}}}^{-1}$ and $\varOmega \to -\infty$ for ${k}_{x}=-2.02\,{{{\upmu }}{\rm{m}}}^{-1}$ and ${k}_{y}=-3.97\,{{{\upmu }}{\rm{m}}}^{-1}$.

Similar to the position of the DP, the Berry curvature dipole direction is determined by the relative magnitude of the two RD contributions, namely the ${a}_{x}/{a}_{y}$ ratio. This in turn is defined by the Euler angles $\theta ,\,\varphi ,\,\psi$ and the rotation of the crystal. The rotation angles dependence of the photonic interaction, diabolical points position and Berry curvature characteristics are presented in Figure S.29 and Supplementary Video 1. Notably, the line along which the DP occur separates the momentum plane in two regions of opposite Berry curvature. Although the opposite sign Berry curvature in the two regions indicates that time-reversal symmetry is conserved, a gap-opening with width of ${2{|B}}_{z}|$ is still possible due to the combined effect of TE-TM and RD contributions^38^.

## Discussion

It is instructive to compare the polariton spin-orbit effects revealed in this study to those addressed in the recent works on liquid-crystal based microcavities^31,32,44,45^. Both systems are promising for the engineering of effective spin-orbit Hamiltonians. The control of the coupling of photonic modes of different polarizations can be achieved in liquid crystal microcavities by tuning of the external bias. In perovskite microcavities, by tuning the orientation of perovskite crystals with respect to the axis of the planar microcavity structure one can achieve a similar degree of control over spin-orbit effects. The fundamental difference between the two systems is in the impact of exciton-photon coupling. Liquid crystals exhibit no exciton resonances themselves and in order to achieve lasing they are used in combination with other emissive material such as perovskite crystals. Dye-filled liquid crystal cavities have been used^32^ but the polariton lasing, Bose-Einstein condensation and superfluidity of exciton-polaritons are yet to be demonstrated in these structures. In contrast, pure perovskite microcavities are highly suitable for the observation of strong exciton-photon coupling and lasing effects, which makes them excellent candidates for the realization of neuromorphic and even quantum computation devices^57^.

In conclusion, by using a series of birefringent layered 2D hybrid perovskite crystals as the active medium, providing both high degree of anisotropy and strong excitonic emission, we have achieved the strong coupling regime in microcavities with a Rabi splitting of ~$100\,{\rm{meV}}$, paired with photonic RD SOC effects at room temperature. Our solution-based method facilitates the fabrication of microcavities that support multiple confined photonic modes due to their relatively large width. Moreover, the orientation of the self-assembled crystals inside the microcavities is found to play a major role in the emergence of RD effects and associated splittings, with large crystal orientation angles producing more pronounced interactions and a variety of dispersion features (crossings, anti-crossings, mode polarization hybridization). We describe our 2D hybrid perovskite microcavity by means of a generalized effective Hamiltonian which, unlike previous works, is formulated for a fully anisotropic crystal at random three-dimensional orientation. This allows us to calculate topological features such as the effective magnetic field and Berry curvature and reveal non-trivial characteristics such as the asymmetric emergence of diabolical points in the momentum space.

The integration of topological photonics with the described microcavity perovskite system could lead to a plethora of potential applications. The ability to engineer topologically protected states in photonic systems opens up new avenues for robust optical communication and quantum information processing, with unidirectional light transport immune to scattering and defects^16,18,19^. Additionally, the realization of artificial gauge fields in our microcavity system paves the way for novel photonic devices such as non-reciprocal optical components, which can be crucial for integrated photonic circuits^34,58^ and the development of advanced light sources, including topological non-reciprocal lasers that can operate with high efficiency and stability under ambient conditions^59^. These innovations could not only enhance the functionality of existing photonic technologies but also enable entirely new paradigms in the topological manipulation and control of light.

## Materials and methods

### Optical characterization

For the optical characterization of the perovskite crystals and fabricated microcavities an excitation source consisting of a mode-locked ultrafast Ti: Sapphire laser, pumped by a solid-state diode-pumped frequency-doubled Nd: Vandate $532\,{\rm{nm}}$ $10\,{\rm{W}}$ is used, producing pulses tuned at $800\,{\rm{nm}}$ with repetition rate $75.25\,{\rm{MHz}}$, temporal width $\sim 125\,{\rm{fs}}$ and pulse energy $\sim 17\,{\rm{nJ}}$. Initially, the frequency of the light pulses is doubled by means of a $1\,{\rm{mm}}$ beta barium borate crystal to $400\,{\rm{nm}}$. The remaining fundamental is completely removed using a bandpass filter centered at $495\,{\rm{nm}}$. Lastly, the pulsed beam is focused into a 3 μm spot on the sample by a $0.55\,{\rm{NA}}$, $13\,{\rm{mm\; WD}}$ Plan Apochromat Objective Lens while its power is adjusted by a continuously variable neutral density filter. The PL emission of the samples is filtered from the excitation beam by a $455\,{\rm{nm}}$ longpass filter and is directed into a spectrometer where it is spectrally analyzed. The angle-resolved PL measurements are acquired through capturing the Fourier plane signal by placing lenses at a distance equal to their focal length from the objective lenses.

### Synthesis of perovskite solution and crystals

The two-dimensional Ruddlesden–Popper perovskite crystal thin sheets are synthesized using an interfacial growth method with the precursor species, concentrations, and ratios carefully optimized and the crystallization temperature carefully controlled to promote layer-by-layer growth, avoid dislocation formation, maximize lateral growth and maintain phase purity. For the ${\left({\rm{BA}}\right)}_{2}{\left({\rm{MA}}\right)}_{n-1}{{\rm{Pb}}}_{n}{{\rm{I}}}_{3n+1}$ series and specifically the ${\left({\rm{BA}}\right)}_{2}{\left({\rm{MA}}\right)}_{2}{{\rm{Pb}}}_{3}{{\rm{I}}}_{10}$ ($n=3$) compound (with ${\rm{BA}}$ and ${\rm{MA}}$ abbreviations for n-butylammonium and methylammonium, respectively), the synthesis is described below:

Lead iodide (${\rm{Pb}}{{\rm{I}}}_{2}$) ($0.59{\rm{M}}$) and methylammonium chloride (${{\rm{CH}}}_{3}{{\rm{NH}}}_{3}{\rm{Cl}}$) ($0.40{\rm{M}}$) precursors are dissolved in a concentrated aqueous solution of hydrogen iodide (${\rm{HI}},\,57 \% \frac{{\rm{w}}}{{\rm{w}}}$ in ${{\rm{H}}}_{2}{\rm{O}}$) and hypophosphorous acid (${{\rm{H}}}_{3}{{\rm{PO}}}_{2},\,50 \% \frac{{\rm{w}}}{{\rm{w}}}$ in ${{\rm{H}}}_{2}{\rm{O}}$) mixture ($10:1\frac{{\rm{vol}}}{{\rm{vol}}}$) and then heated at 130 °C in a closed stirred glass vial until a clear, yellow solution is obtained. In a separate beaker, n-butylamine (${\rm{n\hbox{-}}}{{\rm{CH}}}_{3}{\left({{\rm{CH}}}_{2}\right)}_{3}{{\rm{NH}}}_{2}$) ($0.19M$) is neutralized with ${\rm{HI}}$ $57 \% \frac{{\rm{w}}}{{\rm{w}}}$ in ${{\rm{H}}}_{2}{\rm{O}}$ ($2\,{\rm{mL}}$) resulting in a clear pale-yellow solution and then is added to the ${\rm{Pb}}{{\rm{I}}}_{2}$ solution slowly. The solution is then cooled down to 65 °C and kept at that temperature in a closed vial in a sand bath as the stock solution. 2 μL of this warm supernatant solution are collected and dispensed with a 10 μL pipette onto a glass slide placed in an open ambient environment (25 °C). Nucleation and growth quickly initiate on the surface of the precursor solution droplet and perovskite thin sheets floating on the droplets are obtained within a few seconds up to 30 s.

### Microcavities fabrication

For our studies, we have synthesized two kinds of microcavities, consisting of perovskite crystals sandwiched between DBRs. MC1 microcavity consists of 10 layers of ${{\rm{Ta}}}_{2}{{\rm{O}}}_{5}/{\rm{Si}}{{\rm{O}}}_{2}$ (starting with ${{\rm{Ta}}}_{2}{{\rm{O}}}_{5}$) on a sapphire substrate, with an extra top layer of ${{\rm{Ta}}}_{2}{{\rm{O}}}_{5}$, and stop-band centered at $700\,{\rm{nm}}\,(1.77\,{\rm{eV}})$. MC2 microcavity consists of the same structure but on a quartz substrate and stop-band centered at $650\,{\rm{nm}}\,(1.91\,{\rm{eV}})$. Initially, the DBRs are thoroughly cleaned with acetone for the removal of any organic dirt. Then the bottom DBR is placed on an aluminum stand heated by a hot plate at around 80 °C. The perovskite solution is also heated and stirred at 100 °C for about $5\,\min$ until all the pre-crystals are dissolved inside the solvent. Then, 5 μL of the solution is quickly drop-casted on the hot DBR before the new crystals start to form. Lastly, the sample is covered with the top DBR, that is again heated at 80 °C, and another aluminum plate. The cavity is then removed from the hot plate and placed between two permanent ring-shaped magnets that firmly hold together the DBRs with uniform force. The whole structure is left to cool down and after 2 days inside a glove box with a nitrogen atmosphere, several micrometers wide crystals are grown inside the microcavity. Then the magnets are carefully detached and the surface of the sample is cleaned from residual material/solvent using acetone.

## Supplementary information

Supplementary Material

Supplementary Video 1

Supplementary Video 2

## Data Availability

The data that support the findings of this study are available from the corresponding author upon reasonable request.
