# Supplementary material for: Unveiling asymmetric topological photonic states in anisotropic 2D perovskite microcavities
Source: Light Sci Appl. 2025 May 29;14:207. doi: 10.1038/s41377-025-01852-8 (PMC12122894; doi:10.1038/s41377-025-01852-8)
Supplement: Supplementary file 1 — Supplementary Material [file 41377_2025_1852_MOESM1_ESM.pdf]

Supplementary Material for

Unveiling Asymmetric Topological Photonic States in Anisotropic 2D Perovskite Microcavities

E. G. Mavrotsoupakis, L. Mouchliadis, JH. Cao, M. C. Chairetis, M. E. Triantafyllou-Rundell, E. C. P. Macropulos, G. G. Paschos, A. Pantousas, HY. Liu, A. V. Kavokin, H. Ohadi, C. C. Stoumpos, P. G. Savvidis

Contents

Part A. Additional measurements and theory 3
S.1 Overview of Halide Perovskite Microcavities 3
S.2 Optical characterization of perovskite crystals 4
S.2.A Optical spectroscopy set up 4
S.2.B Microscope and SEM images 5
S.2.C Emission and reflectivity of crystals 11
S.2.D Temperature dependence 15
S.2.E Power dependence 17
S.2.F Time resolved measurements 19
S.2.G Birefringence 20
S.3 Perovskite crystal synthesis and microcavity fabrication 22
S.3.A Synthesis of perovskite solution and crystals 22
S.3.B Microcavities Fabrication 24
S.4 Microcavities properties 25
S.4.A Distributed Bragg Reflectors 25
S.4.B Optical cavity modes 26
S.5 Splitting and interaction of cavity modes and strong coupling 31
S.5.A Rashba-Dresselhaus interaction 31
S.5.B Polaritonic strong coupling 33
S.5.C. Total effective Hamiltonian 35
S.6 Effective magnetic field 35
S.7 Berry curvature calculation 37
S.8 Other effects 39
S.8.A Polarization dependence 42
S.8.B Confinement effects 45
Part B. Hamiltonian and Transfer matrix calculation 47
S.9 Derivation of the generalized Rashba-Dresselhaus Hamiltonian for a birefringent microcavity 47
S.10 Transfer matrix method for a birefringent cavity 71
S.11 Supplementary Videos 75
References 75

Part A. Additional measurements and theory

S.1 Overview of Halide Perovskite Microcavities

Hybrid organic-inorganic halide perovskite crystals are direct bandgap semiconductors having the 3D bulk structure expressed by the formula $ABX_3$where A is a monovalent organic cation, B is a divalent metal cation, and X is a halide anion. Their 2D variant features multilayered stacks of n inorganic layers separated by a bilayer of intercalated long chain organic cation.

This formation resembles multiple quantum well (MQW) structures, where the inorganic layer acts as the potential well and the organic part as the barrier. These 2D perovskites showcase excellent optical and electronic properties, blending organic and inorganic benefits [46-47, 54, 60, 62]. The strong quantum and dielectric confinement provide a large exciton binding energy, oscillator strength, and photoluminescence quantum yield at room temperature, as well as a large exciton diffusion length, lifetime and nonlinear interaction strength. Their self-assembled nature simplifies fabrication with soft chemistry methods and their emission can be tuned across ultraviolet to near-infrared by precisely adjusting the thickness of the well and barrier sheets, making them ideal for photovoltaics, optoelectronics, photonics and quantum information [48, 50, 63-66].

The basic concepts were large developed in the early 1990s from the pioneering work of Papavassiliou [67], Mitzi [68] and Ishihara [69] who established the fundamental aspects of the materials, with the latter being the first to attempt to exploit them in exciton-polariton microcavities [70-71]. The modern view of perovskite exciton-polaritons microcavities has been revolving mainly around the bulk perovskites $CsPbCl_3$ and $CsPbBr_3$ with PEA_2PbI_4 (PEA stand for the cationic form of phenylethylamine) representing the cornerstone of studying polaritons in two dimensional systems. These systems have been shown remarkable results in the demonstration of exciton-polaritons and room temperature Bose-Einstein condensation [72-78], yet these do not make full use of the maximum potential of perovskites which lie in the precise control of the excitonic properties via the employment of the complete homologous n-layered series of the form $A'_2(A)_{n-1}M_nX_{3n+1}$ (Ruddlesden-Popper, RP) or $A'(A)_{n-1}M_nX_{3n+1}$ (Dion-Jacobson, DJ), where A' is a large monovalent (RP) or bivalent (DJ) spacer cation, A is a small "perovskitizer" cation, M is a bivalent group metal ion and X is a halogen anion [49]. An exception to this comes from the study of "bare" (i.e. cavity-less) polaritons obtained recently for a $n = 2$ member of a lead iodide based homologous series [79]. The major advantage of homologous series is that the key parameters of the excitons (binding energy, Bohr radii, etc. [80]) can be finely tuned by varying both the dimensions of the barriers and the wells [81], independently from one another, thus producing a wealth of systems that can be manipulated to produce optimal exciton-polariton strong coupling. Furthermore, these compounds have anisotropic orthorhombic crystal structure that results in intrinsic birefringence. They also exhibit strong spin-orbit coupling (SOC) due to the presence of heavy elements such as Pb, which results in the splitting of conduction and valence bands. The combination of SOC with broken structural inversion symmetry facilitates Rashba-Dresselhaus couplings and topological for photonics, and spintronics [82-86].

These complex systems hold great challenges to overcome since defect-associated phenomena (generically termed "edge-effect" [54]), large optical nonlinearities [87], and symmetry breaking associated effects [82], are all needed to be overcome in order to successfully demonstrate light-matter interactions. Exciton-polaritons emerging from perovskite crystal microcavities can have immense potential in realizing Berry curvature, topological states and photonic artificial gauges with many examples in recent studies [38-37, 39-44].

S.2 Optical characterization of perovskite crystals

S.2.A Optical spectroscopy set up

For the optical characterization of the perovskite crystals and the subsequent fabricated microcavities, as presented in **Figure S.1**, an excitation source consisting of a mode-locked ultrafast Ti: Sapphire laser (Mira Optima 900-F, Coherent), pumped by a solid-state diode-pumped frequency-doubled Nd: Vandate $532\,nm$ $10\,W$ (Verdi V10, Coherent) is used, producing pulses tuned at $800\,nm$ with repetition rate $75.25\,MHz$, temporal width $\sim 125\,fs$ and pulse energy $\sim 17\,nJ$. Initially, the frequency of the light pulses is doubled by means of a $1\,mm$ beta barium borate crystal (Crystech) to $400\,nm$. The remaining fundamental is completely removed using a bandpass filter centered at $495\,nm$ (FSR-BG40, Newport). Lastly, the pulsed beam is focused into a $3\,\mu m$ spot on the sample by a 0.55 NA, $13\,mm$ WD Plan Apochromat Objective Lens (MY50X-805-50X, Mitutoyo) while its power is adjusted by a continuously variable neutral density (ND) filter (Edmund).

The sample is placed either outside, for room temperature measurements, or inside a cryostat (Micro-PL Cryostat DE-204PF-DMX-20-OM, Advanced Research Systems) where its temperature can be lowered down to $13\,K$ in a $10^{-6}\,mbar$ environment. The PL emission of the sample is filtered from the excitation beam by a $455\,nm$ longpass filter (FGL455, Thorlabs) and is directed into a spectrometer (Acton SpectraPro 500 *mm* Imaging Triple Grating Spectrograph, Princeton Instruments) where is spectrally analyzed. In our experiments, we have used two gratings with 150 and $600\,grooves\,mm^{-1}$ blazed at $800\,nm$ and $1\,\mu m$ and an observable window of $350\,nm$ and $80\,nm$, respectively. The angle resolved PL measurements of our samples are acquired through capturing the Fourier plane signal by placing lenses at a distance equal to their focal length from the objective lenses.

The emission signal linear polarizations are separated using a polarizing beam splitter (PBS252, Thorlabs) and a set of mirrors directing them to the spectrometer. Left and right circular polarizations are distinguished by placing a $\lambda/4$ waveplate (WPQ05M-633, Thorlabs) before the beam splitter, transforming them into parallel and orthogonal linear polarizations.

Reflectivity measurements are conducted with the use of a white light, halogen lamp (KLS) and real space images of the sample are captured with a camera (ZWO ASI290MM).

For time resolved measurements we have used a second spectrometer (Acton SpectraPro 300 *mm*) with its output directed into a streak camera system (C5680 with M5675 Synchroscan Unit, Hamamatsu), synchronized with the frequency of the pulsed excitation. The signal is

amplified using MCP plates, operating at around $360\ V$, and is projected on a CCD camera (C4880-16 Dual-Mode cooled CCD, Hamamatsu) for inspection. A time window of around $800\ ps$ is simultaneously observed with a temporal resolution of around $6\ ps$. The streak camera is triggered by delivering a small portion of the initial beam to a p-i-n photodiode (C1808-03, Hamamatsu), operating along with a Delay (C1097-01, Hamamatsu) and Trigger Unit (C4792-01, Hamamatsu).

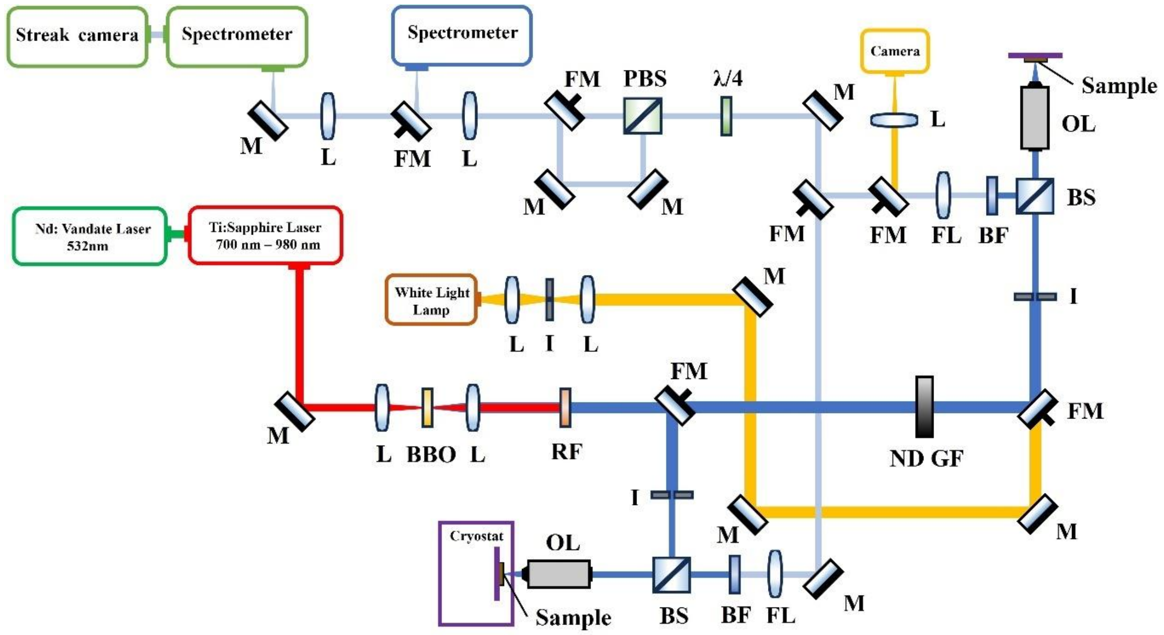

Figure S1: Experimental optical setup. The main excitation ($800\ nm$), pumped by a $532\ nm$ laser source, is frequency doubled to $400\ nm$ by a **BBO** plate. The remaining red excitation is filtered by a bandpass filter (**RF**). The beam can then either excite samples in a cryostat or in an open environment. It is focused on them by objective lenses (**OL**) while its fluence can be adjusted by a ND gradient filter (**ND GF**). The emission signal, after it is cleared of the blue wavelengths by long pass filters (**BF**), is directed to the spectrometers and streak camera. The signal polarizations are separated by a polarizing beam splitter (**PBS**) and a λ/4 waveplate. Angle resolved measurements are acquired by capturing the Fourier plane signal of the sample by placing lenses (**FL**) at specific positions along the collection paths. Reflectivity measurements and images of the samples are obtained with the use of a white lamp source and a camera. Further optical components of the setup are regular (**M**) and flipping mirrors (**FM**), beam splitters (**BS**), lenses (**L**) and irises (**I**).

S.2.B Microscope and SEM images

Drop-cast grown crystals

In **Figure S.2**, optical microscope and SEM images of a drop casted sample of $n = 3$ layered perovskite crystals are presented. The crystals have a bright red color, characteristic of their layer number, and form aggregates that range in in-plane dimensions from $1\ mm$ to $50\ \mu m$. The aggregates result from the merging of different crystals with different crystalline directions as they grow inside the solvent. Single crystals can also form, with a single

orientation of their periodic structure, and average dimensions close to 200 μm. They are normally shaped as rectangular cuboids with an estimated width of 50 μm. Some solvent residue is present on the sample with crystals still flowing inside. Their orientation is random and as they become larger and touch the substrate, they retain this position even after the solvent has evaporated. Similar behavior has been seen in other works, such as in [88].

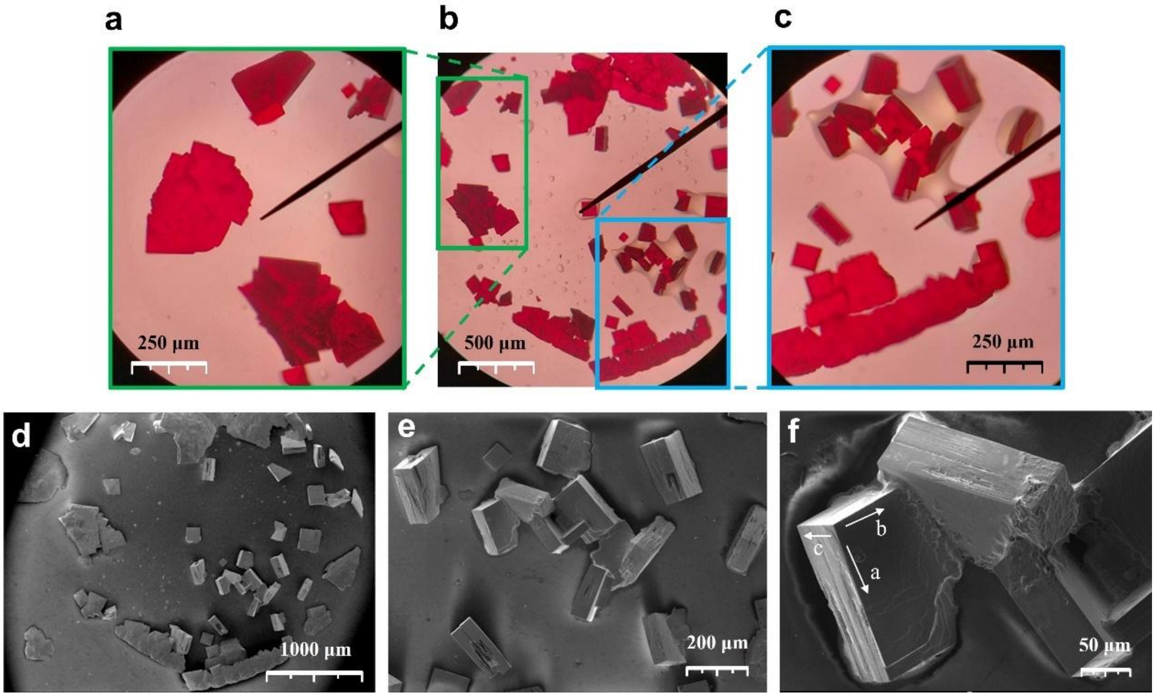

Figure S.2: Microscope and SEM images of $n = 3$ perovskite crystals, drop casted and freely grown on glass substrate. (**a**)-(**c**) Optical microscope images of the crystals with (**a**) and (**c**) being zoomed images of specific regions on the sample (**b**). In (**a**), large aggregates with merged crystals and dimensions close to 1 mm can be observed. Single crystals can also be seen still flowing in solvent with angled positions and dimensions of around 200 μm in (**c**). (**d**)-(**f**) SEM images of the same sample region. In (**e**) and (**f**), the specific rectangular shape following the crystallographic axes $\boldsymbol{a}$, $\boldsymbol{b}$ and $\boldsymbol{c}$ as well as the random orientation of the crystals can be distinguished.

In more detailed images of the single crystals in **Figure S.3**, they are shown to consist of many individual layers, with an average width of 1 μm, stacked together and grown according to their crystallographic directions. Each of these layers contains many individual elementary layers of alternating organic and inorganic cations of a few nm width.

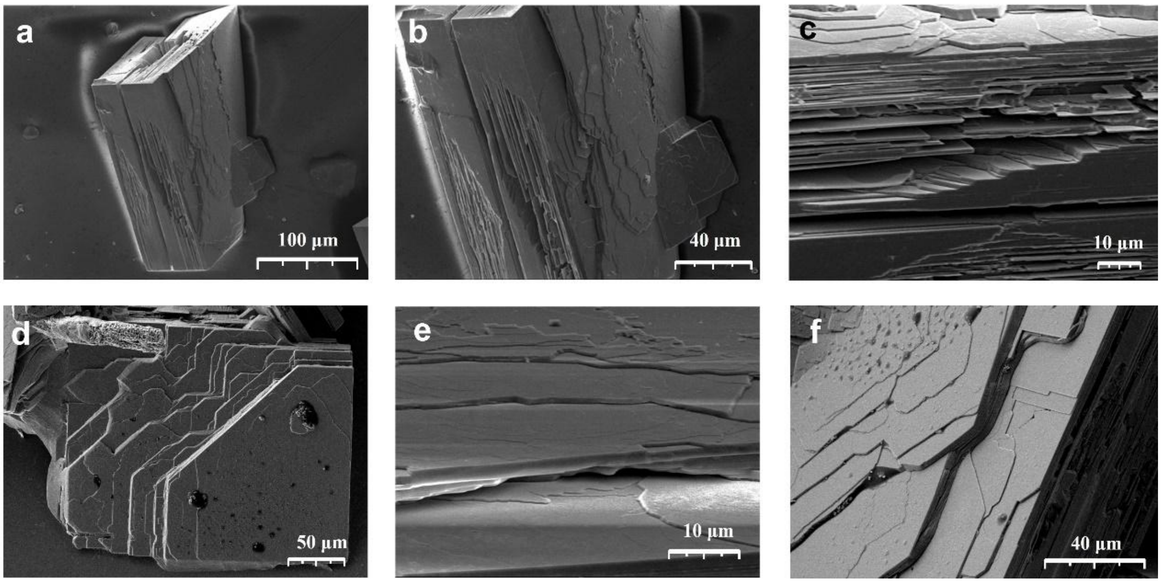

Figure S.3: Detailed SEM images of $n = 3$ single crystals. They are assembled by many layers of about 1 μm width, aligned to the crystallographic directions. Each of these layers are composed of many alternating stacks of inorganic halide perovskites and organic cations of some nm width.

Confined crystals

Images of crystals grown between pressed glasses are also shown in **Figure S.4**. The resulting merged aggregates have increased in-plane dimensions, of many mm in some cases, due to the fact that their growth is mainly allowed laterally, with the perpendicular direction confined by the glasses. The multiple colors and hues represent the different widths and directions the crystals acquire before being combined to larger bodies.

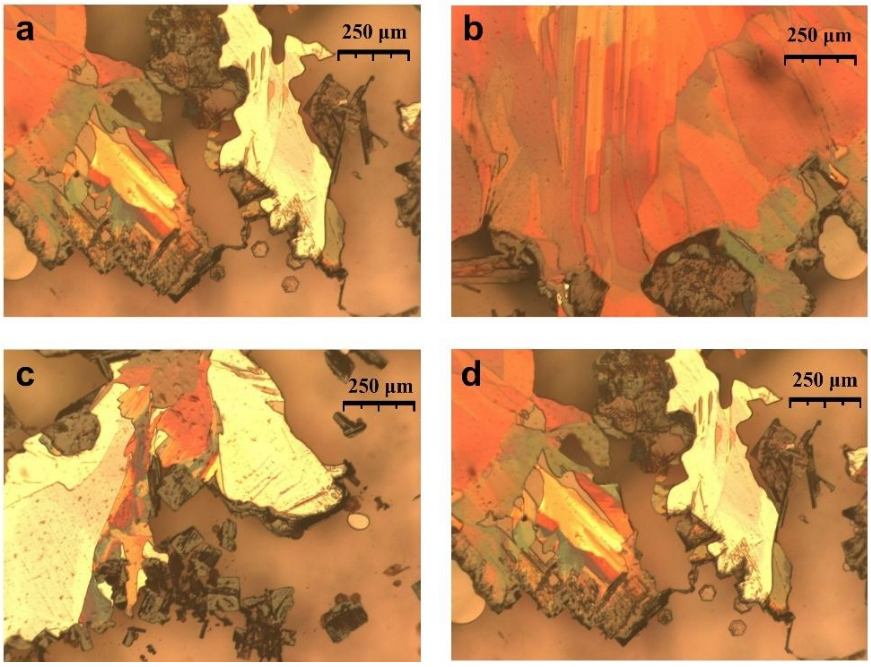

Figure S.4: Optical microscope images of perovskite crystals $n = 3$ grown between pressed glasses. Different single crystals of various orientations and widths are combined together and form large aggregates that can reach even a few mm in size. Different colors and shades indicate the various properties of the aggregate for each point.

Besides large aggregates, many single crystals with distinct rectangular shapes are observed when the crystals are grown in the pressed solvent as shown in **Figure S.5**. These crystals have lateral sizes on the order of $100 - 250\ \mu m$, while their width varies between 1 and $10\ \mu m$. They too grow with random orientations and, when the solvent is removed, some of them are positioned either on top of each other or at an angle to the substrate.

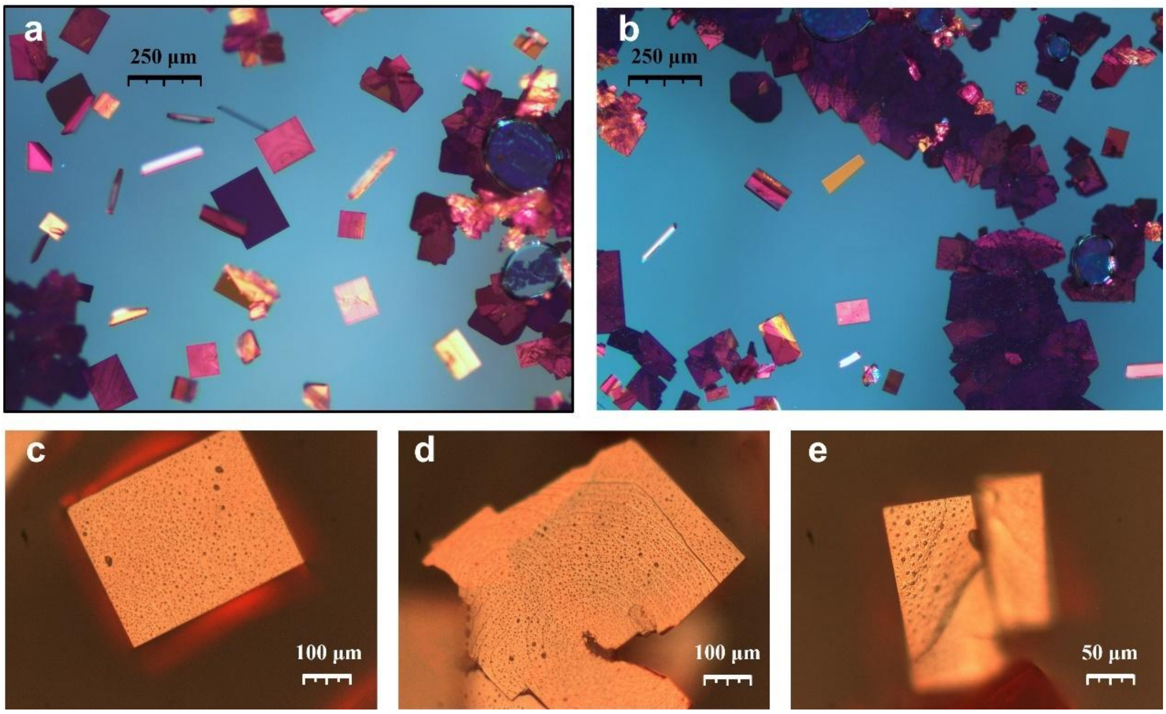

Figure S.5: Microscope images of perovskite $n = 3$ single crystals grown between glasses. The crystals have rectangular shape with $100 - 250\ \mu m$ lateral size and 1 to $10\ \mu m$ width and are positioned with a random angle on the substrate. In (**c**)-(**d**), the stacking layers can be distinguished on their surface, together with solvent residue.

Microscope optical images of the perovskite crystals grown between glasses and DBRs can be seen in **Figure S.6**. The crystals have acquired a variety of planar dimensions, ranging from some μm up to a half mm. The larger are aggregates of combined single crystals similar to that of freely grown crystals of **Figure S.2**. The smaller single crystals are usually positioned at the edges or inside the residues of solvent in the pressed cavity. There, they grow until they reach their maximum width, limited by the cavity size, or until there is no further perovskite material at their proximity. In the latter case, they have widths smaller than that of the cavity (around $1 - 5\ \mu m$), they float inside the solvent and some of them end up placed on the lower DBR or on other crystals with random orientation and oblique angle. Their iridescence and variable coloration indicate that their width is varied along their surface, with a different number of stacking layers at every point, such as in the case of freely grown crystals of **Figure S.3**. In **Figure S.7**, single crystals grown inside a DBR cavity are also presented, after the removal of the top DBR. Their uniformity, small width of about $3.5\ \mu m$ and random orientation is visible.

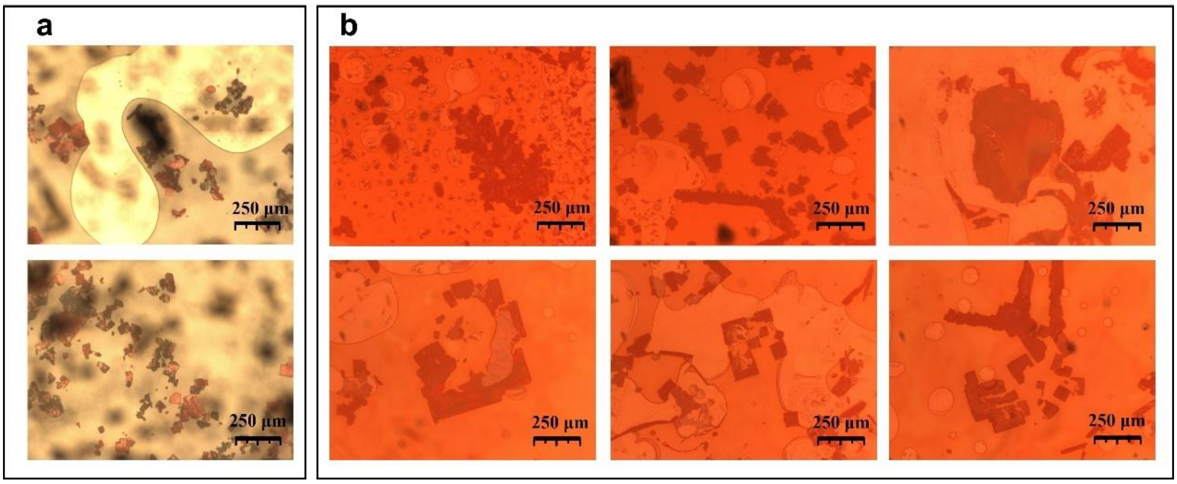

Figure S.6: Optical microscope images of perovskite crystals grown inside magnet pressed cavities. The crystals are confined by microscope glasses (**a**) or by Ta_2O_5/SiO_2 DBRs (**b**). Large aggregates can be seen formed, with lateral dimensions of up to $500\ \mu m$, as well as single crystals with sizes of $5-100\ \mu m$. The width of the crystals ranges from 1 to $5\ \mu m$ with the maximum limit being the width of the cavity. Single crystals also have a variation of width along their surface, evident by their iridescence and multiple coloration. They float inside the solvent residues until they are positioned with random angles on top of other crystals or the lower DBR.

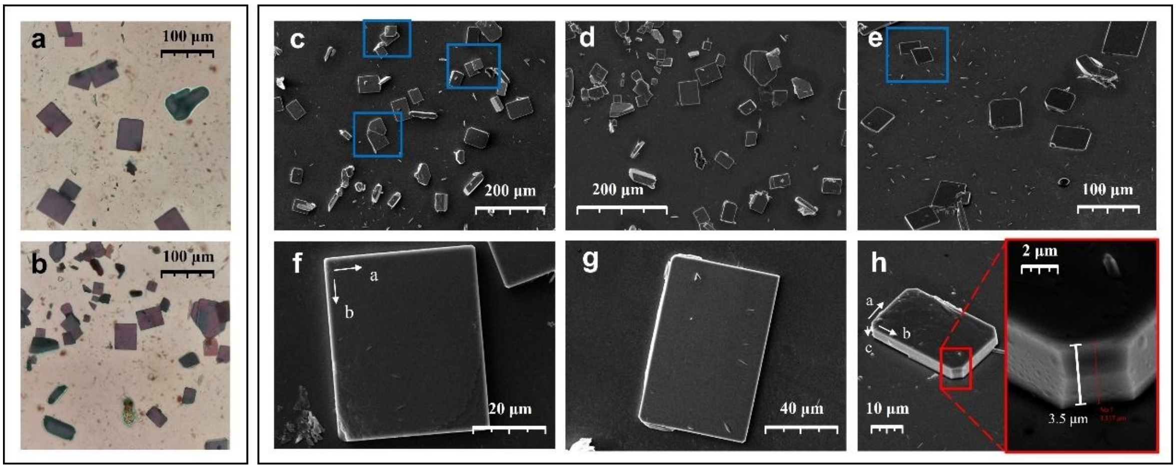

Figure S.7: Optical microscope and SEM images of single perovskite crystals formed inside a cavity between two DBRs after the removal of the top one. (**a, b**) Optical microscope images. (**c-e**) SEM images of multiple crystals with (**e**) taken at an angle. The blue boxes indicate the regions where crystals have been placed on top of each other resulting in a tilted position with respect to the DBR surface. (**f-h**) SEM images of single crystals with (**h**) taken at an angle. The quality of the crystals is evident by their tetragonal shape and their width is approximately $3.5\ \mu m$.

S.2.C Emission and reflectivity of crystals

Emission of various n's

In **Figure S.8**, the room temperature micro-photoluminescence (μ-PL) of pure perovskite crystals with different layer number n, grown in drop-casted solutions on Ta_2O_5/SiO_2 DBRs is presented. With the increase of n, the excitonic emission is shifted to higher wavelengths with $n = 1$ emitting at around 523 nm and $n = 6$ at 727 nm. That behavior corresponds to the widening of the quantum wells created by the organic layers and the subsequent red-shifting of the emission signal. The increase with n of fitted FWHM PL linewidth is also observed, starting at around 15 nm for $n = 1$ and reaching 50 nm for $n = 6$. In panel (**c**), the emission of $n = 3$ crystals that have been inserted into the microcavities is shown. They present excitonic resonance $X_{n=3}$ at 618 nm and a spectral FWHM linewidth of 23 nm.

The quality of the excitonic emission, with a single PL peak for every perovskite compound, depends on the purity of the initial solution. The presence of impurities or non-ideal fabrication conditions can lead to a proportion of the grown crystal having a layer number n not initially intended, resulting in multiple peaks in the PL spectra. This phenomenon cannot be completely eliminated but can be alleviated by a precise and careful synthesis process. Due to the slight presence of such impurities, all of the compounds show a small inhomogeneous broadening at higher wavelengths. Overall, the purity and condition of most of the crystals is almost perfect as confirmed by the XRD measurements shown in **Figure S.9**.

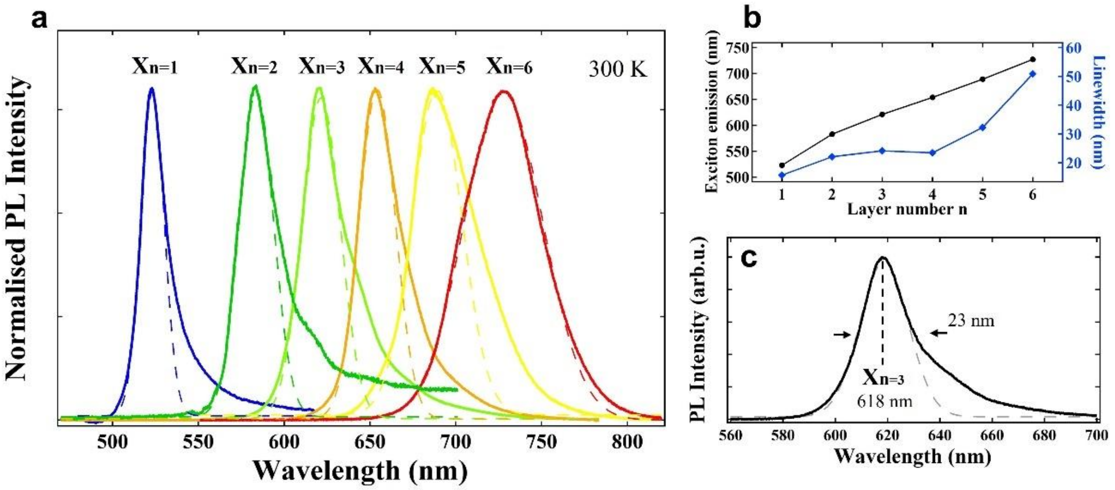

Figure S.8: μ-PL spectra of perovskite crystal compounds $n = 1,2,3,4,5,6$ at room temperature. (**a**) Excitonic emission X of every crystal: $X_{n=1} = 523\,nm$, $X_{n=2} = 583\,nm$, $X_{n=3} = 618\,nm$, $X_{n=4} = 654\,nm$, $X_{n=5} = 689\,nm$, $X_{n=6} = 727\,nm$ (**b**) Emission wavelength and linewidth for every n, having an almost linear dependence. (**c**) Spectral characteristics of the $n = 3$ compound, acting as the active medium in our polaritonic microcavities.

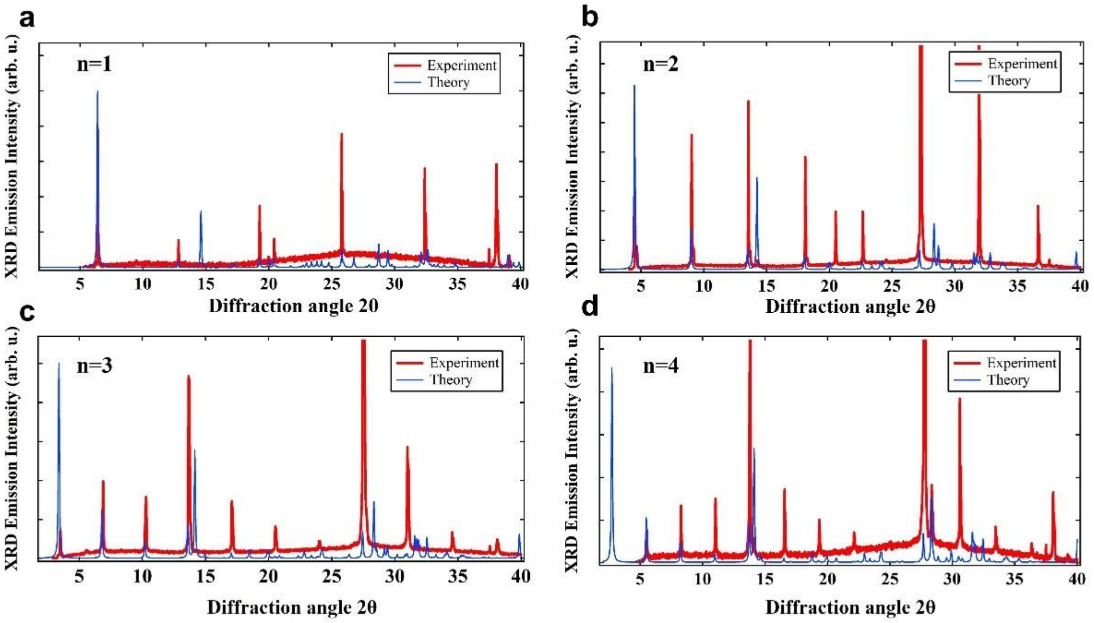

Figure S.9: XRD measurements of perovskite crystals of $n = 1,2,3,4$. The experimental measurements agree well with the theoretical diffraction angles data (taken from reference [46]) with the first peaks in each compound, for angles $2\theta < 10°$ indicating the number of inorganic layers. This proves that the crystals grown are sufficiently pure with high quality.

Edge effect

Even though the fabricated crystals are of high quality, external environmental conditions can have an effect on their structure and optical properties. When subject to mechanical pressure, high temperatures or humidity, cracks can form on their surface, their color changes to yellow, while a new peak appears in their spectra, close to the emission of bulk crystals. Also in some cases, the degradation is so large that the proper excitonic peak can disappear completely. This is a phenomenon previously observed in literature, referred to as the *edge effect* (EE) [54]. **Figure S.10** shows the spectral and morphological changes to a perovskite crystal with a combination of $n = 3$ and $n = 4$ after its heating to about $420\ K$. The sample is impure, with two main PL peaks corresponding to the two $n's$, but after the heating, an additional wider peak appears at around $750\ nm$ due to the edge effect. Many cracks also appear at the surface of the crystal creating many boundaries that edge states can populate and where the edge effect emission can originate from. The same behavior can be observed with the exposure of the samples to humid air after some days, or to moderate pressures. The spectral peak of the edge effect and its linewidth are not always the same, but range from $720\ nm$ to $780\ nm$, close to the 3D crystal limit, and being at least $55\ nm$, respectively.

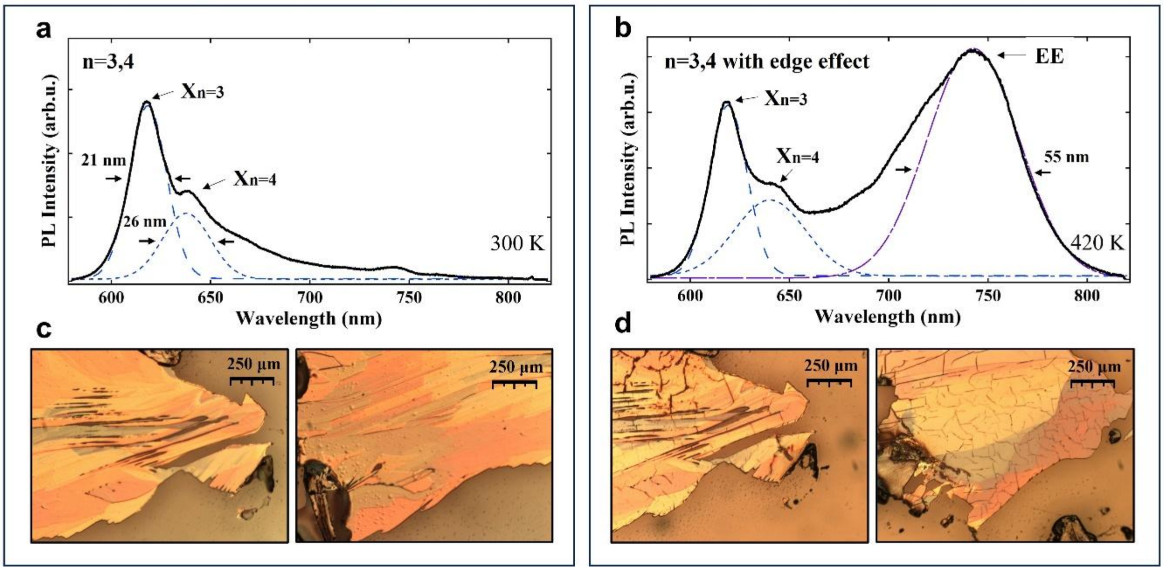

Figure S.10: Edge effect emission. (**a**) Room temperature PL for an impure perovskite crystal with $n = 3, 4$. Two main peaks $X_{n=3}$ and $X_{n=4}$ are visible corresponding the excitonic emission for the respected layer numbers, with linewidths less than $30\ nm$. (**b**) Room temperature PL after heating of the sample to $420\ K$. The appearance of the edge effect peak (EE) is visible with a spectral emission close to that of 3D perovskites and a FWHM larger than $50\ nm$. (**c**) Microscope images of perovskite crystals grown between glasses, before heating. (**d**) The same crystals after heating, with cracks appearing on their surfaces.

Reflectivity and substrates comparison

Perovskite crystals are grown by drop-casting either on microscope quartz glasses, silicon wafers or Ta_2O_5/SiO_2 DBRs, with the top layer being Ta_2O_5. Although the main characteristics of optical measurements are the same in all of these substrates, when the crystals are created on top of silicon wafers, their emission and reflectivity exhibit some interesting oscillation effects, shown in **Figure S.11**. In PL measurements, the oscillations appear at the spectral region between $630\ nm$ and $680\ nm$, close to the excitonic peak at $618\ nm$. This trend is repeated in reflectivity measurements, where they start forming at wavelengths higher than the reflectivity excitonic feature at $605\ nm$. It is worth mentioning that the overall reflectivity is much lower for smaller wavelengths, suggesting a higher light absorption in that range.

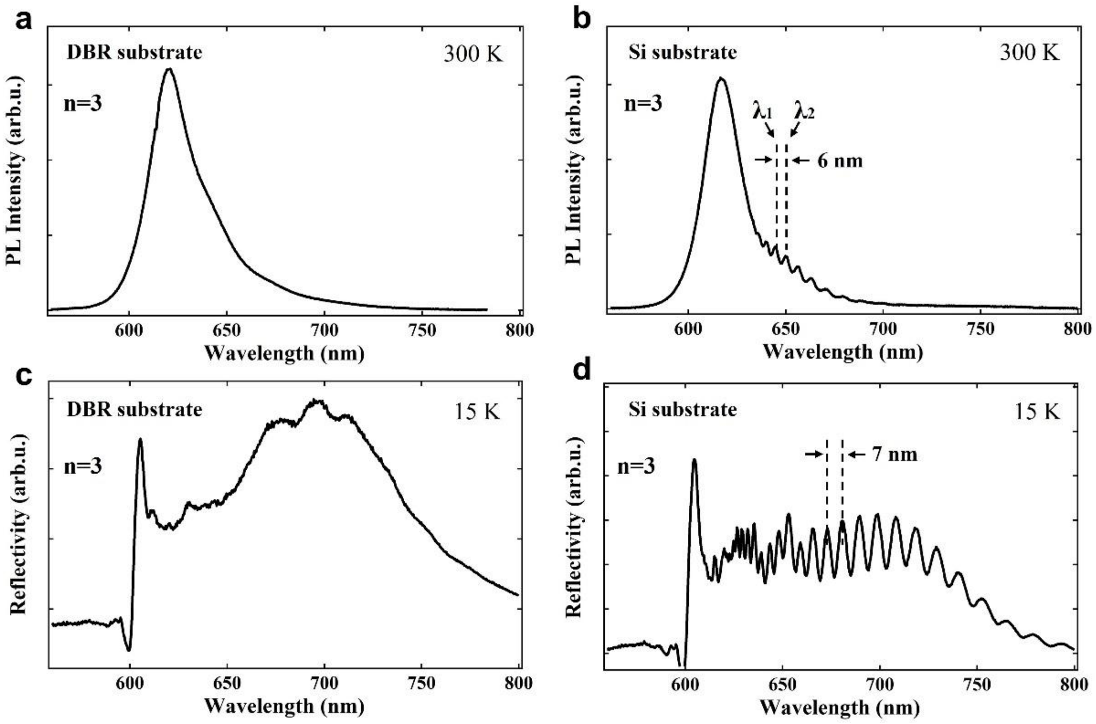

Figure S.11: Optical spectra comparison between perovskite crystals of $n = 3$ on Ta_2O_5/SiO_2 DBRs and Si wafers. (**a, b**) µ-PL spectra at room temperature for the two substrates with oscillations appearing for the Si case with period 6 nm, indicating an air cavity of about 30 μm length. (**c, d**) Reflectivity measurements at 15 K for the same samples. Oscillations are again present for wavelengths above the excitonic feature at 605 nm.

These effects are indications of the existence of a cavity, probably because of trapped air or hydrogen iodide (HI) solvent (with refractive index of 1.46) between the perovskite and the substrates. The length of this cavity can be estimated considering adjacent modes λ_1, λ_2 at around 650 nm with spectral distance $\Delta\lambda = \lambda_1 - \lambda_2$ of 6 nm and zero incident angle.

$$\begin{cases} \frac{2\pi}{\lambda_1} n_c L_c = m\pi \\ \frac{2\pi}{\lambda_2} n_c L_c = (m+1)\pi \end{cases} \rightarrow L_c = \frac{\lambda_1 \lambda_2}{2 n_c \Delta\lambda}$$

The result is that a space, filled with air or HI, exists between the crystals and the substrates, behaving as a cavity of around 30 μm and 20 μm width respectively, and generating the oscillation patterns. The oscillations are more intense when there is a lower refractive index mismatch of the materials acting as mirrors and when they have larger lengths, before absorption becomes a limiting factor. In our case, the first layer of the DBRs is Ta_2O_5 with refractive index of 2.1 at 650 nm. Si wafers always have a layer of SiO_2 on their surface with refractive index 1.45 at 650 nm. Perovskite crystals close to exciton resonance exhibit a refractive index of 1.11 for the stacking axis, as shown in **Section S.2.G**. Because of its

birefringence, the refractive index can vary up to 1.92 depending on the direction inside the crystal. As mentioned in the previous section, crystals are grown with random orientation and light incident on the surface of the perovskite sample can experience a crystal refractive index close to that of SiO_2 at some point. The similar refractive indices can result in strong oscillation modes from the air cavity when the substrate is Si. Oscillations still exist with DBR substrate, but they are weaker in intensity and the modes are further apart to be distinguishable from the perovskite emission signal and reflectivity.

S.2.D Temperature dependence

Temperature dependent measurements, with μ-PL presented in **Figure S.12**, are performed on the perovskite crystals of $n = 3$, by inserting them into the cryostat and lowering their temperature down to cryogenic conditions. As shown, the excitonic emission remains almost unchanged down to 150 K, where the peak is abruptly shifted by almost 20 nm to lower wavelengths. For lower temperatures, the emission remains stable until around 60 K when it starts to continuously redshift until it reaches 611 nm at 12 K. The peak FWHM has a linear dependence on temperature with its linewidth steadily narrowing as the temperature drops. In **Figure S.13**, a comparison of the excitonic emission for room temperature and 12 K is shown. At low temperature, the linewidth becomes 4 nm and the peak is blue-shifted by 7 nm from ambient conditions.

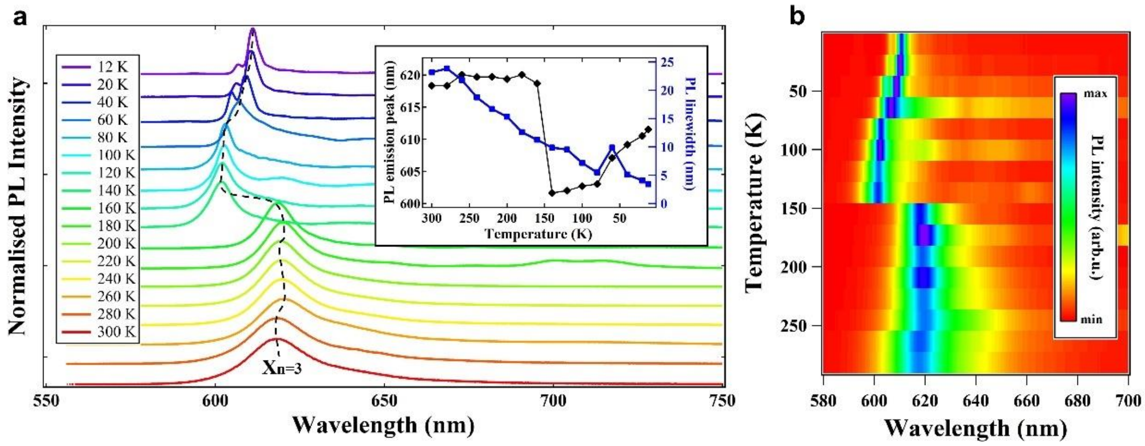

Figure S.12: Temperature dependent μ-PL of perovskite crystals with $n = 3$. (**a**) As the temperature continuously drops, the excitonic emission peak does not change spectrally until the phase change at 150 K where it blueshifts to 601 nm. As the temperature continues to decrease, the peak emission redshifts to 611 nm at 12 K, with a second phase change at 60 K. The linewidth steadily narrows until it reaches 4 nm at 12 K (**b**) The same as in (**a**) depicted in a contour plot, where changes in peak emission and linewidth are clearly shown.

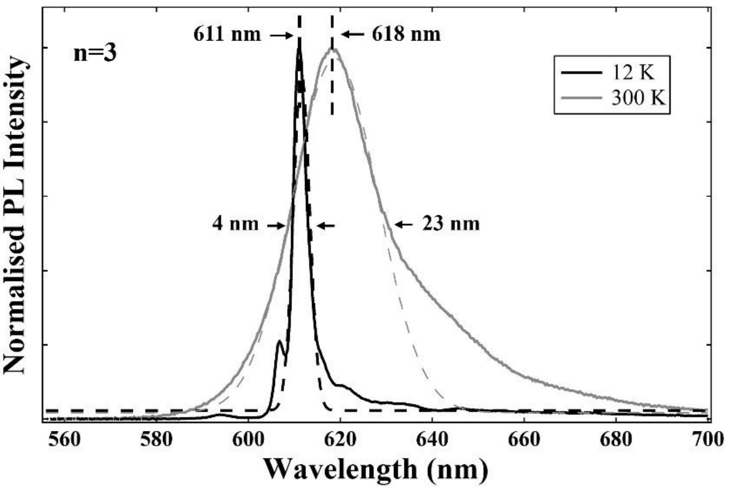

Figure S.13: μ-PL emission of $n = 3$ perovskite crystals at room temperature and $12\ K$. Excitonic resonance at $300\ K$ is centered at $618\ nm$, while at $12\ K$, it is shifted to $611\ nm$. The linewidth of the peak at cryogenic temperatures is $4\ nm$, at least five times narrower than that of room temperature.

The sudden change of optical spectra with the tuning of temperature is a characteristic effect of layered perovskites. At specific temperatures the crystal and band structure of these materials is reordered and a phase transition occurs [49, 51-52, 89-91]. The excitonic emission follows this change and is shifted to different wavelengths. In some cases, two structural phases can exist at the transition temperature resulting in two emission peaks. The exact point at which the phase transitions appear depends on the inorganic layer number n and the organic barrier, with $(BA)_2(MA)_2Pb_3I_{10}$ perovskites being known to experience two main phase changes at around $150\ K$ and $60\ K$, as our measurements depict. In **Figure S.14**, temperature dependent reflectivity of the same crystal shows another example of the main phase transition at $150\ K$, with the excitonic feature having a similar transition behavior to that of μ-PL measurements, as well as a notable difference in width between low and high temperatures.

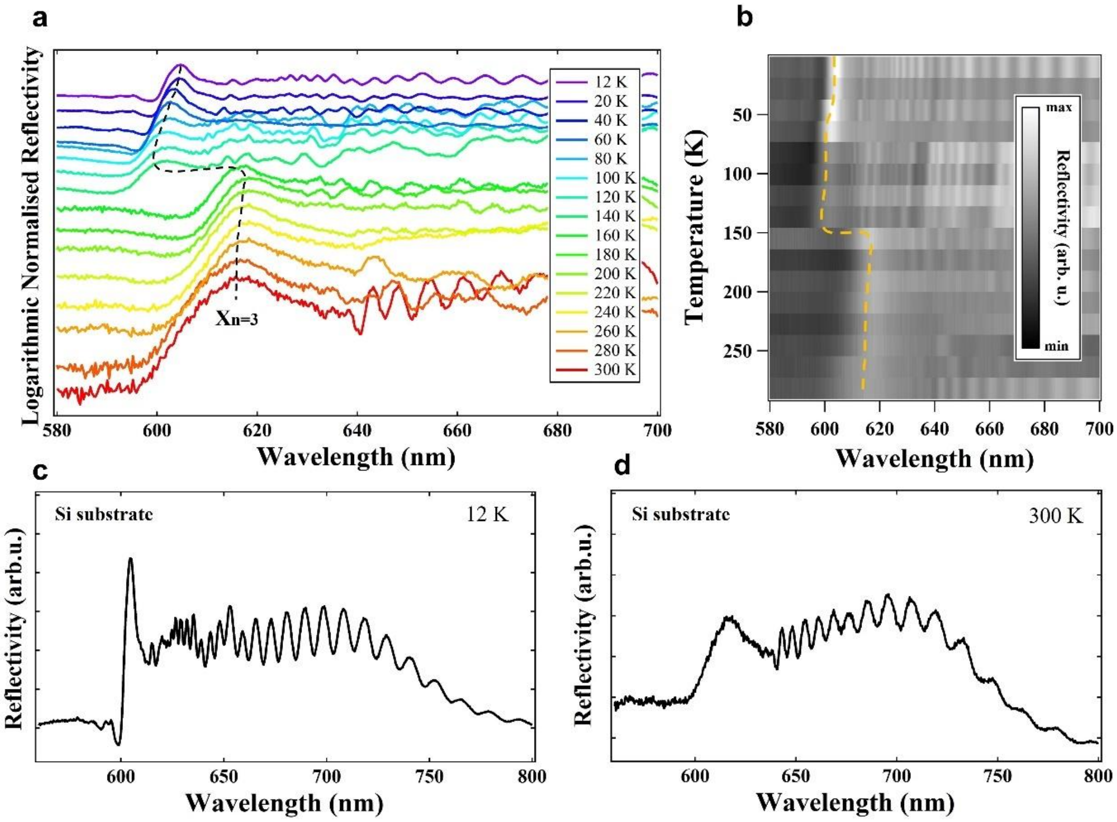

Figure S.14: Temperature dependent reflectivity measurements of perovskite crystals with $n = 3$. (**a**) The exciton feature changes spectral position similarly to that depicted in μ-PL measurements with a phase transition occurring at $150\,K$ (**b**) Contour plot of the same measurements with the excitonic shifts highlighted. (**c, d**) Comparison of low and room temperature reflectivities on Si substrate with oscillation effects and widening of the excitonic feature at $12\,K$.

S.2.E Power dependence

Perovskite crystals with $n = 3$ are also subjected to excitation power dependent μ-PL measurements in order to evaluate their endurance to increased laser fluence. In **Figure S.15**, the power dependent measurements are presented, firstly by increasing the pump fluence (power) from $9\,\mu J\ cm^{-2}$ $(25\,\mu W)$, up to $376\,\mu J\ cm^{-2}$ $(1\,mW)$. Then the experiment is repeated with descending power until the initial value. The beam fluence is tuned by the gradient ND filter of the optical setup.

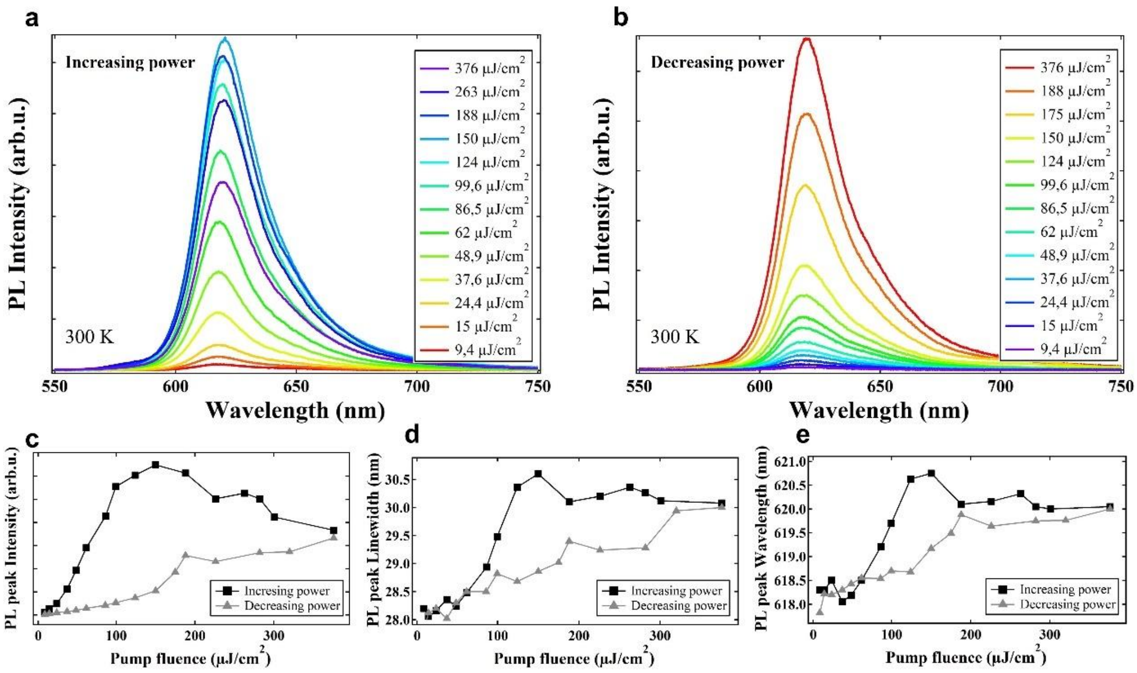

Figure S15: Excitation power dependent µ-PL measurements of $n = 3$ perovskite crystals at $300\ K$. (**a**), (**b**) Measurements with increasing and decreasing excitation fluence respectively. The PL peak intensity increases linearly until about $100\ \mu J\ cm^{-2}$ and maximizes at $150\ \mu J\ cm^{-2}$ (**c**). With further rise of fluence, the PL peak intensity drops to almost half of its maximum value. By subsequently decreasing the fluence, the intensity is linearly and rapidly reduced without attaining again the maximum value. The PL peak linewidth (**d**) and wavelength (**e**), although stable at low fluences, progressively increase with power until they are changed by $2.5\ nm$ and $3\ nm$ respectively at $150\ \mu J\ cm^{-2}$. After that, they decrease slightly and eventually return to their initial values when power is decreased.

Initially, the rise in intensity is almost linear as the fluence increases. However, at around $100\ \mu J\ cm^{-2}$ the increase starts to slow down and eventually stops at $150\ \mu J\ cm^{-2}$. With further power increase, the emission continuously decreases up until $376\ \mu J\ cm^{-2}$ to almost half the maximum intensity. By reversing the change in fluence, the peak intensity rapidly decreases almost linearly with a greater rate than previously. This behavior is mirrored by the linewidth of the emission peak but also by its wavelength. The FWHM changes from $28\ nm$ to $30.5\ nm$ at $150\ \mu J\ cm^{-2}$ and then stabilizes at around $30\ nm$ for higher fluence. With descending power, it decreases almost linearly to the initial value. The emission peak is redshifted by $3\ nm$ at $150\ \mu J\ cm^{-2}$ and then follows the trend of the other quantities, with the return to $618\ nm$ at $9\ \mu J\ cm^{-2}$.

The change of spectral properties, although slight, indicates that a structural change presumably takes place in the crystals when the fluence exceeds $100\ \mu J\ cm^{-2}$, although additional evidence is required to confirm this hypothesis. The fact that layered perovskites are composed of both organic and inorganic parts makes them very sensitive to the rapid changes of temperature when subject to intense beam fluences. Because there is no efficient dissipation mechanism, the excess energy can result in reordering of organic large cations to different states, with a phase change taking place. These phase transitions affect the optical properties of the material and its emission texture. In some cases, the change is irreversible

with the excitonic emission completely disappearing and the emergence of edge effect peak. At this point the crystal structure has degraded and cannot be used as a medium for strong coupling in microcavities. Due to that, in our main experiments with polaritonic microcavities, we have used pump fluences below $50\ \mu J\ cm^{-2}$ to avoid destroying our samples and obtain a stable optical response.

S.2.F Time resolved measurements

The temporal evolution of a perovskite $n = 3$ crystal PL at $618\ nm$ is measured as well as that of edge effect emission at around $750\ nm$ at a slight degraded sample, as depicted in **Figure S.16**. The modeling of the temporal profiles results in a double exponential time dependence with two characteristic lifetimes for both emissions.

The excitonic resonance presents a rise time of about $74\ ps$, a long lifetime of around $400\ ps$ and a short of around $110\ ps$. The transitions and temporal evolution of exciton populations are defined by these lifetimes and should indicate the two mechanisms of recombination, non-radiative and radiative. Although the non-radiative path does not emit any light, it affects the overall exciton initial population and thus its effects can be revealed by the dynamics of the radiative emission.

The edge effect emission has similar rise time of $81\ ps$ but a much larger long lifetime of $1230\ ps$. The short lifetime is almost the same at that of the excitonic emission. The large divergence between the lifetimes is a further indication that the edge effect emission is not excitonic and has a different nature and origin from that of usual transitions.

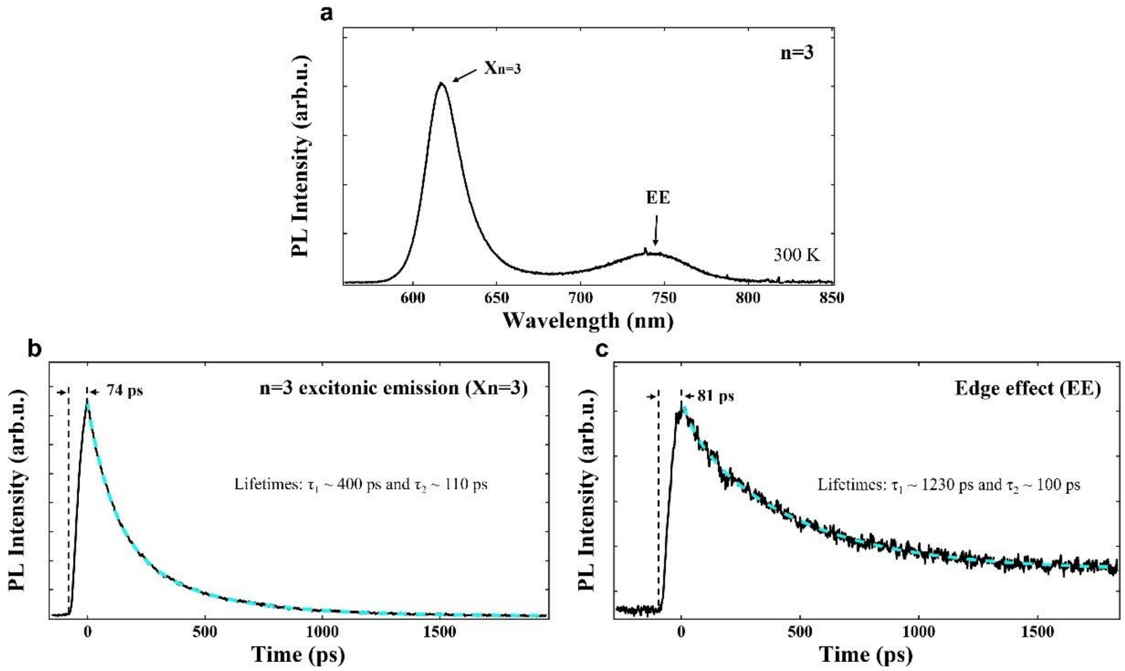

Figure S.16: Time resolved μ-PL measurements of $n = 3$ perovskite crystals at 300 K. (**a**) Spectral profile of the excitonic ($X_{n=3}$) at $618\ nm$ and edge effect emission (EE) at around $750\ nm$. (**b**) Temporal evolution of $X_{n=3}$ with a rise time of $74\ ps$ and a double exponential lifetime of $400\ ps$

and $110\ ps$. (**c**) The same as in (**b**) in the presence of the EE, with a $81\ ps$ rise time, a long lifetime of $1230\ ps$ and a short of $100\ ps$.

S.2.G Birefringence

$(BA)_2(MA)_2Pb_3I_{10}$ perovskite crystals can be generally approximated as uniaxial birefringent materials, with the refractive indexes n_x, n_y on the plane of $\boldsymbol{a}$, $\boldsymbol{b}$ axes having a similar value n_o (ordinary), while that on the layer stacking axis $\boldsymbol{c}$ being much different, $n_z = n_e$ (extraordinary). The refractive index is related to the dielectric function of the layered perovskites which, for the general case, is expressed by the tensor:

$$\boldsymbol{\varepsilon_p}(\omega) = \begin{pmatrix} \varepsilon_{xp}(\omega) & 0 & 0 \\ 0 & \varepsilon_{yp}(\omega) & 0 \\ 0 & 0 & \varepsilon_{zp}(\omega) \end{pmatrix}$$

with $\varepsilon_{xp} = \varepsilon_0 n_x^2$, $\varepsilon_{yp} = \varepsilon_0 n_y^2$, $\varepsilon_{zp} = \varepsilon_0 n_z^2$ the elements of the crystal dielectric tensor expressed on its own coordinate system (x_p, y_p, z_p) with axes parallel to the principal crystallographic directions $(\vec{a}, \vec{b}, \vec{c}\,)$. Using crystallographic data (extracted from reference [46]) for the exact structure of the crystals and employing density functional perturbation theory, we calculate the dielectric tensor as:

$$\boldsymbol{\varepsilon_p} = \begin{pmatrix} 4.643 & 0 & 0 \\ 0 & 4.643 & 0 \\ 0 & 0 & 3.702 \end{pmatrix}$$

For semiconductors and metals, the dielectric function, has to be modified to account for the response to the AC electromagnetic field of light, according to the Drude-Lorentz model. Away from excitonic resonances, and without including losses it can be written as:

$$\varepsilon(\omega) = \varepsilon_0 - \frac{\varepsilon_0 \omega_p^2}{\omega^2} = \varepsilon_0 \left(1 - \frac{\omega_p^2}{\omega^2}\right)$$

with $\omega_p^2 = \frac{Ne^2}{M\varepsilon_0}$ being the plasma frequency. By setting $\omega_p^2 = 1.508\ eV$, the extraordinary dielectric function on the z_p axis, $\varepsilon_{zp}/\varepsilon_0$, is calculated to gradually reach 1.245 at $650\ nm$ with the extraordinary refractive index being 1.116. Closer to the excitonic emission, at $620\ nm$, its influence is expected to further modify the refractive index by increasing it to a larger value.

From the microscope images, it is observed that the crystals may grow at a tilted position on top of the substrates, defined by the Euler angles φ, θ, ψ, as depicted in **Figure S.17**. In that case, the lab coordinate system (x, y, z) is different from that of the crystal and the dielectric tensor, is modified into:

$$\boldsymbol{\varepsilon}(\omega) = \begin{pmatrix} \varepsilon_{xx}(\omega) & \varepsilon_{xy}(\omega) & \varepsilon_{xz}(\omega) \\ \varepsilon_{yx}(\omega) & \varepsilon_{yy}(\omega) & \varepsilon_{yx}(\omega) \\ \varepsilon_{zx}(\omega) & \varepsilon_{zy}(\omega) & \varepsilon_{zz}(\omega) \end{pmatrix}$$

The transformation between the two tensors is described in **Section S.9** where the effective system Hamiltonian is derived, taking into account the effect of the crystal birefringence on the different polarizations of light, inside a microcavity.

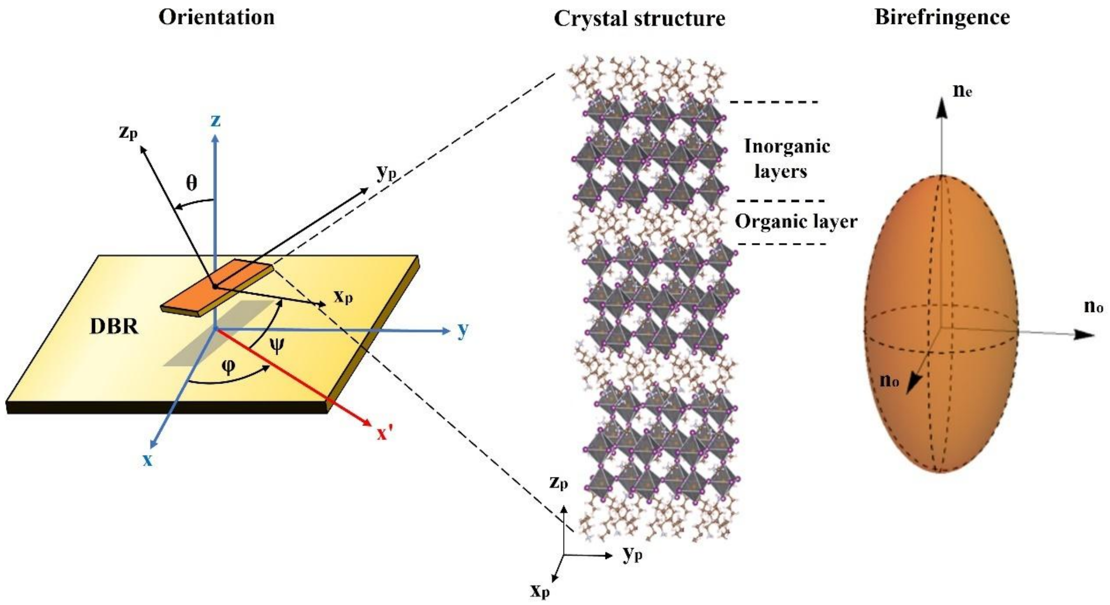

Figure S.17: Schematic of the perovskite orientation and birefringence when grown between DBRs with the solution method. The crystals attain a random orientation with Euler angles φ, θ, ψ with respect to the plane of the cavity. Their coordinate system (x_p, y_p, z_p) is rotated relative to that of the cavity (x, y, z) and their axes are parallel to the main crystallographic axes $\boldsymbol{a}$, $\boldsymbol{b}$ and $\boldsymbol{c}$. Because of the birefringence and their uniaxial nature, the crystals exhibit an ordinary refractive index n_o along x_p and y_p axes and an extraordinary refractive index n_e along the z_p axis.

S.3 Perovskite crystal synthesis and microcavity fabrication

S.3.A Synthesis of perovskite solution and crystals

The two-dimensional Ruddlesden–Popper perovskite crystal thin sheets are synthesized using an interfacial growth method, based on the procedure followed in [92], with the precursor species, concentrations, and ratios carefully optimized and the crystallization temperature carefully controlled to promote layer-by-layer growth, avoid dislocation formation, maximize lateral growth and maintain phase purity.

For the $(\mathrm{BA})_2(\mathrm{MA})_{n-1}\mathrm{Pb}_n\mathrm{I}_{3n+1}$ series and specifically the $(\mathrm{BA})_2(\mathrm{MA})_2\mathrm{Pb}_3\mathrm{I}_{10}$ $(n = 3)$ compound (with BA and MA abbreviations for n-butylammonium and methylammonium, respectively), the synthesis is described below:

Lead iodide (PbI_2) $(0.59M)$ and methylammonium chloride ($\mathrm{CH_3NH_3Cl}$) $(0.40M)$ precursors are dissolved in a concentrated aqueous solution of hydrogen iodide ($\mathrm{HI}, 57\% \frac{w}{w}$ in $\mathrm{H_2O}$) and hypophosphorous acid ($\mathrm{H_3PO_2}, 50\% \frac{w}{w}$ in $\mathrm{H_2O}$) mixture $(10{:}1\ vol/vol)$ and then heated at $130°C$ in a closed stirred glass vial until a clear, yellow solution is obtained. This step follows the chemical reactions:

$$n\mathrm{PbO} \xrightarrow{\mathrm{HI_{(aq)}}} n\mathrm{Pb}^{2+} + 2n\mathrm{I}^- + n\mathrm{H_2O_{(l)}}$$

$$(n-1)\mathrm{CH_3NH_3Cl} \xrightarrow{\mathrm{HI_{(aq)}}} (n-1)\mathrm{CH_3NH_3}^+ + (n-1)\mathrm{Cl}^-$$

In a separate beaker, n-butylamine ($\text{n-}\mathrm{CH_3(CH_2)_3NH_2}$) $(0.19M)$ is neutralized with HI $57\% \frac{w}{w}$ in $\mathrm{H_2O}$ $(2\ mL)$ according to the following reaction, resulting in a clear pale-yellow solution and then is added to the $\mathrm{PbI_2}$ solution slowly.

$$2(\mathrm{CH_3(CH_2)_3NH_2})_{(l)} \xrightarrow{\mathrm{HI_{(aq)}}} 2(\mathrm{CH_3(CH_2)_3NH_3})^+ + 2\mathrm{I}^-$$

The solution is then cooled down to $65\ °C$ and kept at that temperature in a closed vial in a sand bath as the stock solution. $2\ \mu L$ of this warm supernatant solution are collected and dispensed with a $10\mu L$ pipette onto a glass slide placed in an open ambient environment $(25\ °C)$. Nucleation and growth quickly initiate on the surface of the precursor solution droplet and perovskite thin sheets floating on the droplets are obtained within a few seconds up to $30\ s$.

$$2(\mathrm{CH_3(CH_2)_3NH_3})^+ + (n-1)\mathrm{CH_3NH_3}^+ + n\mathrm{Pb}^{2+} + (3n+1)\mathrm{I}^- \xrightarrow[-\Delta]{\mathrm{HI_{(aq)}}}$$

$$(\mathrm{CH_3(CH_2)_3NH_3})_2(\mathrm{CH_3NH_3})_{(n-1)}\mathrm{Pb}_n\mathrm{I}_{(3n+1)_{(s)}}$$

This growth process on the surface of the droplet on glass slides is observed directly under an optical microscope to monitor the progress. For each n, different and precise amounts of precursors are required for the crystal formation while, for $n = 1$, methylammonium chloride is omitted from the process. The grown perovskite crystals for $n = 1 - 6$ are presented in **Figure S.18**.

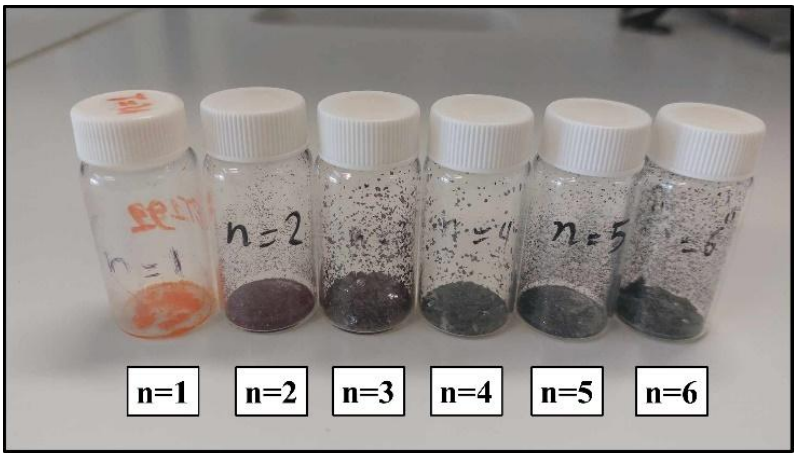

Figure S.18: Naturally grown two-dimensional perovskite single crystals of $(BA)_2(MA)_{n-1}Pb_nI_{3n+1}$. Different layer numbers n result in color variations of the crystals, especially for those with lower $n's$, while higher $n's$ appear black to the naked eye due to their absorption shifting to the near infrared.

The stoichiometric synthesis method can provide 2D perovskite single crystals with sufficient purity and single exciton emission peaks. However, the width and morphology of the crystals cannot be directly controlled, with the formation of many alternative organic and inorganic layers, similar to a multiple quantum well (MQW) structure, and a total width of several tens of micrometers. In the bulk, the crystals cannot be inserted into a microcavity without compromising either the integrity of the crystal and its emission, the quality of the cavity or both.

With the supersaturated solution method, the initial solution is purposely modified to be oversaturated. The perovskite solubility decreases with decreasing solution temperature and therefore tuning of temperature can control the formation and the dissolution of crystals. Keeping the solution at a specific critical temperature, very close to the nucleation point, and inserting it into a space-confining structure, enables the crystals that form when the solution is cooled, to take the shape of their surroundings. In that way, the width of the grown crystals depends on the space that confines them and because there is no mechanical pressure involved, their purity, quality and emission is unaffected. This method was used for the creation of strongly-coupled microcavities, where the confined environment is defined by DBRs.

S.3.B Microcavities Fabrication

Polariton formation strongly depends on the strong excitonic emission and the quality of the cavity. Although two-dimensional perovskite crystals show enhanced optical properties, the task of realizing a cavity with them as the active material with sufficient quality factor for strong coupling is not trivial. Perovskite crystals are very sensitive to environmental conditions and can degrade with increased temperature or pressure. They also tend to form large bulk aggregates with irregular shapes that cannot be used for the formation of a uniform and high-quality cavity.

For our studies, we have synthesized two kinds of microcavities, consisting of perovskite crystals sandwiched between DBRs. The first consists of 10 layers of Ta_2O_5/SiO_2 (starting with Ta_2O_5) on a quartz substrate, with an extra top layer of Ta_2O_5, and stop-band centered at $650\ nm$. The second consists of the same structure but on a sapphire substrate and stop-band centered at $700\ nm$. Initially the DBRs are thoroughly cleaned with acetone for the removal of any organic dirt. Then the bottom DBR is placed on an aluminum stand heated by a hot plate at around $80\ °C$. The perovskite solution is also heated and stirred at $100°C$ for about $5\ min$ until all the pre-crystals are dissolved inside the solvent. Then $5\ \mu L$ of the solution is quickly drop-casted on the hot DBR before the new crystals start to form. Lastly, the sample is covered with the top DBR, that is again heated at $80\ °C$, and another aluminum plate. The cavity is then placed between two permanent ring-shaped magnets that firmly hold together the DBRs with uniform force, as shown in **Figure S.19**, and is removed from the hot plate to cool down. After 2 days inside a glove box with a nitrogen atmosphere, several micrometers wide crystals are grown inside the microcavity. Then the magnets are carefully detached and the surface of the sample is cleaned from residual material/solvent using acetone.

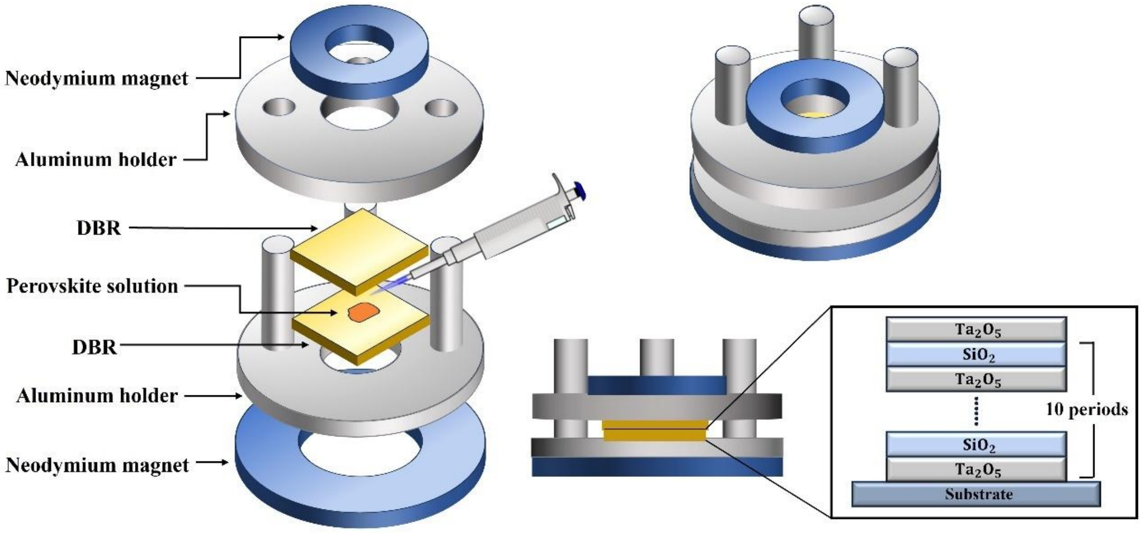

Figure S.19: Perovskite cavity fabrication process. The perovskite solution is drop-casted on a pre-heated DBR and quickly covered by another DBR at the same temperature. The DBRs are kept in place by two pieces of aluminum holders while a pair of neodymium magnets provide the necessary uniform force for the confinement of the thin solution layer between the DBRs. The setup is left to cool naturally and after two days, perovskite crystals are grown, with a width approaching that of the

cavity. The DBRs consist of 10 periods of Ta_2O_5/SiO_2 layers on quartz or sapphire substrate, with an extra Ta_2O_5 layer on top.

The aim of this type of fabrication is for the perovskite crystals to form only when their solution is confined in a predetermined and uniform space, determined by the magnets field, and to grow as little as possible vertically and as large as possible horizontally. Although not as tunable as other advanced techniques, the method proposed here does not require any complicated equipment, is fast, inexpensive, highly reproducible and produces crystals and cavities with very good quality that are able to manifest polaritonic strong coupling and spin orbit interactions with sufficient magnitude.

S.4 Microcavities properties

S.4.A Distributed Bragg Reflectors

The DBRs used for the fabrication of the cavities are of two types: one with stop band centered around $650\,nm$ and the other around $700\,nm$. The 10 periods of alternate $\mathrm{Ta_2O_5/SiO_2}$ layers ensure that the reflectivity of the mirrors would be close to unity at the center wavelength and would form a stop band with clear and sharp edges, called the first Bragg modes (BM), with all factors contributing to their ability to create cavities with high quality factors. This can be observed in **Figure S.20** with the simulation of the DBRs reflectivity being very close to the experimental measurements for both types.

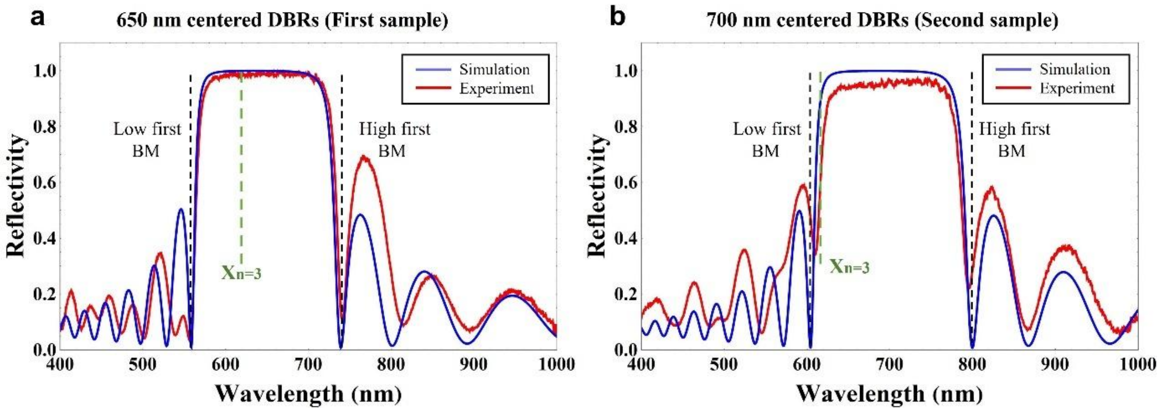

Figure S.20: Simulated (blue solid line) and experimental measurements (red solid line) at 0° incident angle and at room temperature of the Ta_2O_5/SiO_2 DBR reflectivities used in the fabrication of the perovskite cavity samples. The first set has a stop band centered at $650\,nm$ (**a**), while the second at $700\,nm$ (**b**). At the first sample, the perovskite excitonic emission $X_{n=3}$ is positioned close to the center of the stop band exhibiting a cavity reflectivity close to unity. At the second sample, $X_{n=3}$ is placed to a point adjacent to the first lower Bragg mode (BM) with slightly lower reflectivity.

Although large reflectivities are essential for the confinement of light inside the cavities and the formation of polaritons, they also hinder the collection of the excited material emission.

This fact, combined with the increased absorbance of perovskite crystals for wavelengths lower than $600\ nm$, affect the experimental visibility of excitonic effects when its resonance is set at the center of the stop band. This may be the reason why in the cavity fabricated with the DBR stop band center at $650\ nm$, adjacent to the excitonic emission at $620\ nm$, no polaritonic effects could be identified. On the contrary, the second set of DBRs, with stop band center at $700\ nm$ and first Bragg mode at $610\ nm$, showed strong polaritonic interaction, probably due to the slightly lower reflectivity at that wavelength.

S.4.B Optical cavity modes

The two perovskite cavities, fabricated with the sets of DBRs, are optically characterized using reflectivity and μ-PL measurements. Due to the nature of the fabrication process, every point on the samples has different properties, such as a varied width and effective refractive index. This results in cavity modes having different characteristics and dispersion behavior.

Reflectivity measurements

The comparison of reflectivities at zero incident angle of the bare DBRs and their corresponding cavity is shown in **Figure S.21**. The first Bragg modes for both samples have remained at almost the same spectral positions, with the resulting cavity stop bands spanning from $558\ nm$ to $748\ nm$ for the first sample, and from $610\ nm$ to $792\ nm$ for the second. Although the cavity modes for the first sample are not easily visible inside the stop band, the second sample exhibits multiple cavity modes, with clear reflectivity dips. From the linewidth of these dips, the quality factor of the cavity at that point can be deduced as $\mathrm{Q} \approx 870$.

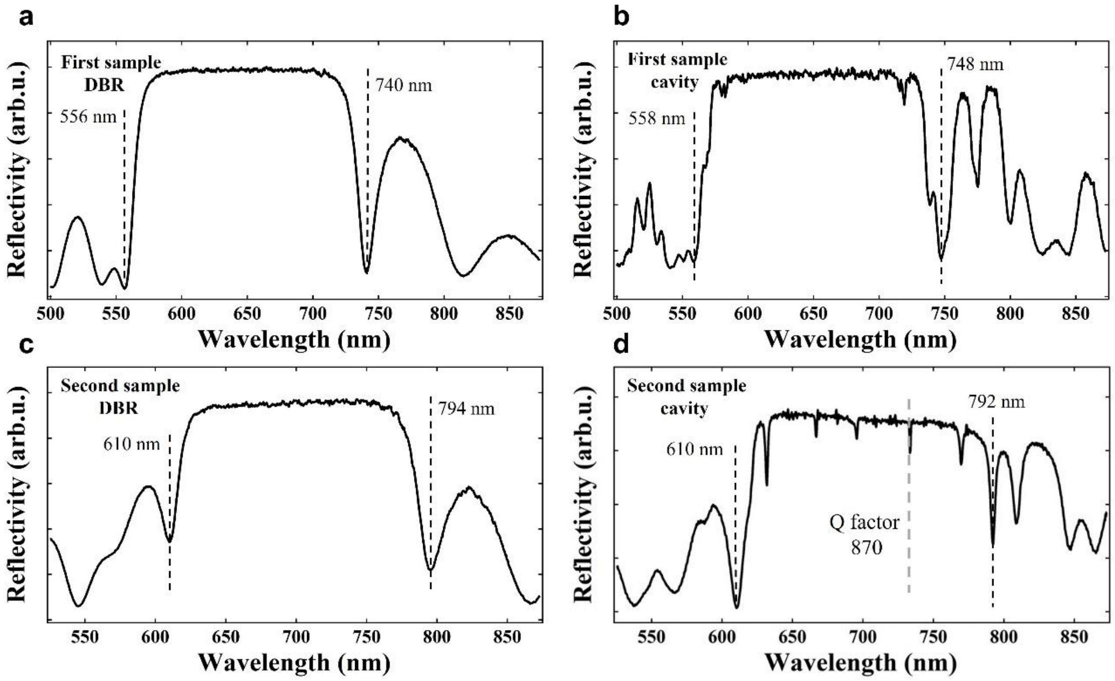

Figure S.21: Comparison of reflectivity measurements at 0° incident angle and at room temperature, of the two sets of Ta_2O_5/SiO_2 DBRs and their corresponding perovskite cavities. (**a**) The stop band of the first set of DBRs and its cavity (**b**), having a spectral width from $558\,nm$ to $748\,nm$. (**c**) Second sample DBR and microcavity (**d**) with a stop band spanning from $610\ nm$ to $792\ nm$. The cavity modes of the second sample are clearly observed as reflectivity dips with a quality factor of $Q = 870$.

Angle resolved photoluminescence

Using the configuration of **Section S.2.A**, we are able to acquire angle resolved photoluminescence from the two fabricated cavities at room temperature, that presents the dispersion characteristics of the cavity modes for each system. The measurements for the first sample are shown in **Figure S.22**, with a series of equally distanced parabolic curves corresponding to the confined cavity modes. We can observe that although the excitonic emission should be positioned at the center of the DBRs stopband, there is no single PL peak present at the perovskite emission wavelength for $X_{n=3}$ of around $620\ nm$. Instead, there exists a spectrally wide emission that strongly resembles that of the edge effect at higher wavelengths. This assumption is further supported by the fact that there is no observable anti-crossing or interaction with the cavity modes inside the stopband that would manifest the light-matter coupling and hence the existence of an exciton. Therefore, the emission is not excitonic and should be attributed to the edge effect, arising from the degeneration of the perovskite crystals, probably due to not ideal crystal growth conditions or the application of post-fabrication excess pressure by the DBRs. In any case, the absence of excitons in this sample allows the observation of pure photonic interactions as described in the main text.

The close proximity of the modes indicates that the cavity is relatively large. Using fitted curves to simulate the modes, the width of the cavity is estimated to be around $6 - 8\ \mu m$, depending on the exact measured spot, with an effective refractive index of about 1.5. Depending on the orientation of the crystal, the incident light experiences an effective

refractive index ranging from 2.1, the ordinary refractive index n_o, to the corrected value of the extraordinary refractive index n_e of 1.1. Due to the correction, the refractive index can also vary for different wavelengths.

TE-TM and XY splitting is observable for many positions on the sample, although their magnitude varies considerably for different crystals. At the point of **Figure S.22b**, the XY splitting is around 2 nm or 5 meV, while the TE-TM splitting is very small. On the contrary, in **Figure S.22c** both effects are more intense, with a large TE-TM splitting and a variable wavelength dependent XY splitting ranging from 3 meV to 46 meV. The combination of the splittings shift the modes into intersecting with each other multiple times, although no interaction effects seem to be pronounced at this particular point.

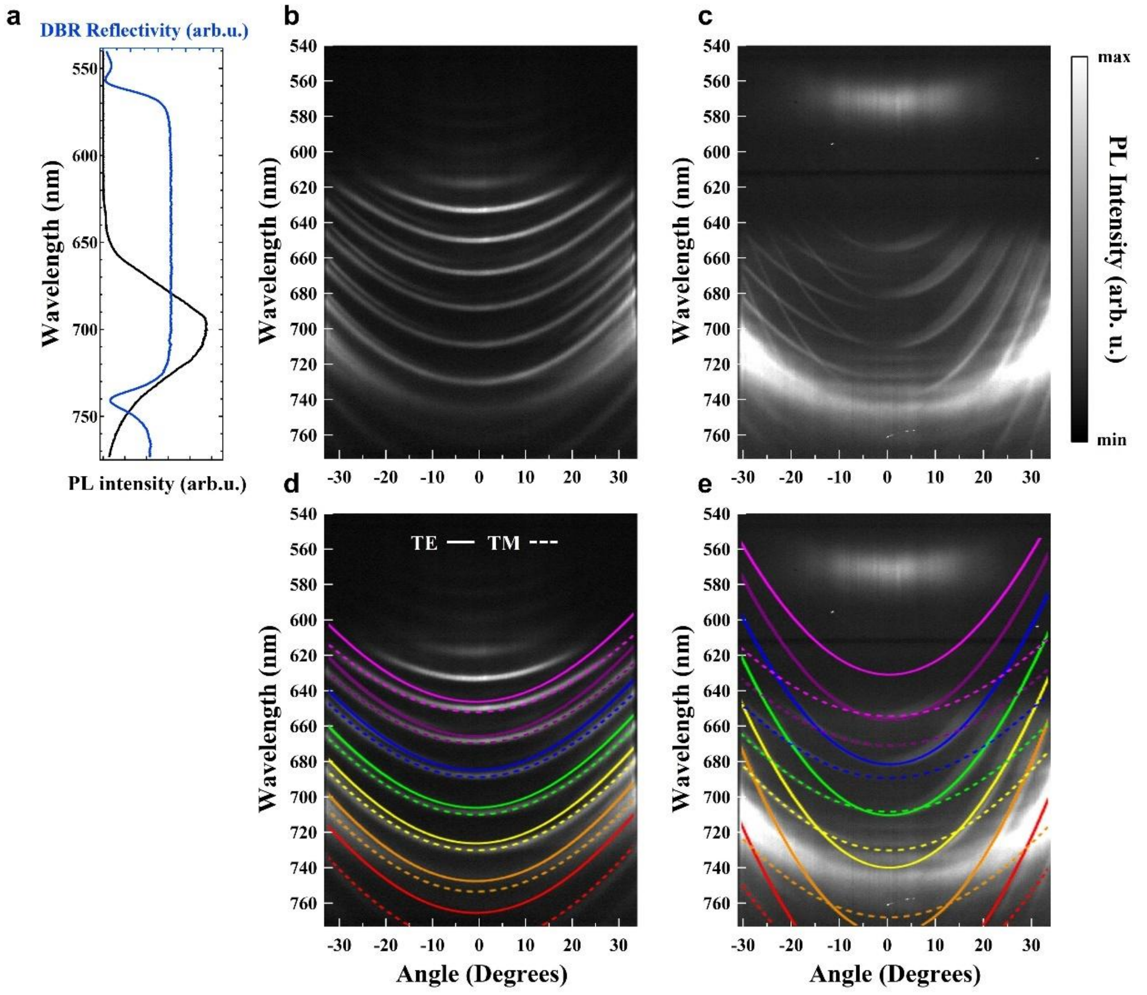

Figure S.22: Angle resolved micro-photoluminescence measurements at room temperature on different positions at the first perovskite cavity sample. The emission from this sample is attributed to the non-excitonic edge effect (**a**) DBR reflectivity and edge effect emission ranging from 650 nm to 750 nm. (**b, d**) Dispersion of multiple cavity modes for a point at the sample with mode spectral distance indicating a cavity length of about 6 μm and 1.5 effective refractive index. (**d**) Fitting of the dispersion curves and observation of a 5 meV XY splitting of TE (solid lines) and TM (dashed lines) polarizations. The modes with different quantum numbers are depicted with different colors. (**c, e**) Another sample point where TE-TM and XY splittings are very large. XY splitting is wavelength

dependent and can reach values up to 46 meV. (**e**) The modes are shifted and intersect with each other at multiple wavelengths without interacting.

Similarly to the first sample, we perform angle resolved PL measurements to the second sample at room temperature that are presented in **Figure S.23**. Here, the excitonic resonance emission is visible with an expected linewidth and spectral position close to the lower first Bragg mode of the DBRs. At the points of **Figure S.23**, the exciton does not interact with the photonic modes, enabling us to measure their pure dispersion curves.

The dispersion curves of **Figures S.23(b, e)** show modes with uniform curvature and distance indicating a cavity length of 4 μm and an effective refractive index of 1.7. In **Figures S.23(c, f)**, a similar set of modes can be observed but with those of higher quantum number exhibiting smaller curvature. This indicates that at those wavelengths the refractive index is higher, as one should expect in this spectral region where the exciton emission exists. Although there is no obvious strong coupling interaction, the exciton is still able to influence the dielectric response of the material. At both of these points the TE-TM and XY splittings have little magnitude and their effects are not pronounced.

At the point of **Figures S.23(d, g),** however, the situation is different. Here, we can observe large wavelength dependent XY splittings, reaching up to 34 meV and similarly large TE-TM splitting. Again, the excitonic resonance influences the effective refractive index for wavelengths in its proximity, but interestingly, only one polarization seems to be affected by it, with the dispersions of the other retaining their curvature.

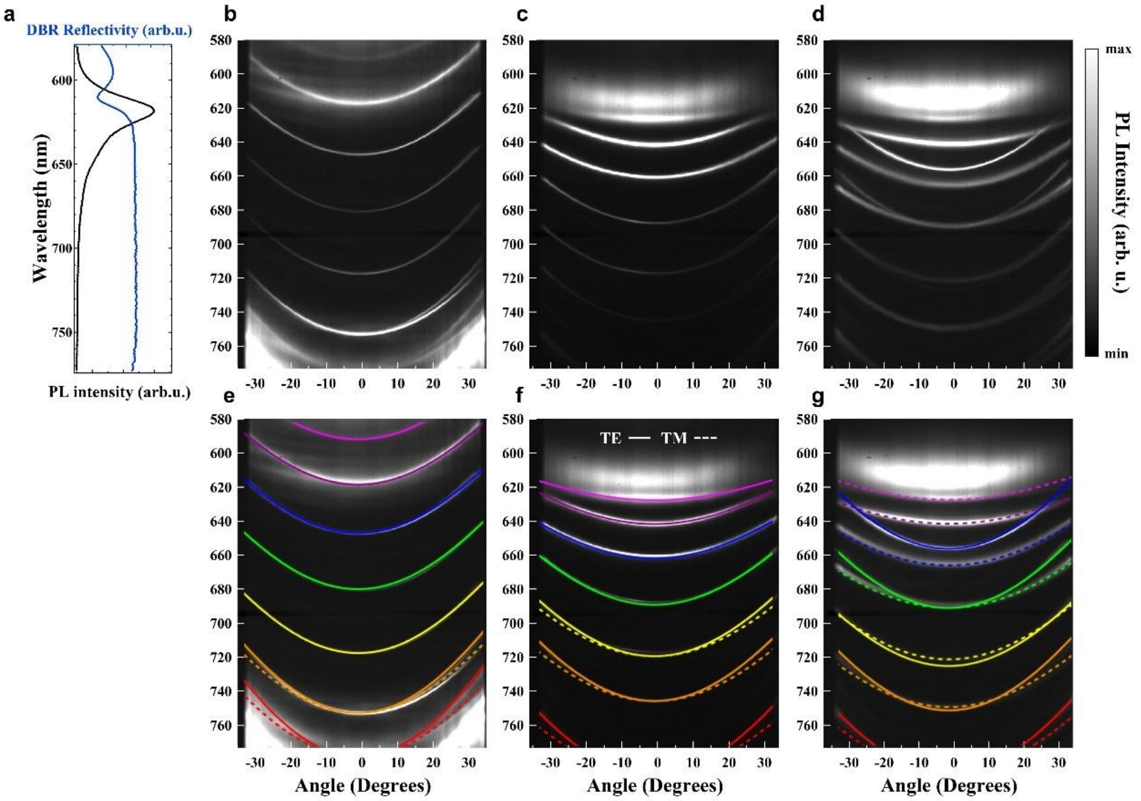

Figure S.23: Angle resolved micro-photoluminescence measurements at room temperature for different points on the second perovskite cavity sample. Here the excitonic resonance is strong, emitting at 618 nm, close to the higher first Bragg mode (**a**). (**b, e**) Multiple modes dispersions with small splittings, indicating a cavity length of 4 μm and an effective refractive index of 1.7. In the simulation (**e**), the colors correspond to different mode numbers, while TE and TM polarization are depicted with solid and dashed lines, respectively. (**c, f**) Similar set of modes with a wavelength dependent curvature and effective refractive index, attributed to the excitonic influence. (**d, g**) Modes with large TE-TM and XY splitting up to 34 meV intersect with each other without interacting. Modes with TM polarization again show smaller curvature close to the exciton wavelength, while those with TE polarization are unaffected.

Angle resolved reflectivity

The cavity modes dispersion and their interaction with the exciton are also measured using angle resolved reflectivity measurements presented in **Figure S.24**. The cavity modes curves supported by the first sample cavity inside its stop band can be observed in **Figure S.24a**. Close to the excitonic resonance, at the higher first Bragg mode, the formation of polaritonic branches can be observed with the flattening of curves for nonzero angles. In **Figure S.24b**, non-interacting mode curves at the edge of the stop band are shown with an equal spectral distance and uniform curvature. Outside the stop band the configuration is more complicated with many modes having varied distance with each other. At a different point of **Figure S.24c**, the modes outside the stopband also have different curvatures, indicating that they are subject to TE-TM and XY splitting and possess different polarizations. At the start of the

stopband, an intense anti-crossing is visible and should be attributed either to strong coupling with the exciton, or to spin-orbit interaction of modes with different polarization and parity.

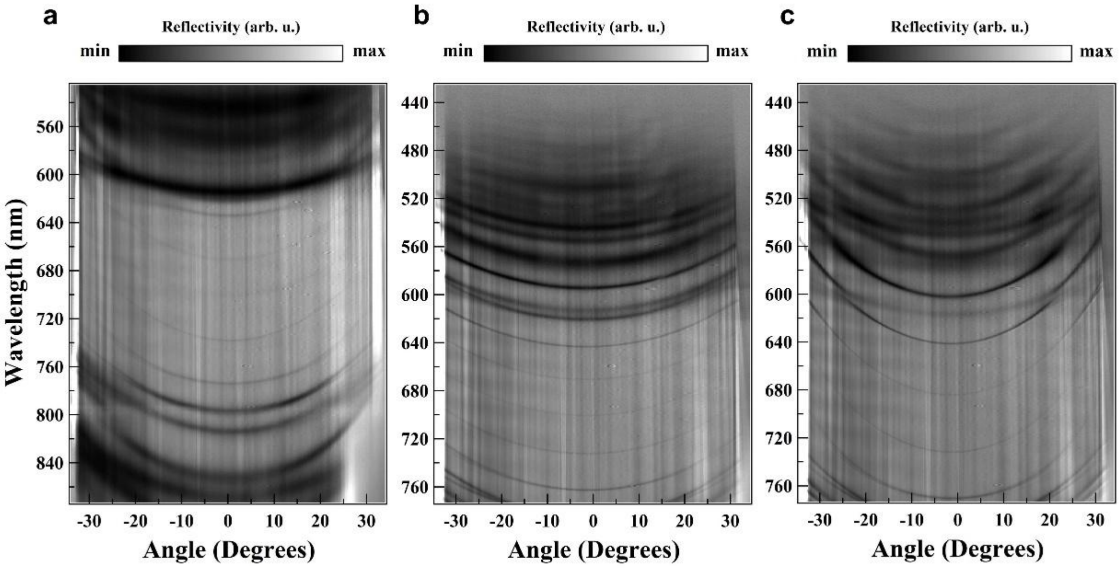

Figure S.24: Angle resolved reflectivity measurements of the second sample at room temperature. (**a**) Equally spaced optical modes with uniform curvature exist inside the stop band of the cavity with a polaritonic interaction visible close to the high first Bragg mode and the excitonic resonance. (**b**) Outside the stop band, for wavelengths lower than $610\ nm$, the modes are more closely positioned with varied distance between them. (**c**) Modes subject to TE-TM and XY splitting outside the stop band with an interaction - strong coupling, spin orbit coupling or both - close to its edge.

S.5 Splitting and interaction of cavity modes and strong coupling

S.5.A Rashba-Dresselhaus interaction

Optical planar microcavities confine light waves that interfere with each other and produce Fabry-Perot modes in their spectral output. The energy of the optical modes is related to their planar wavevector:

$$E(k_{\parallel}) \cong E_{q,ph} + \frac{\hbar^2 k_{\parallel}^2}{2m_{ph}^*}$$

$$m_{ph}^* = \frac{q\pi\hbar n_c}{cL_c} \qquad E_{q,ph} = \frac{q\pi\hbar c}{n_c L_c}$$

In dispersion representation (energy – wavevector), this relation corresponds to a parabolic curve, whose shape and origin point depend on the width L_c and refractive index of the cavity

n_c. The same quantities affect the energy distance between modes of successive cavity numbers q and $q+1$, with larger cavities accommodating modes closer to each other.

When light is incident on the surface of the microcavities at non-zero angle (having non-zero component of the in-plane wavevector), its two base electric field polarizations, TE or s-polarization and TM or p-polarization, experience different reflectivity and transmission coefficients inside the DBRs [93] and their corresponding mode dispersions are split for $k_{\parallel} \neq 0$, with a quadratic dependence on the wavevector.

When the cavity active material is birefringent, as in the case of layered perovskite crystals, light is subject to a further TE-TM-like splitting where orthogonal polarizations inside the cavity (referred to as TE and TM in this work for simplicity) experience different refractive indices [24, 31, 41]. The difference depends on the dielectric tensor of the material but also on the exact orientation of its birefringent crystallographic axes. The anisotropy of the material causes a further splitting of the two polarizations even at $k_{\parallel} \neq 0$, the XY splitting, that can be wavelength dependent.

The combination of these two splitting effects results in the close proximity of modes with different parities and polarization, to the point where they become degenerate at certain wavevectors. At these regions, photonic spin-orbit coupling combined with the optical asymmetry of the cavity can cause the interaction of these modes and the anti-crossing of their dispersion curves, which is a manifestation of Rashba-Dresselhaus effects in an optical system [23], explained in the following paragraphs. The splitting effects and interactions of a set of cavity modes in a birefringent cavity are presented progressively in **Figure S.25**.

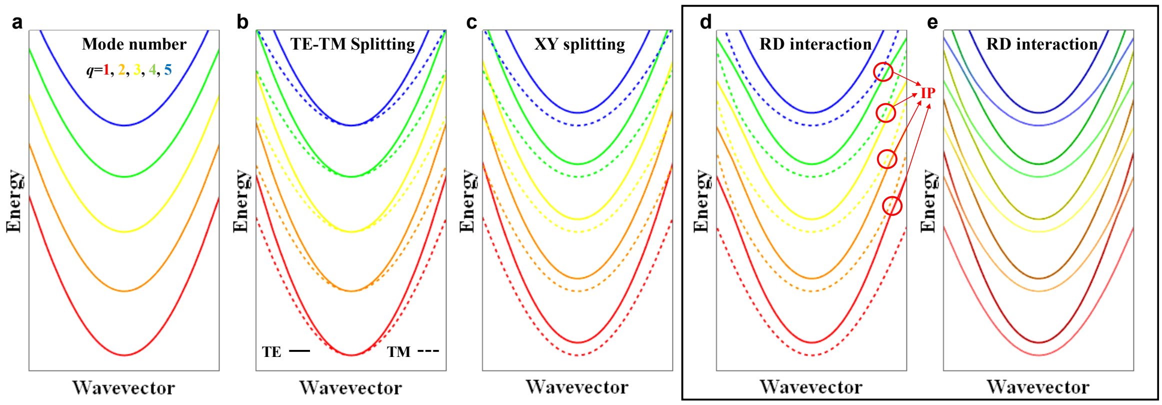

Figure S.25: Optical cavity modes dispersions, splitting effects and Rashba-Dresselhaus interaction. (**a**) Bare cavity modes with parabolic dispersion. Each color represents a different cavity mode with quantum number q. (**b**) TE-TM splitting of cavity modes for nonzero wavevector, with TM polarization shown as dashed lines and TE as solid ones. (**c**) XY splitting of the two polarization states due to the birefringence of the cavity active material. (**d-e**) Rashba- Dresselhaus interaction of modes with different polarization and opposite parity. The red circles in (**d**) denote the anti-crossing or interacting points (IP) of the dispersion curves, (**e**) Close to the interacting points, the dispersion curves are composed of fractions of the interacting modes with a transition occurring at higher energies and wavevectors. Here, the TM polarization of each mode number is depicted with bright colors, while the TM with dark.

The Rashba-Dresselhaus interaction of the photon modes results in distorted dispersions close to the anti-crossing points, where the modes are exchanged for lower and higher energies as shown in **Figures S.25(d, e)**. At the strongest interaction point, the dispersions are composed of both modes with equal contributions.

When the XY splitting has such a value, that modes of opposite parity and polarization become degenerate at $k_{\|} = 0$, as in **Figure S.26**, the modes interact while attaining right $\sigma^{+}$and left σ^{-}circular polarizations and the resulting dispersions shift across the wavelength axis.

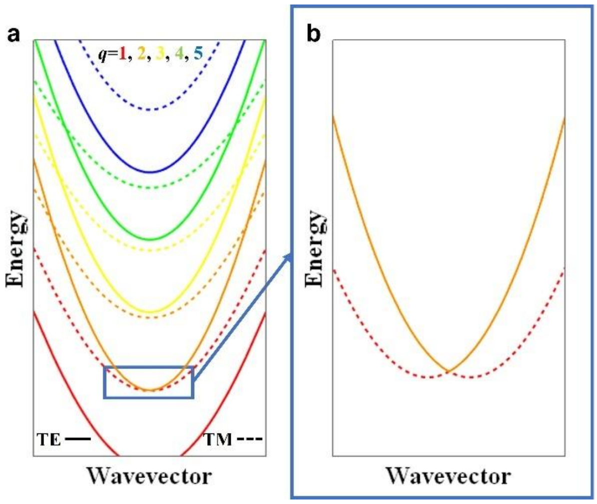

Figure S.26: Shifting of the interacting photonic modes at $k_{\|} = 0$. (**a**) XY splitting can cause modes with different polarization and parity to become degenerate at zero in-plane momentum. (**b**) Zoom-in depiction of the interaction area of (**a**). The two modes are diametrically shifted away from $k_{\|} = 0$. (**c**) The interaction leads to a superposition of the two linear polarized states with a phase factor and thus the modes attain right $\sigma^{+}$and left σ^{-}circular polarization.

S.5.B Polaritonic strong coupling

The inclusion of an excitonic resonance inside the cavity introduces an additional interaction, with each of the photonic eigenstates of the previous section strongly coupling to the exciton thus giving rise to polaritonic states. The resulting dispersion curves, taking into account all effects considered for multiple modes, are shown in **Figure S.27**.

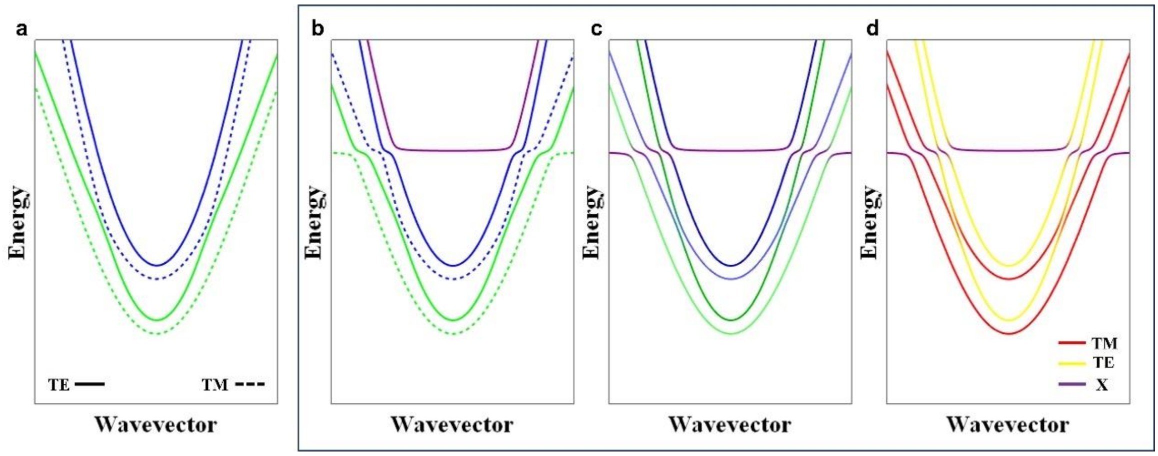

Figure S.27: Inclusion of excitonic resonance in a multiple cavity mode system with Rashba-Dresselhaus interactions. (**a**) The highest energy cavity modes with successive numbers and split polarizations of **Figure S.25d** dispersions. Only modes with different polarizations and parity interact with each other. (**b-d**) Polaritonic strong coupling between an exciton and the photonic modes. (**c**) Excitonic and photonic fraction composition of the dispersion eigenstates. Different colors represent different parities, while light and dark hues, correspond to different polarizations. (**d**) TE and TM polarization parts in the dispersion eigenstates. Red and yellow colors denote TM and TE polarization, respectively. At the interaction points the orange color reveals a mixture of the two linear polarizations.

As in pure photonic or polaritonic systems, the final interacting dispersion eigenstates are a mixture of the initial non-interacting particle eigenfunctions, with each one contributing to a different extent for each value of energy and momentum. For n photonic modes, each of the i final dispersion eigenstates, with $i = n + 1$ accounting for the exciton, can be expressed as:

$$|D_i\rangle = C_x(k)|X\rangle + C_{ph1,X}(k)|Ph_{1,X}\rangle + C_{ph1,Y}(k)|Ph_{1,Y}\rangle + \cdots C_{phn,s}(k)|Ph_{n,s}$$

where $|C_x|^2$ and $|C_{phn,s}|^2$ are respectively the excitonic and photonic fractions, with number n and polarization s, and $|C_x|^2 + |C_{ph1,X}|^2 + |C_{ph1,Y}|^2 + \cdots + |C_{phn,s}|^2 = 1$. The final eigenstate can also be written in terms of the excitonic and the two polarization eigenstates of the system as:

$$|D_i\rangle = C_x(k)|X\rangle + C_{phX}(k)|Ph_{1,X}\rangle + C_{phY}(k)|Ph_{1,Y}\rangle$$

$|C_{phX}|^2$and $|C_{phY}|^2$ are now the fractions of photonic TE and TM polarization of the resulted interaction curve, with $|C_x|^2 + |C_{phX}|^2 + |C_{phY}|^2 = 1$.

S.5.C. Total effective Hamiltonian

The total Hamiltonian for a system with n interacting cavity modes strongly coupled with an exciton resonance can be expressed as:

$$H_{tot} = \begin{pmatrix} E_{ex} & 0 \\ 0 & 0 \end{pmatrix} \otimes I_n + \begin{pmatrix} 0 & 0 \\ 0 & 1 \end{pmatrix} \otimes H_{ph,n} + \frac{\hbar\hat{\sigma}_x}{2} \otimes \begin{pmatrix} \Omega_{1,X} & 0 & \cdots & 0 & 0 \\ 0 & \Omega_{1,Y} & \cdots & 0 & 0 \\ \vdots & \vdots & \ddots & \vdots & \vdots \\ 0 & 0 & \cdots & \Omega_{n,X} & 0 \\ 0 & 0 & \cdots & 0 & \Omega_{n,Y} \end{pmatrix}$$

Each of the n modes are split into $s = X$ or $s = Y$ polarization (TE and TM, respectively) and can strong couple with the exciton, having energy E_{ex}, with a Rabi splitting of $\Omega_{n,s}$. The interaction between the modes is defined by the Hamiltonian term $H_{ph,n}$ which is:

$$H_{ph,n} = \begin{pmatrix} E_{1,X} & 0 & \cdots & A_{(1,m),(X,s)} & \cdots & 0 & A_{(1,n),(X,Y)} \\ 0 & E_{1,Y} & \cdots & A_{(1,m),(Y,s)} & \cdots & A_{(1,n),(Y,X)} & 0 \\ \vdots & \vdots & \ddots & \vdots & \ddots & \vdots & \vdots \\ A_{(m,1),(s,X)} & A_{(m,1),(s,Y)} & \cdots & E_{m,s} & \cdots & A_{(m,n),(s,X)} & A_{(m,n),(s,Y)} \\ \vdots & \vdots & \ddots & \vdots & \ddots & \vdots & \vdots \\ 0 & A_{(n,1),(X,Y)} & \cdots & A_{(n,m),(X,s)} & \cdots & E_{n,X} & 0 \\ A_{(n,1),(Y,X)} & 0 & \cdots & A_{(n,m),(Y,s)} & \cdots & 0 & E_{n,Y} \end{pmatrix}$$

The diagonal elements $E_{m,s}$ are the energies of n interacting modes while the off-diagonal elements $A_{(m,m\prime),(s,s\prime)}$ represent the interaction strength between a mode with number m and polarization s, and another with number m' and polarization s'. These elements are nonzero only when $m \neq m'$ and $s \neq s'$, and their value along with that of $E_{m,s}$ can be calculated by the two-mode interaction Hamiltonian H_{ph}.

S.6 Effective magnetic field

The effective photonic Hamiltonian of the system in circularly polarized basis is written as:

$$H_{ph} = \left(\frac{\hbar^2 k_x^2}{2m_x} + \frac{\hbar^2 k_y^2}{2m_y} + \frac{\hbar^2 k_x k_y}{2m_{xy}}\right)\hat{I} + \left(\delta_x k_x^2 + \delta_y k_y^2 + \delta_{xy} k_x k_y\right)\hat{\sigma}_x + \frac{1}{2}(E_X - E_Y)\hat{\sigma}_x \\ - 2\left(a_x k_x + a_y k_y\right)\hat{\sigma}_z$$

or:

$$H_{ph} = \left(\frac{\hbar^2 k_x^2}{2m_x} + \frac{\hbar^2 k_y^2}{2m_y} + \frac{\hbar^2 k_x k_y}{2m_{xy}}\right)\hat{I} + \mu_B g \boldsymbol{B} \cdot \boldsymbol{\sigma}$$

The momentum-dependent effective magnetic field in the case of the above effective Hamiltonian is:

$$\boldsymbol{B} = (\delta_x k_x^2 + \delta_y k_y^2 + \delta_{xy} k_x k_y + \frac{1}{2}(E_X - E_Y), 0, -2\, a_x k_x - 2\, a_y k_y\,)$$

$$\text{with } \boldsymbol{B_{TE-TM,XY}} = (\,\delta_x k_x^2 + \delta_y k_y^2 + \delta_{xy} k_x k_y + \tfrac{1}{2}(E_X - E_Y), 0{,}0)$$

$$\text{and } \boldsymbol{B_{RD}} = (0{,}0, -2\, a_x k_x - 2\, a_y k_y).$$

By changing the parameters $a_x, a_y, \delta_x, \delta_y, \delta_{xy}, E_X$ and E_Y that depend on the perovskite dielectric tensor elements ε_{xp}, ε_{yp}, ε_{yp}, the crystal Euler angles θ, φ, ψ, the cavity length L_c and the quantum number of the two interacting cavity modes of different parity and polarization, we can simulate the total effective magnetic field at different positions on the samples.

For instance, for the case of the interacting modes of our system (**Figure 2**), the resulting effective magnetic fields for the TE-TM and XY splittings and RD interaction are shown separately and combined in **Figure S.28**.

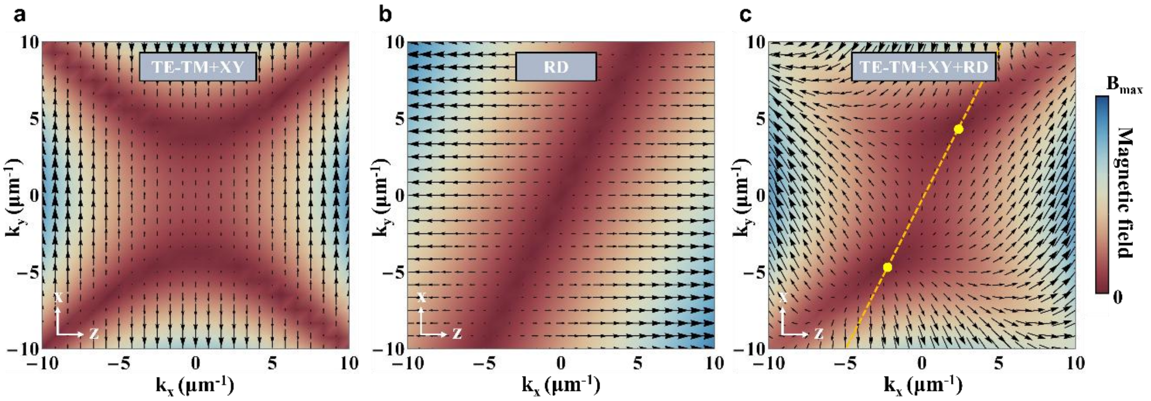

Figure S.28: Calculated effective magnetic fields generated on the k_x, k_y plane due to TE-TM, XY splittings and RD interaction of the two cavity modes of our system. The combination of the effective magnetic field due to TE-TM and XY splittings (**a**) and RD interaction (**b**) results in a total effective magnetic field that is zero for two specific points on the momentum plane: $(2.02\ \mu m^{-1}, 3.97\ \mu m^{-1})$ and $(-2.02\ \mu m^{-1}, -3.97\ \mu m^{-1})$ (**c**). These are the diabolical points of the system and are positioned on a line on the k_x, k_y plane with a slope a_x/a_y, with a_x, a_y the RD parameters of the effective Hamiltonian.

The secular equation of the effective Hamiltonian and the magnetic field with $\mu_B g\boldsymbol{B} \to \boldsymbol{B}$ is:

$$\det\begin{pmatrix} E_i + B_z - E & B_x \\ B_x & E_i - B_z - E \end{pmatrix} = 0$$

where $E_i = \frac{\hbar^2 k_x^2}{2m_x} + \frac{\hbar^2 k_y^2}{2m_y} + \frac{\hbar^2 k_x k_y}{2m_{xy}}$. The two eigenvalues $E_\pm$ are calculated as:

$$E_{\pm} = E_i \pm \sqrt{B_x^2 + B_z^2}$$

The diabolical points are defined as the (anti-)crossing points on the dispersion curves, which indicates $E_+ = E_-$ in our case. Hence $\sqrt{B_x^2 + B_z^2} = 0$, implying the zero effective magnetic field at the diabolical points.

Therefore, the diabolical points of the system emerge at the momentum plane (with $k_x = \pm 2.02\ \mu m^{-1}$ and $k_y = \pm 3.97\ \mu m^{-1}$) where the vector sum of the two fields $\boldsymbol{B}_{TE-TM,XY}$ and $\boldsymbol{B}_{RD}$ is zero and cancel each other. They are positioned at an axis in the k_x, k_y plane with slope dependent on the values of a_x and a_y.

S.7 Berry curvature calculation

The general form of the Hamiltonian of a two-level system, like our effective Hamiltonian, in the spinor representation is:

$$H = E_0 \hat{I} + \boldsymbol{B} \cdot \boldsymbol{\sigma}$$

In spherical coordinates, $\boldsymbol{B} = |\boldsymbol{B}|[\sin(\theta)\cos(\varphi), \sin(\theta)\sin(\varphi), \cos(\theta)]$, with the azimuthal and polar angles defined as: $\varphi = \tan^{-1}\left(\frac{B_y}{B_x}\right)$, and $\theta = \cos^{-1}\left(\frac{B_z}{|\boldsymbol{B}|}\right)$. Thereby, the two corresponding eigenstates of the Hamiltonian are:

$$|-\rangle = \begin{pmatrix} \sin\left(\frac{\theta}{2}\right) \\ -\cos\left(\frac{\theta}{2}\right) e^{i\varphi} \end{pmatrix} \quad |+\rangle = \begin{pmatrix} \cos\left(\frac{\theta}{2}\right) \\ \sin\left(\frac{\theta}{2}\right) e^{i\varphi} \end{pmatrix}$$

Without loss of generality, if we focus on the eigenstate $|-\rangle$, the Berry connection along the three directions would be:

$$V_r = i\langle -|\partial_r|-\rangle = 0$$

$$V_\theta = \frac{i}{|\boldsymbol{B}|}\langle -|\partial_\theta|-\rangle = 0$$

$$\boldsymbol{V} = V_\varphi \hat{\boldsymbol{\varphi}} = \frac{i}{|\boldsymbol{B}|\sin(\theta)}\langle -|\partial_\varphi|-\rangle \hat{\boldsymbol{\varphi}} = -\frac{\cos^2\left(\frac{\theta}{2}\right)}{|\boldsymbol{B}|\sin(\theta)} \hat{\boldsymbol{\varphi}}$$

The Berry curvature, can be calculated by taking the curl of the Berry connection, which is:

$$\boldsymbol{\Omega} = \nabla \times \boldsymbol{V} = \frac{\boldsymbol{B}}{2|\boldsymbol{B}|^3}$$

For the case of the interacting cavity modes of our first sample, it is worth noticing that, because the spin-orbit coupling effect does not break the time reversal symmetry, the effective Hamiltonian is invariant under the time reversal operation of bosonic system, $T = K\sigma_x$. As a result, the Berry curvature is an odd function in momentum space, $\boldsymbol{\Omega}(\boldsymbol{k}) = -\boldsymbol{\Omega}(-\boldsymbol{k})$. Since we are considering the Hamiltonian in the xy plane of the momentum space, the Berry curvature shall only be polarized along the z-axis, i.e., $\boldsymbol{\Omega} = \Omega_z \hat{\boldsymbol{z}}$. By substituting the total effective magnetic field, the Berry curvature Ω can be calculated as:

$$|\boldsymbol{\Omega}| = |\Omega_z \hat{\boldsymbol{z}}| = \frac{B_z}{2|\boldsymbol{B}|^3} = -\frac{a_x k_x + a_y k_y}{\left(4\left(a_x k_x + a_y k_y\right)^2 + \left(\delta_x k_x^2 + \delta_y k_y^2 + \delta_{xy} k_x k_y\right)^2\right)^{3/2}}$$

In **Figure S.29**, the dependence of the simulated photonic modes coupling, effective magnetic field, diabolical points and Berry curvature on the Euler angles θ, φ, ψ is presented for 4 distinct cases of angle combinations. Since the Euler angles are the parameters that can vary the most for different crystals inside the microcavity, they can determine the interaction characteristics. As mentioned in the main text, the diabolical points are positioned along the $k_y = -(a_x/a_y)k_x$ line in the momentum plane. Therefore, the momentum of light at the non-trivial topological states can be determined by the orientation of the crystals through the Euler angles. It is clear that the choice of Euler angles determines the modes coupling, the position of the diabolical points in momentum space and the shape of the Berry curvature. **Figure S.29a** corresponds to the crystal orientation shown in the main text. In **Figure S.29b**, the Euler angles have larger values resulting in larger RD parameters a_x, a_y. Furthermore, the opposite sign of a_y shifts the modes into crossing at an axis with negative slope, and the diabolical points and Berry curvature to have opposite configuration compared to the previous case. In **Figure S.29c**, the only nonzero Euler angle is θ with the crystal being almost parallel to the substrate plane. The photonic interaction is very weak while the appearance of diabolical points and the divergence of Berry curvature occurs only on the $k_x = 0$ axis. For the last case of **Figure S.29d**, diabolical points are not formed and the Berry curvature is not divergent, indicating that non-trivial topological points do not appear for every Euler angles combination. The effective magnetic field, diabolical points appearance and Berry curvature characteristics depending on the Euler angles are also shown in **Supplementary Video 1**.

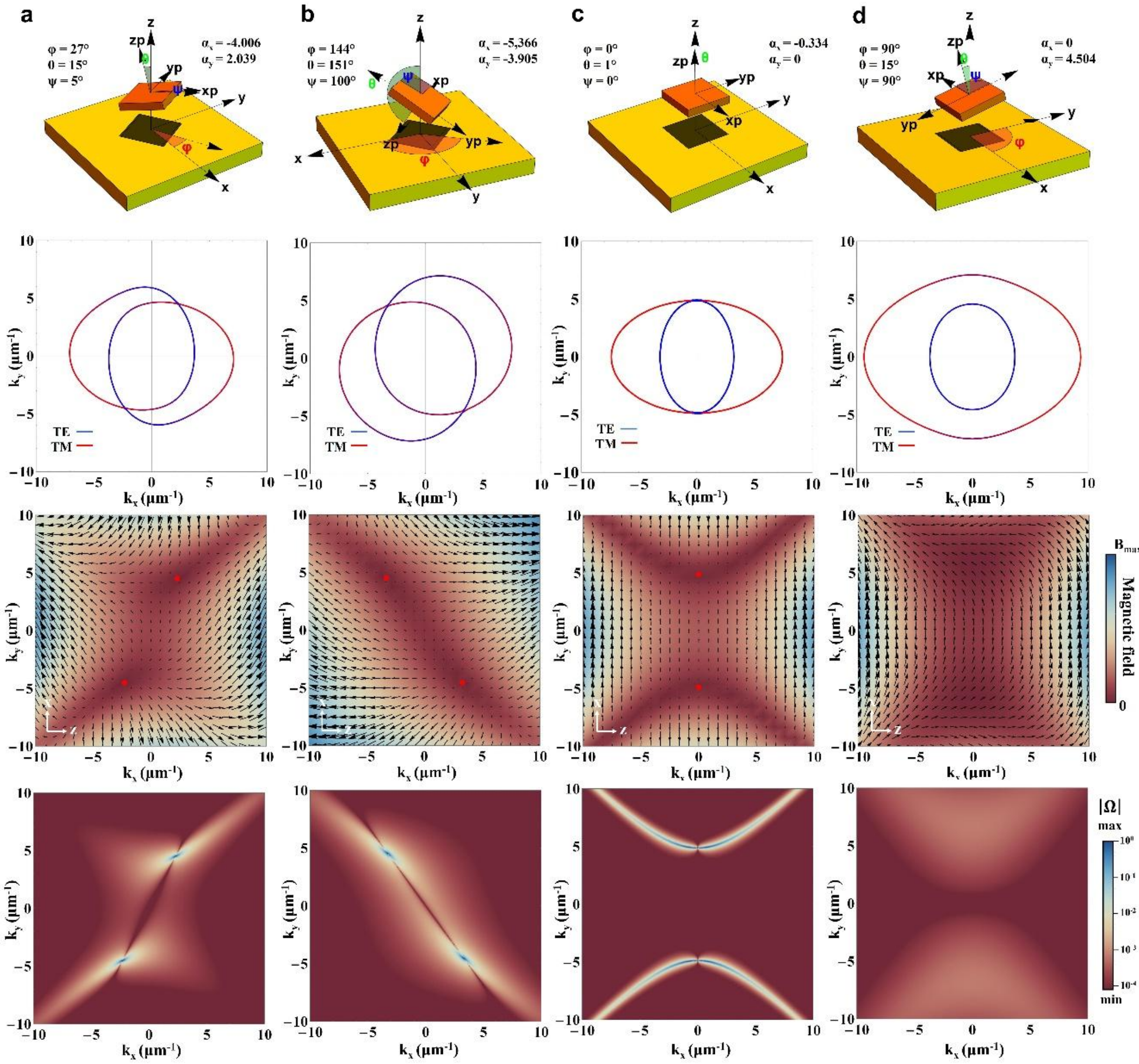

Figure S.29: Perovskite crystal rotation by Euler angles (φ, θ, ψ) inside the microcavity and the respective photonic modes cross section, effective magnetic field with diabolical points (red dots), and Berry curvature. The corresponding Rashba parameters a_x, a_y in meV μm are also presented. (**a**) Crystal orientation as that of the main text. (**b**) Orientation resulting in opposite shifting of the modes, diabolical points of different momentum and distinct Berry curvature. The angles have a larger value, resulting in larger Rashba parameters. (**c**) Crystal almost parallel to the plane of the microcavity, with only θ angle being nonzero, exhibiting modes crossing and diabolical points only on k_x axis. (**d**) Crystal orientation where the modes do not cross, no diabolical points are formed, and the Berry curvature is not divergent.

S.8 Other effects

Owing to the random way the crystals are grown in the cavities, different points on the samples have different properties, such as effective refractive index and cavity length, and exhibit very interesting phenomena in their dispersion measurements. In **Figure S.30**, some of these points are presented. At the first one, of **Figure S.30a**, the spin-orbit interaction is clearly visible, with the anti-crossing of modes at around 20° incident angle. The mode with higher energy also appears flattened for small angles possibly due to the influence of the

exciton at $620\,nm$. By moving to another point in **Figure S.30b**, we can observe the XY splitting of the two polarizations for the higher energy mode. The split curves have slightly different curvature and they do not interact with each other. The lower energy mode has a distorted dispersion due to its interaction with the adjacent mode of opposite parity. In **Figure S.30c** two interacting modes are degenerate in energy and are shifted in a similar manner to that depicted in **Figure S.26**.

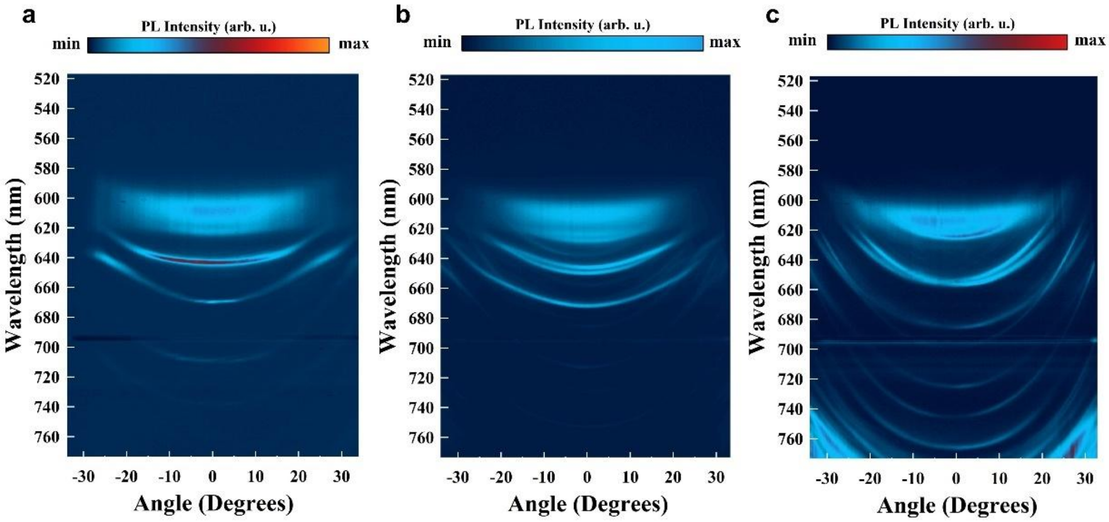

Figure S.30: Angle resolved photoluminescence at room temperature of different points at the first sample exhibiting interesting dispersion shapes and interaction phenomena. (**a**) Rashba-Dresselhaus interaction of two modes, with that of higher energy being flattened at small angles, possibly due to the excitonic influence at $620\,nm$. (**b**) Another point with the upper mode being split in noninteracting TE and TM polarization curves. The lower mode curve is distorted by the interaction with the adjacent mode of opposite parity. (**c**) Another point showing the shifting of two interacting and degenerate modes, an effect depicted in **Figure S26**.

Moving to adjacent points to that of **Figure S.30**, we can observe how the slight change of cavity length can affect the dispersion characteristics of the cavity. At the point of **Figure S.31b**, next to that of **Figure S.30a**, the previous interacting modes have shifted energetically. The flattening of the first mode is not so intense, as its distance to the exciton has increased. Because of that, its curvature also has increased and it does not intersect or interact to the same extent as before with the second mode at higher angles. On the other hand, the XY split mode with the same number but opposite polarization has taken its place close to the excitonic resonance, resulting in strong coupling and the formation of polaritonic branches. The crystal Euler angles for these points are calculated with transfer matrix simulation to be $\theta = 38°$, $\varphi = 42°$, $\psi = 0°$ and $\theta = 33°$, $\varphi = 67°$, $\psi = 0°$, respectively.

The system is dependent on many factors with each crystal having different orientation angle and width. Because of the existence of many cavity modes with small spectral distance and interconnected interactions between them and the exciton, any small change or tuning of these factors can result in a vastly different modification of their dispersion and emergence of various interaction effects.

In **Figure S.31c** the crystal is in such a position that the cavity mode at $640\,nm$ has an asymmetric dispersion curve with an energy gap at zero incident angle and its two parts following different trajectories for positive and negative angles. As the transfer matrix simulation of **Figure S.31f** shows, the anisotropy of the material at that angle ($\theta = 18°,\ \varphi = 8°,\ \psi = -1°$) causes one half of the dispersion of this mode to be more visible than the other, with the opposite configuration occurring for the next mode. As will be shown in the next section, the intensity of the two parts is also dependent on the polarization of the excitation beam.

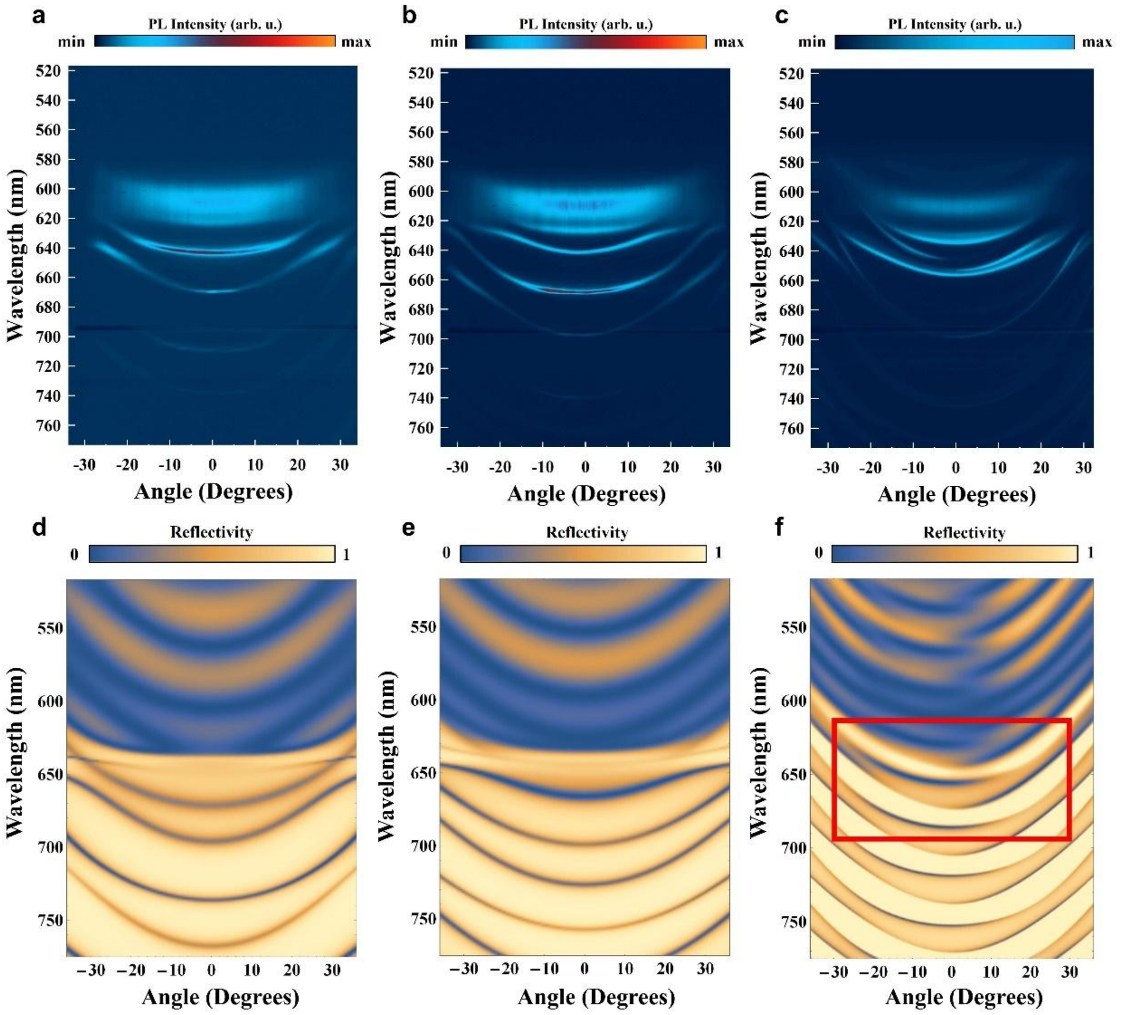

Figure S.31: Angle resolved photoluminescence and reflectivity simulations at room temperature for adjacent points on the second sample. (**a**) Same point as in **Figure S29a** with a characteristic flattened mode (**b**) An adjacent point where the cavity modes have shifted to lower energies. The previous mentioned mode is less flattened and its interaction with the successive mode is less strong. It has been replaced by its XY split mode of opposite polarization at 640 nm that is strongly coupled with the exciton. (**c**) Asymmetric dispersion of cavity modes exhibiting an energy gap between the two parts of the dispersion curve with positive and negative incident angles. The anisotropic interaction causes the modes to appear more intense for different angle signs. (**d-f**) Transfer matrix simulations for the corresponding points (**a-c**), with crystal angles being calculated as $\theta = 38°,\ \varphi = 42°,\ \psi = 0°$, $\theta = 33°,\ \varphi = 67°,\ \psi = 0°$ and $\theta = 18°,\ \varphi = 8°,\ \psi = -1°$, respectively. The red box of (**f**) depicts the asymmetric modes region of (c).

S.8.A Polarization dependence

According to the system Hamiltonian, when cavity modes are subject to both TE-TM and XY splitting, their split states have opposite polarization, TE and TM respectively. In our experimental measurements in the strong coupling regime, the two polarization states are not completely distinguishable due to the influence of exciton that forces the modes to acquire similar curvature at higher angles and the fact that the states are mixed.

On the contrary, the two states can be separated for photonic interaction, as shown in **Figure S.32**. In this case, the angle resolved reflectivity measurements for $k_x = 0$ of a point with Rashba-Dresselhaus interactions close to the higher Bragg mode, at around $600\,nm$, is presented. When we direct the emission through the two sides of a polarizing beam splitter, the cavity states with higher and lower curvature, corresponding to TE and TM polarization, can be separated from each other. The TE-TM splitting of the modes close to the other edge of the stop band, at around $760\,nm$, is also visible.

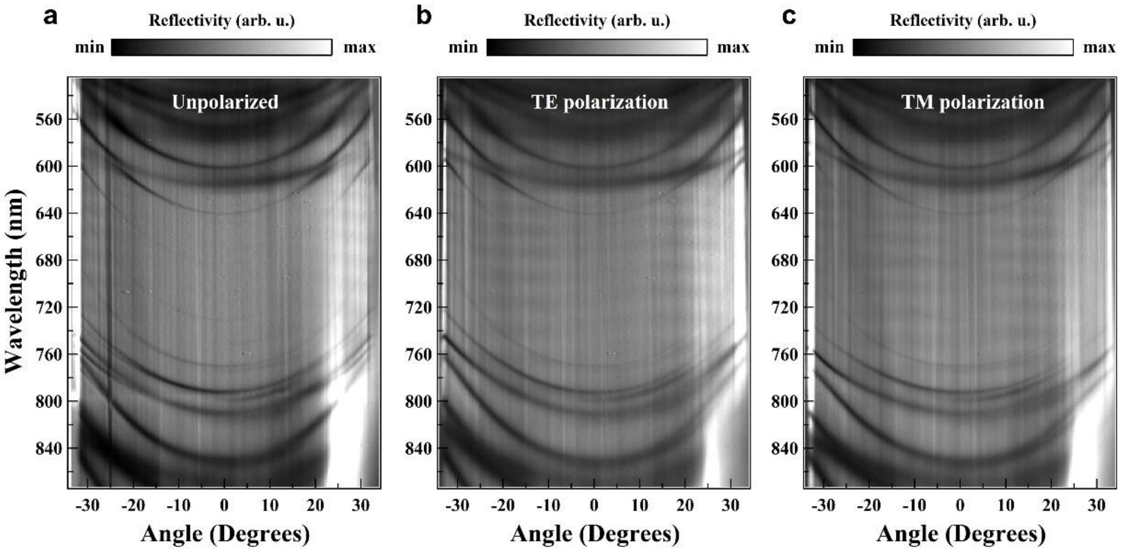

Figure S.32: Angle and polarization resolved reflectivity measurements of the second sample at room temperature. (**a**) Unpolarized dispersion showing TE-TM splitting of modes at the higher edge of the stop band and anti-crossing at the lower edge. TE (**b**) and TM (**c**) polarized emission from the same point. The modes are separated at $760\,nm$, and at $600\,nm$ at the other edge of the stop band.

The photonic interaction should also result in circular polarization splitting where the cavity modes of different polarization acquire opposite circular polarization at the interaction points. This is also measured in our cavities as depicted in **Figure S.33** and **Supplementary Video 2.** There, the XY split modes of different polarization and parity approach each other at various angles, interact, anti-cross and their circular polarization state can be distinguished.

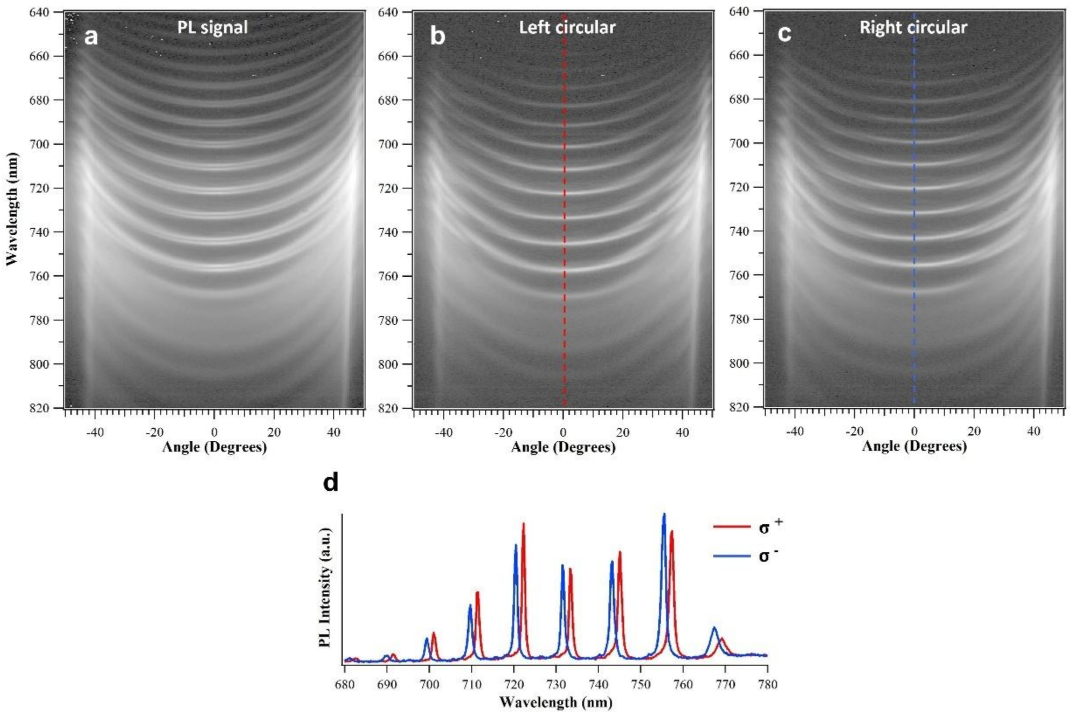

Figure S.33: Angle and circular polarization resolved photoluminescence measurements at room temperature. (a) The unresolved PL emission showing several split cavity modes that interact at k=0 and higher angles. (b) Left and (c) right circular polarization emission of the same point. The modes anti-cross at the interaction points and acquire opposite circular polarization. (**d**) Left (σ^{+}) and right (σ^{-}) polarized emission for $k = 0$ of the split modes.

The dispersion characteristics at specific points, such as that of **Figure S.31c**, depend also on the excitation beam polarization. The laser excitation is normally horizontally polarized. When it passes through the BBO and its frequency is doubled, its polarization changes to elliptical as the anisotropic structure of the BBO causes the electric field components on its crystallographic axes to be delayed by a different extent. By placing a polarizer in front of the sample and rotating its optical axis, we can change the polarization of the incident light. In **Figure S.34** the different dispersions of various excitation polarizations are presented for points on the sample with an asymmetric cavity dispersion curve, as in **Figure S.31c**. For the first point (**Figures S.34a-c**), a polarizer rotation of 97° to the horizontal polarization axis, results in the right part of the dispersion (i.e., the one for positive incident angles), to have maximum intensity, while the left one has minimum intensity. For 50° rotation the situation is reversed with the left part of the modes having maximum intensity, while the right almost disappearing. The same trend is followed at the point of **Figures S.34d-f**, with the right and left parts of the cavity modes having maximum and minimum intensity respectively at 105° polarizer rotation angle, while the reversed configuration occurs at 65° rotation angle.

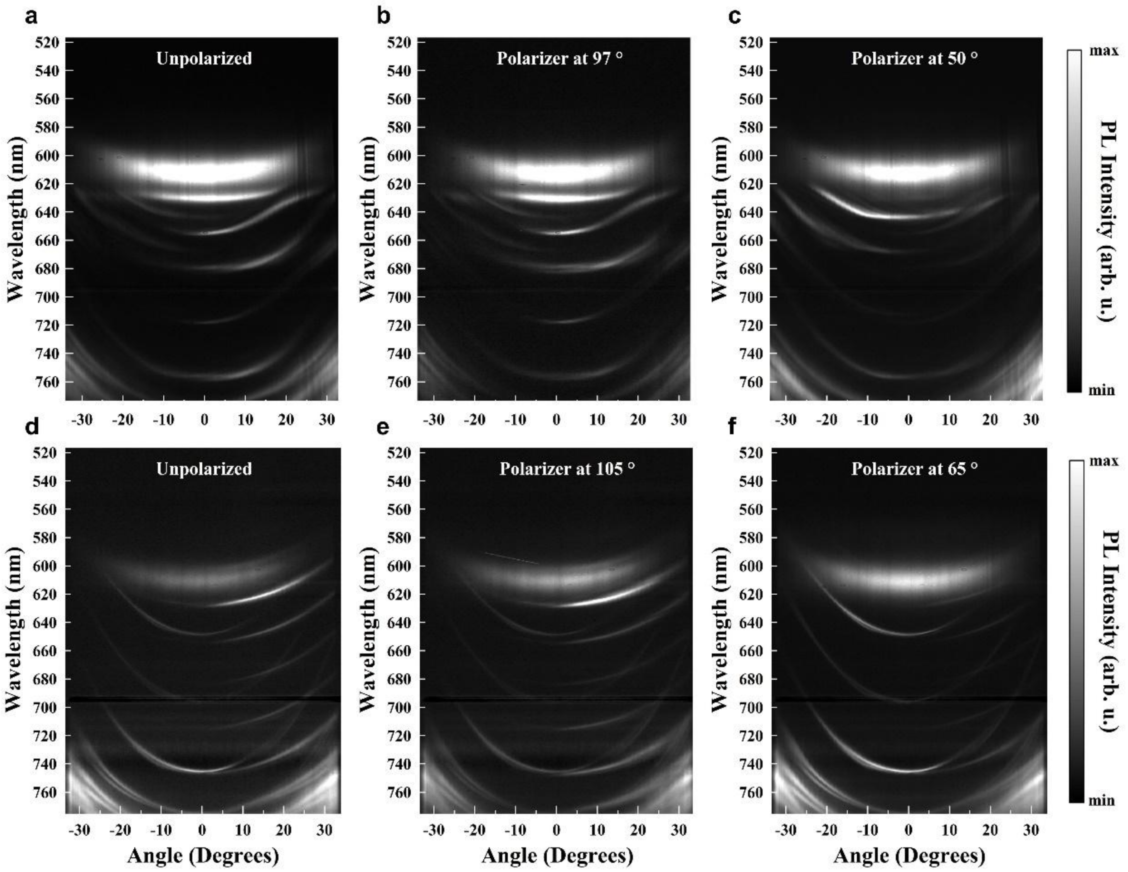

Figure S.34: Angle resolved and excitation polarization dependent photoluminescence at room temperature on points at the second sample showing some asymmetric cavity modes, as in **Figure S.31c**. (**a-c**) First point with (**a**) showing the collected emission without using a polarizer at the excitation beam. The cavity modes split to a right and left side by an energy gap at zero momentum. (**b**) A polarizer is placed with its optical axis rotated at 97° angle to the horizontal polarization of the laser beam. For positive angles of incidence, the modes exhibit maximum intensity, whereas for negative incidence angles, the intensity is minimum. (**c**) At 50° polarizer angle, the reversed situation occurs. (**d-f**) Similar behavior for another point, with right and left sides of the dispersion having maximum and minimum intensities respectively at 105° polarizer angle (**e**) and reversed at 65° polarizer angle (**f**).

There is a difference in emission when two-dimensional perovskites are excited by light with polarization parallel and perpendicular to their stacking axis. In our case, of elliptically polarized excitation and tilted perovskite crystals, there are components of light parallel to the stacking and plane axes of the crystals, E_z and E_{xy}, respectively. Because of TE-TM and XY splitting effects, the system states with different polarizations are separated in dispersion measurements and, as confirmed from the transfer matrix simulation of **Figure S.31f**, exhibit the asymmetric shapes of **Figure S.34**. Each of the two sides of the dispersion curves are associated with E_z and E_{xy} and are influenced more by one or the other. When we place a polarizer in front of the sample, we can tune the relative magnitude of these two components and also the emission intensity of the dispersion curves. This empirical explanation of the phenomenon presented in **Figure S.34** proposes just a qualitative reasoning for its origin and would require additional and more detailed experimental evidence to be confirmed.

S.8.B Confinement effects

When measuring some points on the second sample, their dispersion exhibits cavity modes that are segregated, such as in the case of **Figure S.35**. This phenomenon appears when the system confinement is extended to the xy plane of the cavity and not only on the perpendicular axis. The small lateral size of perovskite crystals can create this type of in-plane confinement and results in segmented dispersion curves. The spectral distance of these segments can provide the spatial dimensions of the crystals. With an effective refractive index of 1.7 the dimensions of the crystals corresponding to the dispersion measurements presented in **Figure S.35** are $41\ \mu m, 26\ \mu m, 60\ \mu m$ and $23\ \mu m,$ respectively, sizes that are consistent with those of the crystals shown with microscope imaging. In some points we can also observe that some cavity modes are solid and are positioned between the dashed ones. These are presumably the XY split modes with opposite polarization to that of the segmented ones, associated with the in-plane axis perpendicular to the confinement axis. They indicate that, at these points, the crystals have a rectangle shape and the light confinement of their larger side is not so strong.

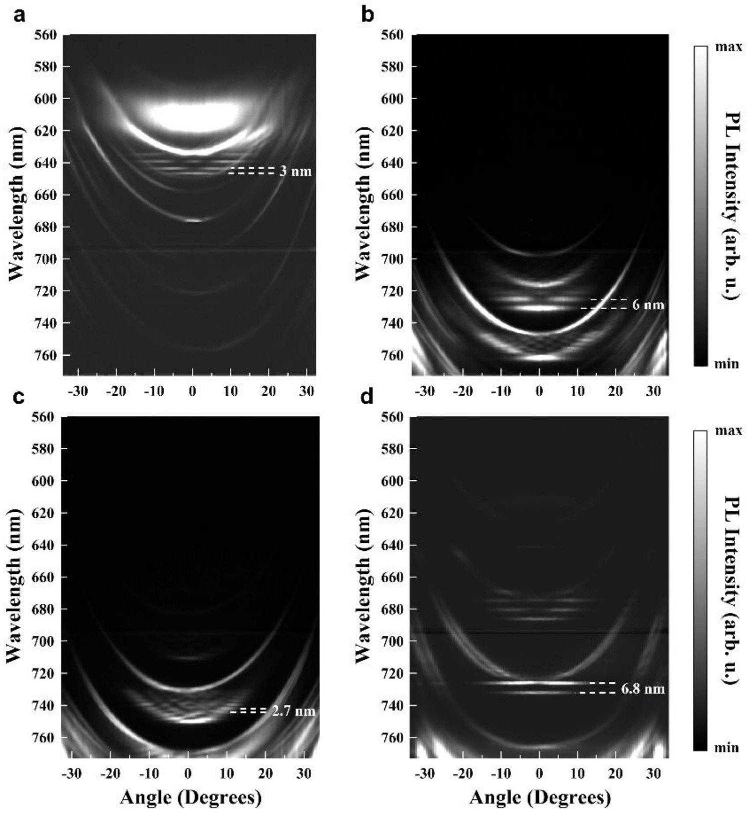

Figure S.35: Angle resolved photoluminescence at room temperature for some points at the second sample showing confinement effects. Some of the dispersion curves are dashed because of the confinement of light at the xy plane of the cavity due to the small perovskite crystals dimensions. The XY split modes do not appear segmented because they are associated with the larger side of the orthogonally shaped crystals that is characterized by weak confinement. The sides of the crystals that do show confinement have lengths calculated as $41\ \mu m$ (**a**), $26\ \mu m$ (**b**), $60\ \mu m$ (**c**) and $23\ \mu m$ (**d**).

Some additional confining features emerge when we measure the dispersion at specific points of the sample inside a cryostat at 13 K, presented in **Figure S.36**. In these cases, between the normal cavity modes there exist additional lines, dashed or solid, that for some energies seem to even have negative curvature. The origin of these effects is not understood completely and further studies are required to reveal their exact nature. However, they appear to be the result of either confined states, more easily observable at low temperatures, or trap states at the edges of the crystals, associated with structural imperfections and defects.

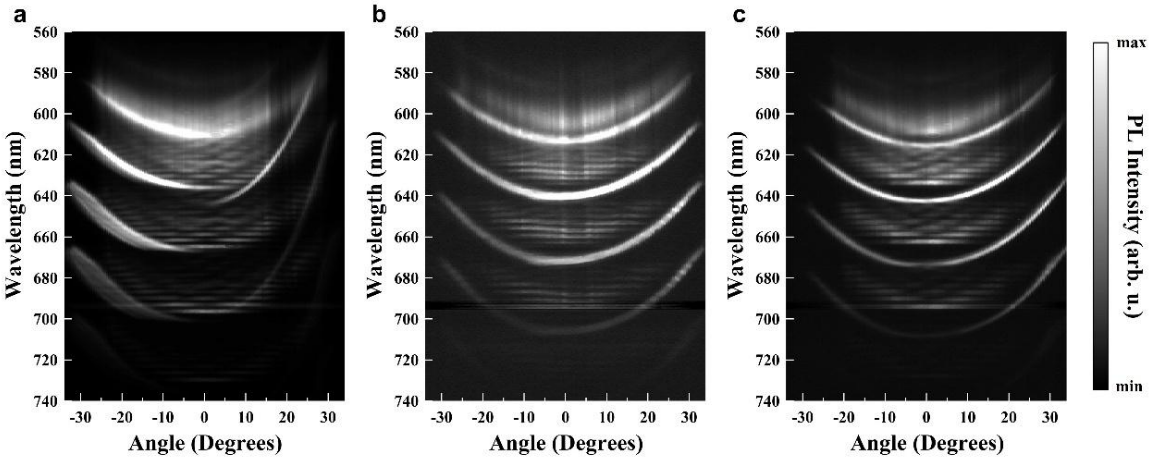

Figure S.36: Angle resolved photoluminescence at 13 K at specific points on the second sample showing additional confinement effects. Segmented lines appear between the normal cavity modes that can be solid (**b**), dashed (**a, c**) or even have negative curvature (**b**). They are attributed to either confined or trap states of the perovskite crystals.

Part B. Hamiltonian and Transfer matrix calculation

S.9 Derivation of the generalized Rashba-Dresselhaus Hamiltonian for a birefringent microcavity

This section presents the derivation of the generalized Hamiltonian for an optical cavity filled with a uniaxial dielectric material, having its birefringence axes rotated at a random direction with respect to the microcavity plane, defined by three Euler angles. The derivation is based on that of reference [31], where the birefringent material is rotated by only one angle. It assumes the cavity being composed of perfect conducting mirrors separated by a distance L, the cavity width, with the z-axis of the laboratory coordinate system perpendicular and the x and y axes parallel to the plane of the mirrors, respectively. For an electromagnetic wave inside the cavity, Maxwell's equations connect the electric fields $\boldsymbol{E}, \boldsymbol{D}$ with the magnetic $\boldsymbol{B}, \boldsymbol{H}$ as:

$$\boldsymbol{\nabla} \times \boldsymbol{E}(\boldsymbol{r}, t) = -\frac{\partial \boldsymbol{B}(\boldsymbol{r},t)}{\partial t} \qquad \boldsymbol{\nabla} \times \boldsymbol{H} = \frac{\partial \boldsymbol{D}}{\partial t} \tag{A.1-2}$$

Analytically these can be written in three dimensions as:

$$\begin{vmatrix} \hat{\boldsymbol{x}} & \hat{\boldsymbol{y}} & \hat{\boldsymbol{z}} \\ \frac{\partial}{\partial x} & \frac{\partial}{\partial y} & \frac{\partial}{\partial z} \\ E_x & E_y & E_z \end{vmatrix} = -\frac{\partial B_x(\boldsymbol{r},t)}{\partial t}\hat{\boldsymbol{x}} - \frac{\partial B_y(\boldsymbol{r},t)}{\partial t}\hat{\boldsymbol{y}} - \frac{\partial B_z(\boldsymbol{r},t)}{\partial t}\hat{\boldsymbol{z}} \tag{A.3}$$

$$\begin{vmatrix} \hat{\boldsymbol{x}} & \hat{\boldsymbol{y}} & \hat{\boldsymbol{z}} \\ \frac{\partial}{\partial x} & \frac{\partial}{\partial y} & \frac{\partial}{\partial z} \\ H_x & H_y & H_z \end{vmatrix} = \frac{\partial D_x(\boldsymbol{r},t)}{\partial t}\hat{\boldsymbol{x}} + \frac{\partial D_y(\boldsymbol{r},t)}{\partial t}\hat{\boldsymbol{y}} + \frac{\partial D_z(\boldsymbol{r},t)}{\partial t}\hat{\boldsymbol{z}} \tag{A.4}$$

Because the fields can be considered as a combination of plane waves, they can be written as:

$$\boldsymbol{B}(\boldsymbol{r}, t) = \boldsymbol{B_0} e^{i(\boldsymbol{k}\cdot\boldsymbol{r} - \omega t)} \qquad \boldsymbol{D}(\boldsymbol{r}, t) = \boldsymbol{D_0} e^{i(\boldsymbol{k}\cdot\boldsymbol{r} - \omega t)} \tag{A.5-6}$$

and (A.3), (A.4) can be modified into six time-independent equations:

$$-\frac{\partial E_y(\boldsymbol{r})}{\partial z}+\frac{\partial E_z(\boldsymbol{r})}{\partial y}=i\omega B_x(\boldsymbol{r}) \tag{A.7}$$

$$\frac{\partial E_x(\boldsymbol{r})}{\partial z}-\frac{\partial E_z(\boldsymbol{r})}{\partial x}=i\omega B_y(\boldsymbol{r}) \tag{A.8}$$

$$-\frac{\partial E_x(\boldsymbol{r})}{\partial y}+\frac{\partial E_y(\boldsymbol{r})}{\partial x}=i\omega B_z(\boldsymbol{r}) \tag{A.9}$$

$$\frac{\partial H_y(\boldsymbol{r})}{\partial z}-\frac{\partial H_z(\boldsymbol{r})}{\partial y}=i\omega D_x(\boldsymbol{r}) \tag{A.10}$$

$$-\frac{\partial H_x(\boldsymbol{r})}{\partial z}+\frac{\partial H_z(\boldsymbol{r})}{\partial x}=i\omega D_y(\boldsymbol{r}) \tag{A.11}$$

$$\frac{\partial H_x(\boldsymbol{r})}{\partial y}-\frac{\partial H_y(\boldsymbol{r})}{\partial x}=i\omega D_z(\boldsymbol{r}) \tag{A.12}$$

or written in a matrix form:

$$\begin{bmatrix} 0 & 0 & 0 & 0 & \frac{\partial}{\partial z} & -\frac{\partial}{\partial y} \\ 0 & 0 & 0 & -\frac{\partial}{\partial z} & 0 & \frac{\partial}{\partial x} \\ 0 & 0 & 0 & \frac{\partial}{\partial y} & -\frac{\partial}{\partial x} & 0 \\ 0 & -\frac{\partial}{\partial z} & \frac{\partial}{\partial y} & 0 & 0 & 0 \\ \frac{\partial}{\partial z} & 0 & -\frac{\partial}{\partial x} & 0 & 0 & 0 \\ -\frac{\partial}{\partial y} & \frac{\partial}{\partial x} & 0 & 0 & 0 & 0 \end{bmatrix} \begin{bmatrix} E_x(\boldsymbol{r}) \\ E_y(\boldsymbol{r}) \\ E_z(\boldsymbol{r}) \\ H_x(\boldsymbol{r}) \\ H_y(\boldsymbol{r}) \\ H_z(\boldsymbol{r}) \end{bmatrix} = i\omega \begin{bmatrix} D_x(\boldsymbol{r}) \\ D_y(\boldsymbol{r}) \\ D_z(\boldsymbol{r}) \\ B_x(\boldsymbol{r}) \\ B_y(\boldsymbol{r}) \\ B_z(\boldsymbol{r}) \end{bmatrix} \tag{A.13}$$

and into shorthand notation:

$$\bar{R}\boldsymbol{G}=i\omega\boldsymbol{C} \tag{A.14}$$

The fields $\boldsymbol{D}$ and $\boldsymbol{B}$ can be eliminated using the expressions for permittivity and permeability:

$$\boldsymbol{D}=\varepsilon\boldsymbol{E} \qquad \boldsymbol{B}=\mu\boldsymbol{H} \tag{A.15-16}$$

In the general case, the permittivity is the material's direction dependent dielectric function, defined as a tensor:

$$\begin{bmatrix} D_{xp} \\ D_{yp} \\ D_{zp} \end{bmatrix} = \begin{bmatrix} \varepsilon_{xp} & 0 & 0 \\ 0 & \varepsilon_{yp} & 0 \\ 0 & 0 & \varepsilon_{zp} \end{bmatrix} \begin{bmatrix} E_{xp} \\ E_{yp} \\ E_{zp} \end{bmatrix} \tag{A.17}$$

with the diagonal terms being the tensor elements of the material along the principal axes. They can be written in terms of the principal refractive indices as:

$$\varepsilon_{xp} = \varepsilon_0 n_{xp}^2 \quad \varepsilon_{yp} = \varepsilon_0 n_{yp}^2 \quad \varepsilon_{zp} = \varepsilon_0 n_{zp}^2 \qquad \text{(A.18-20)}$$

If, $n_{xp} = n_{yp} = n_{zp}$ the material is isotropic, otherwise, it is birefringent. If $n_{xp} = n_{yp} \neq n_{zp}$ it is uniaxial, while, if $n_{xp} \neq n_{yp} \neq n_{zp}$ it is biaxial. In non-magnetic materials, the permeability is equal to the constant permeability of vacuum, thus the magnetic fields are connected with the equations:

$$B_x = \mu_0 H_x \quad B_y = \mu_0 H_y \quad B_z = \mu_0 H_z \qquad \text{(A.21-23)}$$

When the reference coordinate system is different than the principal coordinate system of the material, the permittivity matrix has non-zero off-diagonal elements and equation (A.17) is modified into:

$$\begin{bmatrix} D_x \\ D_y \\ D_z \end{bmatrix} = \begin{bmatrix} \varepsilon_{xx} & \varepsilon_{xy} & \varepsilon_{xz} \\ \varepsilon_{yx} & \varepsilon_{yy} & \varepsilon_{yz} \\ \varepsilon_{zx} & \varepsilon_{zy} & \varepsilon_{zz} \end{bmatrix} \begin{bmatrix} E_x \\ E_y \\ E_z \end{bmatrix} \tag{A.24}$$

The transformation from the material's coordinate system (x_p, y_p, z_p) to the general laboratory system (x, y, z) can be achieved using the transformation matrix $\bar{N}$:

$$\begin{bmatrix} x_p \\ y_p \\ z_p \end{bmatrix} = \bar{N} \begin{bmatrix} x \\ y \\ z \end{bmatrix} \tag{A.25}$$

The matrix $\bar{N}$ is determined by three counterclockwise rotations: (1) around the z axis with angle φ, (2) around the intermediate x axis with angle θ and (3) again around the z axis with angle ψ. These are called Euler's angles and represent the rotation of an orthogonal axis system of a body relative to another reference system. Analytically the matrix $\bar{N}$ can be written as:

$$\bar{N} = \begin{bmatrix} \cos\psi & \sin\psi & 0 \\ -\sin\psi & \cos\psi & 0 \\ 0 & 0 & 1 \end{bmatrix} \begin{bmatrix} 1 & 0 & 0 \\ 0 & \cos\theta & \sin\theta \\ 0 & -\sin\theta & \cos\theta \end{bmatrix} \begin{bmatrix} \cos\varphi & \sin\varphi & 0 \\ -\sin\varphi & \cos\varphi & 0 \\ 0 & 0 & 1 \end{bmatrix} \quad \text{(A.26)}$$

Using this notation, the elements of the dielectric matrix of equation (A.24) can be written in terms of the rotation angles and principal dielectric functions as:

$$\varepsilon_{xx} = \varepsilon_{xp}(\cos\psi\cos\varphi - \cos\theta\sin\varphi\sin\psi)^2 + \varepsilon_{yp}(-\sin\psi\cos\varphi - \cos\theta\sin\varphi\cos\psi)^2 + \varepsilon_{zp}(\sin\theta\sin\varphi)^2 \quad \text{(A.27)}$$

$$\varepsilon_{xy} = \varepsilon_{xp}(\cos\psi\cos\varphi - \cos\theta\sin\varphi\sin\psi)(\cos\psi\sin\varphi + \cos\theta\cos\varphi\sin\psi) + \varepsilon_{yp}(-\sin\psi\cos\varphi - \cos\theta\sin\varphi\cos\psi)(-\sin\psi\sin\varphi + \cos\theta\cos\varphi\cos\psi) + \varepsilon_{zp}(\sin\theta\sin\varphi)(-\sin\theta\cos\varphi) \quad \text{(A.28)}$$

$$\varepsilon_{xz} = \varepsilon_{xp}(\cos\psi\cos\varphi - \cos\theta\sin\varphi\sin\psi)(\sin\psi\sin\theta) + \varepsilon_{yp}(-\sin\psi\cos\varphi - \cos\theta\sin\varphi\cos\psi)(\cos\psi\sin\theta) + \varepsilon_{zp}(\sin\theta\sin\varphi)\cos\theta \quad \text{(A.29)}$$

$$\varepsilon_{yx} = \varepsilon_{xy} \quad \text{(A.30)}$$

$$\varepsilon_{yy} = \varepsilon_{xp}(\cos\psi\sin\varphi + \cos\theta\cos\varphi\sin\psi)^2 + \varepsilon_{yp}(-\sin\psi\sin\varphi + \cos\theta\cos\varphi\cos\psi)^2 + \varepsilon_{zp}(-\sin\theta\cos\varphi)^2 \quad \text{(A.31)}$$

$$\varepsilon_{yz} = \varepsilon_{xp}(\cos\psi\sin\varphi + \cos\theta\cos\varphi\sin\psi)(\sin\psi\sin\theta) + \varepsilon_{yp}(-\sin\psi\sin\varphi + \cos\theta\cos\varphi\cos\psi)(\cos\psi\sin\theta) + \varepsilon_{zp}(-\sin\theta\cos\varphi)\cos\theta \quad \text{(A.32)}$$

$$\varepsilon_{zx} = \varepsilon_{xz} \quad \text{(A.33)}$$

$$\varepsilon_{zy} = \varepsilon_{yz} \quad \text{(A.34)}$$

$$\varepsilon_{zz} = \varepsilon_{xp}(\sin\psi\sin\theta)^2 + \varepsilon_{yp}(\cos\psi\sin\theta)^2 + \varepsilon_{zp}(\cos\theta)^2 \quad \text{(A.35)}$$

The general dielectric functions can be inserted in the matrix $\bar{M}$ together with the material's permeability:

$$\bar{M} = \begin{bmatrix} \varepsilon_{xx} & \varepsilon_{xy} & \varepsilon_{xz} & 0 & 0 & 0 \\ \varepsilon_{yx} & \varepsilon_{yy} & \varepsilon_{yz} & 0 & 0 & 0 \\ \varepsilon_{zx} & \varepsilon_{zy} & \varepsilon_{zz} & 0 & 0 & 0 \\ 0 & 0 & 0 & \mu_0 & 0 & 0 \\ 0 & 0 & 0 & 0 & \mu_0 & 0 \\ 0 & 0 & 0 & 0 & 0 & \mu_0 \end{bmatrix} \quad \text{(A.36)}$$

The fields of equations (A.15-16) can then be expressed as:

$$\boldsymbol{C} = \bar{M}\boldsymbol{G} \tag{A.37}$$

Combining equations (A.14) and (A.37) we have:

$$\bar{R}\boldsymbol{G} = i\omega\bar{M}\boldsymbol{G} \tag{A.38}$$

The plane wave character of the fields allows as to separate their spatial components at x and y axes:

$$\boldsymbol{G} = \begin{bmatrix} E_x(\boldsymbol{r}) \\ E_y(\boldsymbol{r}) \\ E_z(\boldsymbol{r}) \\ H_x(\boldsymbol{r}) \\ H_y(\boldsymbol{r}) \\ H_z(\boldsymbol{r}) \end{bmatrix} = e^{ik_x x} e^{ik_y y} \begin{bmatrix} E_x(z) \\ E_y(z) \\ E_z(z) \\ H_x(z) \\ H_y(z) \\ H_z(z) \end{bmatrix} \tag{A.39}$$

Equation (A.38) can then be reduced to six equations, with two of them not being differential:

$$\frac{\partial H_y(z)}{\partial z} - ik_y H_z(z) = i\omega\big(E_x(z)\varepsilon_{xx} + E_y(z)\varepsilon_{xy} + E_z(z)\varepsilon_{xz}\big) \tag{A.40}$$

$$-\frac{\partial H_x(z)}{\partial z} + ik_x H_z(z) = i\omega\big(E_x(z)\varepsilon_{yx} + E_y(z)\varepsilon_{yy} + E_z(z)\varepsilon_{yz}\big) \tag{A.41}$$

$$ik_y H_x(z) - ik_x H_y(z) = i\omega\big(E_x(z)\varepsilon_{zx} + E_y(z)\varepsilon_{zy} + E_z(z)\varepsilon_{zz}\big) \tag{A.42}$$

$$-\frac{\partial E_y(z)}{\partial z} + ik_y E_z(z) = i\omega H_x(z)\mu_0 \tag{A.43}$$

$$\frac{\partial E_x(z)}{\partial z} - ik_x E_z(z) = i\omega H_y(z)\mu_0 \tag{A.44}$$

$$-ik_y E_x(z) + ik_x E_y(z) = i\omega H_z(z)\mu_0 \tag{A.45}$$

The non-differential equations (A.42) and (A.45) are used to eliminate the longitudinal variables resulting in a system of four equations:

$$\frac{\partial E_x(z)}{\partial z} = \left(-\frac{k_x}{k_0}\frac{\varepsilon_{zx}}{\varepsilon_{zz}}\right) ik_0E_x(z) + \left(-\frac{k_x}{k_0}\frac{\varepsilon_{zy}}{\varepsilon_{zz}}\right) ik_0E_y(z) + \left(\frac{k_xk_y\eta_0}{k_0^2\,\varepsilon_{zz}/\varepsilon_0}\right) ik_0H_x(z) + \left(\frac{(\varepsilon_{zz}/\varepsilon_0 - k_x^2/k_0^2)\eta_0}{\varepsilon_{zz}/\varepsilon_0}\right) ik_0H_y(z) \tag{A.46}$$

$$\frac{\partial E_y(z)}{\partial z} = \left(-\frac{k_y}{k_0}\frac{\varepsilon_{zx}}{\varepsilon_{zz}}\right) ik_0E_x(z) + \left(-\frac{k_y}{k_0}\frac{\varepsilon_{zy}}{\varepsilon_{zz}}\right) ik_0E_y(z) + \left(\frac{\left(k_y^2/k_0^2 - \varepsilon_{zz}/\varepsilon_0\right)\eta_0}{\varepsilon_{zz}/\varepsilon_0}\right) ik_0H_x(z) + \left(-\frac{k_xk_y\eta_0}{k_0^2\,\varepsilon_{zz}/\varepsilon_0}\right) ik_0H_y(z) \tag{A.47}$$

$$\frac{\partial H_x(z)}{\partial z} = \left[\frac{1}{\eta_0}\left(-\frac{\varepsilon_{yx}}{\varepsilon_0} - \frac{k_xk_y}{k_0^2} + \frac{\varepsilon_{yz}\varepsilon_{zx}}{\varepsilon_{zz}\varepsilon_0}\right)\right] ik_0E_x(z) + \left[\frac{1}{\eta_0}\left(\frac{k_x^2}{k_0^2} - \frac{\varepsilon_{yy}}{\varepsilon_0} + \frac{\varepsilon_{yz}\varepsilon_{zy}}{\varepsilon_{zz}\varepsilon_0}\right)\right] ik_0E_y(z) + \left(-\frac{k_y}{k_0}\frac{\varepsilon_{yz}}{\varepsilon_{zz}}\right) ik_0H_x(z) + \left(\frac{k_x}{k_0}\frac{\varepsilon_{yz}}{\varepsilon_{zz}}\right) ik_0H_y(z) \tag{A.48}$$

$$\frac{\partial H_y(z)}{\partial z} = \left[\frac{1}{\eta_0}\left(-\frac{k_y^2}{k_0^2} + \frac{\varepsilon_{xx}}{\varepsilon_0} - \frac{\varepsilon_{xz}\varepsilon_{zx}}{\varepsilon_{zz}\varepsilon_0}\right)\right] ik_0E_x(z) + \left[\frac{1}{\eta_0}\left(\frac{\varepsilon_{xy}}{\varepsilon_0} + \frac{k_xk_y}{k_0^2} - \frac{\varepsilon_{xz}\varepsilon_{zy}}{\varepsilon_{zz}\varepsilon_0}\right)\right] ik_0E_y(z) + \left(\frac{k_y}{k_0}\frac{\varepsilon_{xz}}{\varepsilon_{zz}}\right) ik_0H_x(z) + \left(-\frac{k_x}{k_0}\frac{\varepsilon_{xz}}{\varepsilon_{zz}}\right) ik_0H_y(z) \tag{A.49}$$

where η_0 is the impedance of free space and k_0 the wavevector of the fields:

$$\eta_0 = \sqrt{\frac{\mu_0}{\varepsilon_0}} = \mu_0 c \qquad k_0 = \frac{\omega}{c} \qquad c = \frac{1}{\sqrt{\varepsilon_0\mu_0}} \tag{A.50}$$

By substituting $\frac{k}{k_0} = \kappa$ and $\frac{\varepsilon}{\varepsilon_0} \to \varepsilon$ we can write the equations in a matrix form with ε_{ij} denoting the elements of the dielectric tensor of equations (A.27-35):

$$\frac{\partial}{\partial z}\begin{bmatrix}E_x(z)\\E_y(z)\\H_x(z)\\H_y(z)\end{bmatrix} = ik_0\begin{bmatrix} -\kappa_x\frac{\varepsilon_{zx}}{\varepsilon_{zz}} & -\kappa_x\frac{\varepsilon_{zy}}{\varepsilon_{zz}} & \frac{\kappa_x\kappa_y\eta_0}{\varepsilon_{zz}} & \frac{(\varepsilon_{zz}-\kappa_x^2)\eta_0}{\varepsilon_{zz}} \\ -\kappa_y\frac{\varepsilon_{zx}}{\varepsilon_{zz}} & -\kappa_y\frac{\varepsilon_{zy}}{\varepsilon_{zz}} & \frac{\left(\kappa_y^2-\varepsilon_{zz}\right)\eta_0}{\varepsilon_{zz}} & -\frac{\kappa_x\kappa_y\eta_0}{\varepsilon_{zz}} \\ \frac{1}{\eta_0}\left(-\varepsilon_{yx}-\kappa_x\kappa_y+\frac{\varepsilon_{yz}\varepsilon_{zx}}{\varepsilon_{zz}}\right) & \frac{1}{\eta_0}\left(\kappa_x^2-\varepsilon_{yy}+\frac{\varepsilon_{yz}\varepsilon_{zy}}{\varepsilon_{zz}}\right) & -\kappa_y\frac{\varepsilon_{yz}}{\varepsilon_{zz}} & \kappa_x\frac{\varepsilon_{yz}}{\varepsilon_{zz}} \\ \frac{1}{\eta_0}\left(-\kappa_y^2+\varepsilon_{xx}-\frac{\varepsilon_{xz}\varepsilon_{zx}}{\varepsilon_{zz}}\right) & \frac{1}{\eta_0}\left(\varepsilon_{xy}+\kappa_x\kappa_y-\frac{\varepsilon_{xz}\varepsilon_{zy}}{\varepsilon_{zz}}\right) & \kappa_y\frac{\varepsilon_{xz}}{\varepsilon_{zz}} & -\kappa_x\frac{\varepsilon_{xz}}{\varepsilon_{zz}} \end{bmatrix}\begin{bmatrix}E_x(z)\\E_y(z)\\H_x(z)\\H_y(z)\end{bmatrix}$$

(A.51)

In a matrix form equation (A.51) can be expressed as:

$$\frac{\partial}{\partial z}\boldsymbol{\Psi} = ik_0\bar{\Delta}\boldsymbol{\Psi} \tag{A.52}$$

with:

$$\boldsymbol{\Psi} = \begin{bmatrix}E_x(z)\\E_y(z)\\H_x(z)\\H_y(z)\end{bmatrix} \tag{A.53}$$

Equation (A.52) can again be written as a system of four first order differential equations. Taking the differential of the first two equations results in a system of six equations with the fields expressed in terms of the elements of matrix $\bar{\Delta}$:

$$\frac{\partial E_x(z)}{\partial z} = ik_0\left(\Delta_{11}E_x + \Delta_{12}E_y + \Delta_{13}H_x + \Delta_{14}H_y\right) \tag{A.54}$$

$$\frac{\partial E_y(z)}{\partial z} = ik_0\left(\Delta_{21}E_x + \Delta_{22}E_y + \Delta_{23}H_x + \Delta_{24}H_y\right) \tag{A.55}$$

$$\frac{\partial H_x(z)}{\partial z} = ik_0\left(\Delta_{31}E_x + \Delta_{32}E_y + \Delta_{33}H_x + \Delta_{34}H_y\right) \tag{A.56}$$

$$\frac{\partial H_y(z)}{\partial z} = ik_0\left(\Delta_{41}E_x + \Delta_{42}E_y + \Delta_{43}H_x + \Delta_{44}H_y\right) \tag{A.57}$$

$$\frac{\partial^2 E_x(z)}{\partial z^2} = ik_0\left(\Delta_{11}\frac{\partial E_x(z)}{\partial z} + \Delta_{12}\frac{\partial E_y(z)}{\partial z} + \Delta_{13}\frac{\partial H_x(z)}{\partial z} + \Delta_{14}\frac{\partial H_y(z)}{\partial z}\right) \tag{A.58}$$

$$\frac{\partial^2 E_y(z)}{\partial z^2} = ik_0\left(\Delta_{21}\frac{\partial E_x(z)}{\partial z} + \Delta_{22}\frac{\partial E_y(z)}{\partial z} + \Delta_{23}\frac{\partial H_x(z)}{\partial z} + \Delta_{24}\frac{\partial H_y(z)}{\partial z}\right) \tag{A.59}$$

Equations (A.54-59) can then be used to eliminate the magnetic field from the expressions. This leads to a second order differential equation for the electric field:

$$-\frac{\partial^2}{\partial z^2}\boldsymbol{E} + \frac{ik_0}{\varepsilon_{zz}}\bar{A}\frac{\partial}{\partial z}\boldsymbol{E} - \frac{k_0^2}{\varepsilon_{zz}}\bar{B}\boldsymbol{E} = 0 \tag{A.60}$$

where:

$$\boldsymbol{E} = \begin{bmatrix} E_x(z) \\ E_y(z) \end{bmatrix} \tag{A.61}$$

Matrices $\bar{A}$ and $\bar{B}$ have the form:

$$\bar{A} = \begin{bmatrix} A_{11} & A_{12} \\ A_{21} & A_{22} \end{bmatrix} \qquad \bar{B} = \begin{bmatrix} B_{11} & B_{12} \\ B_{21} & B_{22} \end{bmatrix} \tag{A.62-63}$$

with their elements, in terms of κ_i and ε_{ij}, described below, neglecting terms with power of κ higher than two. For matrix $\bar{A}$:

$$\begin{aligned} A_{11} &= -\frac{\kappa_x\left[\kappa_x\kappa_y\varepsilon_{yz} + \kappa_y^2\varepsilon_{zx} + \kappa_x^2(\varepsilon_{xz} + \varepsilon_{zx}) - (\varepsilon_{xz}\varepsilon_{zx})\varepsilon_{zz}\right]}{\kappa_x^2 + \kappa_y^2 - \varepsilon_{zz}} \\ &= -\kappa_x(\varepsilon_{zx} + \varepsilon_{xz}) + \frac{\kappa_x\kappa_y\left(\kappa_y\varepsilon_{xz} - \kappa_x\varepsilon_{yz}\right)}{\kappa_x^2 + \kappa_y^2 - \varepsilon_{zz}} \approx -\kappa_x(\varepsilon_{zx} + \varepsilon_{xz}) \end{aligned} \tag{A.64}$$

$$\begin{aligned} A_{12} &= -\frac{\kappa_x^2\kappa_y\varepsilon_{xz} + \kappa_x^3\varepsilon_{zy} - \kappa_y\varepsilon_{xz}\varepsilon_{zz} + \kappa_x\left[\kappa_y^2\left(\varepsilon_{yz} + \varepsilon_{zy}\right) - \varepsilon_{zy}\varepsilon_{zz}\right]}{\kappa_x^2 + \kappa_y^2 - \varepsilon_{zz}} \\ &= -\left(\kappa_y\varepsilon_{xz} + \kappa_x\varepsilon_{zy}\right) - \frac{\kappa_x\kappa_y^2\varepsilon_{yz}}{\kappa_x^2 + \kappa_y^2 - \varepsilon_{zz}} + \frac{\kappa_y^3\varepsilon_{xz}}{\kappa_x^2 + \kappa_y^2 - \varepsilon_{zz}} \\ &\approx -\left(\kappa_y\varepsilon_{xz} + \kappa_x\varepsilon_{zy}\right) \end{aligned} \tag{A.65}$$

$$A_{21} = \frac{-\kappa_x^2\kappa_y(\varepsilon_{xz} + \varepsilon_{zx}) + \kappa_x\varepsilon_{yz}\left(-\kappa_y^2 + \varepsilon_{zz}\right) + \kappa_y\varepsilon_{zx}\left(-\kappa_y^2 + \varepsilon_{zz}\right)}{\kappa_x^2 + \kappa_y^2 - \varepsilon_{zz}}$$
$$= -\left(\kappa_x\varepsilon_{yz} + \kappa_y\varepsilon_{zx}\right) - \frac{\kappa_x^2\kappa_y\varepsilon_{xz}}{\kappa_x^2 + \kappa_y^2 - \varepsilon_{zz}} + \frac{\kappa_x^3\varepsilon_{yz}}{\kappa_x^2 + \kappa_y^2 - \varepsilon_{zz}}$$
$$\approx -\left(\kappa_x\varepsilon_{yz} + \kappa_y\varepsilon_{zx}\right)$$

(A.66)

$$A_{22} = -\frac{\kappa_y\left[\kappa_x\kappa_y\varepsilon_{xz} + \kappa_x^2\varepsilon_{zy} + \left(\varepsilon_{yz} + \varepsilon_{zy}\right)\left(\kappa_y^2 - \varepsilon_{zz}\right)\right]}{\kappa_x^2 + \kappa_y^2 - \varepsilon_{zz}}$$
$$= -\kappa_y\left(\varepsilon_{yz} + \varepsilon_{zy}\right) + \frac{\kappa_x\kappa_y\left(\kappa_x\varepsilon_{yz} - \kappa_y\varepsilon_{xz}\right)}{\kappa_x^2 + \kappa_y^2 - \varepsilon_{zz}} \approx -\kappa_y\left(\varepsilon_{yz} + \varepsilon_{zy}\right)$$

(A.67)

Similarly, for $\bar{B}$:

$$B_{11} = -\frac{\kappa_x^4\varepsilon_{xx} + \kappa_x^3 k_y\varepsilon_{yx} + \kappa_x\kappa_y\left(\kappa_y^2\varepsilon_{yx} + \varepsilon_{yz}\varepsilon_{zx} - \varepsilon_{yx}\varepsilon_{zz}\right)}{\kappa_x^2 + \kappa_y^2 - \varepsilon_{zz}} - \frac{\varepsilon_{zz}\left[\kappa_y^4 - \varepsilon_{xz}\varepsilon_{zx} + \varepsilon_{xx}\varepsilon_{zz} - \kappa_y^2(\varepsilon_{xx} + \varepsilon_{zz})\right] + \kappa_x^2\left[\varepsilon_{xz}\varepsilon_{zx} - 2\varepsilon_{xx}\varepsilon_{zz} + \kappa_y^2(\varepsilon_{xx} + \varepsilon_{zz})\right]}{\kappa_x^2 + \kappa_y^2 - \varepsilon_{zz}} = -\left[\varepsilon_{xx}\kappa_x^2 + \left(\varepsilon_{zz} + \frac{\varepsilon_{xz}\varepsilon_{zx}}{\varepsilon_{zz}}\right)\kappa_y^2 + \left(\varepsilon_{yx} - \frac{\varepsilon_{yz}\varepsilon_{zx}}{\varepsilon_{zz}}\right)\kappa_x\kappa_y\right] + \varepsilon_{xx}\varepsilon_{zz} - \varepsilon_{xz}\varepsilon_{zx} + \frac{(\kappa_x^2 + \kappa_y^2)\left(\kappa_y^2\frac{\varepsilon_{xz}\varepsilon_{zx}}{\varepsilon_{zz}} - \kappa_x\kappa_y\frac{\varepsilon_{yz}\varepsilon_{zy}}{\varepsilon_{zz}}\right)}{\kappa_x^2 + \kappa_y^2 - \varepsilon_{zz}} \approx -\left[\varepsilon_{xx}\kappa_x^2 + \left(\varepsilon_{zz} + \frac{\varepsilon_{xz}\varepsilon_{zx}}{\varepsilon_{zz}}\right)\kappa_y^2 + \left(\varepsilon_{yx} - \frac{\varepsilon_{yz}\varepsilon_{zx}}{\varepsilon_{zz}}\right)\kappa_x\kappa_y\right] + \varepsilon_{xx}\varepsilon_{zz} - \varepsilon_{xz}\varepsilon_{zx}$$

(A.68)

$$B_{12} = \frac{-\kappa_x^4\varepsilon_{xy} + \kappa_x^3 k_y\left(-\varepsilon_{yy} + \varepsilon_{zz}\right) - \kappa_x^2\left(\kappa_y^2\varepsilon_{xy} + \varepsilon_{xz}\varepsilon_{zy} - 2\varepsilon_{xy}\varepsilon_{zz}\right)}{\kappa_x^2 + \kappa_y^2 - \varepsilon_{zz}}$$
$$+ \frac{\varepsilon_{zz}\left(\kappa_y^2\varepsilon_{xy} + \varepsilon_{xz}\varepsilon_{zy} - \varepsilon_{xy}\varepsilon_{zz}\right) - \kappa_x\kappa_y\left[\varepsilon_{yz}\varepsilon_{zy} + \kappa_y^2\left(\varepsilon_{yy} - \varepsilon_{zz}\right) - \varepsilon_{yy}\varepsilon_{zz} + \varepsilon_{zz}^2\right]}{\kappa_x^2 + \kappa_y^2 - \varepsilon_{zz}} =$$
$$= -\left[\varepsilon_{xy}\kappa_x^2 + \frac{\varepsilon_{xz}\varepsilon_{zy}}{\varepsilon_{zz}}\kappa_y^2 + \left[\varepsilon_{yy} - \left(\varepsilon_{zz} + \frac{\varepsilon_{yz}\varepsilon_{zy}}{\varepsilon_{zz}}\right)\right]\kappa_x\kappa_y\right] + \varepsilon_{xy}\varepsilon_{zz} - \varepsilon_{xz}\varepsilon_{zy}$$
$$+ \frac{\left(\kappa_x^2 + \kappa_y^2\right)\left(\kappa_y^2\frac{\varepsilon_{xz}\varepsilon_{zy}}{\varepsilon_{zz}} - \kappa_x\kappa_y\frac{\varepsilon_{yz}\varepsilon_{zy}}{\varepsilon_{zz}}\right)}{\kappa_x^2 + \kappa_y^2 - \varepsilon_{zz}}$$
$$\approx -\left[\varepsilon_{xy}\kappa_x^2 + \frac{\varepsilon_{xz}\varepsilon_{zy}}{\varepsilon_{zz}}\kappa_y^2 + \left[\varepsilon_{yy} - \left(\varepsilon_{zz} + \frac{\varepsilon_{yz}\varepsilon_{zy}}{\varepsilon_{zz}}\right)\right]\kappa_x\kappa_y\right] + \varepsilon_{xy}\varepsilon_{zz} - \varepsilon_{xz}\varepsilon_{zy}$$

(A.69)

$$
\begin{aligned}
B_{21} &= \frac{\kappa_x^2\varepsilon_{yx}\left(-\kappa_y^2+\varepsilon_{zz}\right)+\kappa_x^3\kappa_y(-\varepsilon_{xx}+\varepsilon_{zz})-\left(\kappa_y^2-\varepsilon_{zz}\right)\left(\kappa_y^2\varepsilon_{yx}+\varepsilon_{yz}\varepsilon_{zx}-\varepsilon_{yx}\varepsilon_{zz}\right)}{\kappa_x^2+\kappa_y^2-\varepsilon_{zz}} \\
&+ \frac{-\kappa_x\kappa_y\left[\varepsilon_{xz}\varepsilon_{zx}+\kappa_y^2(\varepsilon_{xx}-\varepsilon_{zz})-\varepsilon_{xx}\varepsilon_{zz}+\varepsilon_{zz}^2\right]}{\kappa_x^2+\kappa_y^2-\varepsilon_{zz}} \\
&= -\left[\frac{\varepsilon_{yz}\varepsilon_{zx}}{\varepsilon_{zz}}\kappa_x^2+\varepsilon_{yx}\kappa_y^2+\left[\varepsilon_{xx}-\left(\varepsilon_{zz}+\frac{\varepsilon_{xz}\varepsilon_{zx}}{\varepsilon_{zz}}\right)\right]\kappa_x\kappa_y\right]+\varepsilon_{yx}\varepsilon_{zz}-\varepsilon_{yz}\varepsilon_{zx} \\
&+ \frac{\left(\kappa_x^2+\kappa_y^2\right)\left(\kappa_x^2\frac{\varepsilon_{yz}\varepsilon_{zx}}{\varepsilon_{zz}}-\kappa_x\kappa_y\frac{\varepsilon_{xz}\varepsilon_{zx}}{\varepsilon_{zz}}\right)}{\kappa_x^2+\kappa_y^2-\varepsilon_{zz}} \\
&\approx -\left[\frac{\varepsilon_{yz}\varepsilon_{zx}}{\varepsilon_{zz}}\kappa_x^2+\varepsilon_{yx}\kappa_y^2+\left[\varepsilon_{xx}-\left(\varepsilon_{zz}+\frac{\varepsilon_{xz}\varepsilon_{zx}}{\varepsilon_{zz}}\right)\right]\kappa_x\kappa_y\right]+\varepsilon_{yx}\varepsilon_{zz}-\varepsilon_{yz}\varepsilon_{zx}
\end{aligned}
\tag{A.70}
$$

$$
\begin{aligned}
B_{22} = &-\frac{\kappa_x^3\kappa_y\varepsilon_{xy}+\kappa_x^4\varepsilon_{zz}+\kappa_x^2\left(\kappa_y^2-\varepsilon_{zz}\right)\left(\varepsilon_{yy}+\varepsilon_{zz}\right)+\kappa_x\kappa_y\left(\kappa_y^2\varepsilon_{xy}+\varepsilon_{xz}\varepsilon_{zy}-\varepsilon_{xy}\varepsilon_{zz}\right)}{\kappa_x^2+\kappa_y^2-\varepsilon_{zz}} \\
&- \frac{\left(\kappa_y^2-\varepsilon_{zz}\right)\left(\kappa_y^2\varepsilon_{yy}+\varepsilon_{yz}\varepsilon_{zy}-\varepsilon_{yy}\varepsilon_{zz}\right)}{\kappa_x^2+\kappa_y^2-\varepsilon_{zz}} \\
&= -\left[\left(\varepsilon_{zz}+\frac{\varepsilon_{yz}\varepsilon_{zy}}{\varepsilon_{zz}}\right)\kappa_x^2+\varepsilon_{yy}\kappa_y^2+\left(\varepsilon_{xy}-\frac{\varepsilon_{xz}\varepsilon_{zy}}{\varepsilon_{zz}}\right)\kappa_x\kappa_y\right]+\varepsilon_{yy}\varepsilon_{zz} \\
&- \varepsilon_{yz}\varepsilon_{zy}+\frac{\left(\kappa_x^2+\kappa_y^2\right)\left(\kappa_x^2\frac{\varepsilon_{yz}\varepsilon_{zy}}{\varepsilon_{zz}}-\kappa_x\kappa_y\frac{\varepsilon_{xz}\varepsilon_{zy}}{\varepsilon_{zz}}\right)}{\kappa_x^2+\kappa_y^2-\varepsilon_{zz}} \\
&\approx -\left[\left(\varepsilon_{zz}+\frac{\varepsilon_{yz}\varepsilon_{zy}}{\varepsilon_{zz}}\right)\kappa_x^2+\varepsilon_{yy}\kappa_y^2+\left(\varepsilon_{xy}-\frac{\varepsilon_{xz}\varepsilon_{zy}}{\varepsilon_{zz}}\right)\kappa_x\kappa_y\right]+\varepsilon_{yy}\varepsilon_{zz} \\
&- \varepsilon_{yz}\varepsilon_{zy}
\end{aligned}
\tag{A.71}
$$

The matrices can then be written as:

$$
\bar{A} = \begin{bmatrix} -\kappa_x(\varepsilon_{zx}+\varepsilon_{xz}) & -\left(\kappa_y\varepsilon_{xz}+\kappa_x\varepsilon_{zy}\right) \\ -\left(\kappa_x\varepsilon_{yz}+\kappa_y\varepsilon_{zx}\right) & -\kappa_y\left(\varepsilon_{yz}+\varepsilon_{zy}\right) \end{bmatrix} \tag{A.72}
$$

$$
\begin{aligned}
&\bar{B} \\
&= \begin{bmatrix} -\left[\varepsilon_{xx}\kappa_x^2+\left(\varepsilon_{zz}+\frac{\varepsilon_{xz}\varepsilon_{zx}}{\varepsilon_{zz}}\right)\kappa_y^2+\left(\varepsilon_{yx}-\frac{\varepsilon_{yz}\varepsilon_{zx}}{\varepsilon_{zz}}\right)\kappa_x\kappa_y\right]+\varepsilon_{xx}\varepsilon_{zz}-\varepsilon_{xz}\varepsilon_{zx} & -\left[\varepsilon_{xy}\kappa_x^2+\frac{\varepsilon_{xz}\varepsilon_{zy}}{\varepsilon_{zz}}\kappa_y^2+\left[\varepsilon_{yy}-\left(\varepsilon_{zz}+\frac{\varepsilon_{yz}\varepsilon_{zy}}{\varepsilon_{zz}}\right)\right]\kappa_x\kappa_y\right]+\varepsilon_{xy}\varepsilon_{zz}-\varepsilon_{xz}\varepsilon_{zy} \\ -\left[\frac{\varepsilon_{yz}\varepsilon_{zx}}{\varepsilon_{zz}}\kappa_x^2+\varepsilon_{yx}\kappa_y^2+\left[\varepsilon_{xx}-\left(\varepsilon_{zz}+\frac{\varepsilon_{xz}\varepsilon_{zx}}{\varepsilon_{zz}}\right)\right]\kappa_x\kappa_y\right]+\varepsilon_{yx}\varepsilon_{zz}-\varepsilon_{yz}\varepsilon_{zx} & -\left[\left(\varepsilon_{zz}+\frac{\varepsilon_{yz}\varepsilon_{zy}}{\varepsilon_{zz}}\right)\kappa_x^2+\varepsilon_{yy}\kappa_y^2+\left(\varepsilon_{xy}-\frac{\varepsilon_{xz}\varepsilon_{zy}}{\varepsilon_{zz}}\right)\kappa_x\kappa_y\right]+\varepsilon_{yy}\varepsilon_{zz}-\varepsilon_{yz}\varepsilon_{zy} \end{bmatrix}
\end{aligned}
\tag{A.73}
$$

The matrix $\bar{B}$ can be split into a part $\bar{B}_1$with κ dependence and one $\bar{B}_0$ without. Equation (A.60) will then be:

$$-\frac{\partial^2}{\partial z^2}\boldsymbol{E} + \bar{A}\frac{\partial}{\partial z}\boldsymbol{E} + \bar{B}_1\boldsymbol{E} = k_0^2\bar{B}_0\boldsymbol{E} \qquad \text{(A.74)}$$

By inserting k_0 into the elements of $\bar{A}$ and $\bar{B}_1$, the matrices will become:

$$\bar{A} = \frac{-i}{\varepsilon_{zz}}\begin{bmatrix} k_x(\varepsilon_{zx} + \varepsilon_{xz}) & k_y\varepsilon_{xz} + k_x\varepsilon_{zy} \\ k_x\varepsilon_{yz} + k_y\varepsilon_{zx} & k_y(\varepsilon_{yz} + \varepsilon_{zy}) \end{bmatrix} \qquad \text{(A.75)}$$

$$\bar{B}_1 = \frac{1}{\varepsilon_{zz}}\begin{bmatrix} \varepsilon_{xx}k_x^2 + \tilde{\varepsilon}_{zzx}k_y^2 + \tilde{\varepsilon}_{xy}k_xk_y & \varepsilon_{xy}k_x^2 + \frac{\varepsilon_{xz}\varepsilon_{zy}}{\varepsilon_{zz}}k_y^2 + (\varepsilon_{yy} - \tilde{\varepsilon}_{zzy})k_xk_y \\ \frac{\varepsilon_{yz}\varepsilon_{zx}}{\varepsilon_{zz}}k_x^2 + \varepsilon_{yx}k_y^2 + (\varepsilon_{xx} - \tilde{\varepsilon}_{zzx})k_xk_y & \tilde{\varepsilon}_{zzy}k_x^2 + \varepsilon_{yy}k_y^2 + \tilde{\varepsilon}_{xy}k_xk_y \end{bmatrix}$$

(A.76)

$$\bar{B}_0 = \begin{bmatrix} \tilde{\varepsilon}_{xx} & \tilde{\varepsilon}_{xy} \\ \tilde{\varepsilon}_{xy} & \tilde{\varepsilon}_{yy} \end{bmatrix} \qquad \text{(A.77)}$$

where:

$$\tilde{\varepsilon}_{zzx} = \varepsilon_{zz} + \frac{\varepsilon_{xz}\varepsilon_{zx}}{\varepsilon_{zz}} \qquad \text{(A.78)}$$

$$\tilde{\varepsilon}_{zzy} = \varepsilon_{zz} + \frac{\varepsilon_{yz}\varepsilon_{zy}}{\varepsilon_{zz}} \qquad \text{(A.79)}$$

$$\tilde{\varepsilon}_{xy} = \varepsilon_{xy} - \frac{\varepsilon_{xz}\varepsilon_{zy}}{\varepsilon_{zz}} = \varepsilon_{yx} - \frac{\varepsilon_{yz}\varepsilon_{zx}}{\varepsilon_{zz}} \qquad \text{(A.80)}$$

$$\tilde{\varepsilon}_{xx} = \varepsilon_{xx} - \frac{\varepsilon_{xz}\varepsilon_{zx}}{\varepsilon_{zz}} \qquad \text{(A.81)}$$

$$\tilde{\varepsilon}_{yy} = \varepsilon_{yy} - \frac{\varepsilon_{yz}\varepsilon_{zy}}{\varepsilon_{zz}} \qquad \text{(A.82)}$$

The electric field can be expanded into a sum of orthogonal basis functions as:

$$\boldsymbol{E}(z) = \begin{bmatrix} E_x(z) \\ E_y(z) \end{bmatrix} = \sum_{s=1}^{2}\sum_{n=1}^{N} f_{sn}|s,n\rangle$$

(A.83)

The components on the x and y axis of the laboratory coordinate system will then be:

$$\boldsymbol{E}_x(z) = \sum_{m=1}^{M} f_{1m}|1,m\rangle \qquad \boldsymbol{E}_y(z) = \sum_{n=1}^{N} f_{2n}|2,n\rangle \qquad \text{(A.84-85)}$$

with $s = 1$ denoting the polarization along the x axis and $s = 2$ along the y axis. The basis functions for the two polarizations in the cavity can be chosen to be:

$$|1,m\rangle = \sqrt{\frac{2}{L}}\sin\left(\frac{m\pi z}{L}\right)\begin{bmatrix}1\\0\end{bmatrix} \qquad \text{(A.86)}$$

$$|2,n\rangle = \sqrt{\frac{2}{L}}\sin\left(\frac{n\pi z}{L}\right)\begin{bmatrix}0\\1\end{bmatrix} \qquad \text{(A.87)}$$

In this sense, the electric field consists of a sum of plane waves, or a Fourier series, with frequencies multiplied by an integer, m for $s = 1, (m = 1, 2, \dots., M)$ and n for $s = 2, (n = 1, 2, \dots., N)$. The matrix elements of equation (A.74) can then be represented as:

$$-\frac{\partial^2}{\partial z^2}\boldsymbol{E} + \bar{A}\frac{\partial}{\partial z}\boldsymbol{E} + \bar{B}_1\boldsymbol{E} = k_0^2\bar{B}_0\boldsymbol{E} \rightarrow$$

$$\sum_{\substack{s=1\\s'=1}}^{2}\sum_{\substack{n=1\\m=1}}^{N,M} f_{sn}\left(-\langle s',m|\frac{\partial^2}{\partial z^2}|s,n\rangle\right) + \sum_{\substack{s=1\\s'=1}}^{2}\sum_{\substack{n=1\\m=1}}^{N,M} f_{sn}\langle s',m|\bar{A}\frac{\partial}{\partial z}|s,n\rangle$$
$$+\sum_{\substack{s=1\\s'=1}}^{2}\sum_{\substack{n=1\\m=1}}^{N,M} f_{sn}\langle s',m|\bar{B}_1|s,n\rangle = \sum_{\substack{s=1\\s'=1}}^{2}\sum_{\substack{n=1\\m=1}}^{N,M} k_0^2 f_{sn}\langle s',m|\bar{B}_0|s,n\rangle$$

(A.88)

Each of the terms in equation (A.88) can be calculated according to the definitions (A.86) and (A.87) as follows:

For the matrix elements of the second derivative term:

$$\langle s',m|\frac{\partial^2}{\partial z^2}|s,n\rangle = \begin{cases} s' \neq s \rightarrow \langle s'|s\rangle = 0 \rightarrow \langle s',m|\frac{\partial^2}{\partial z^2}|s,n\rangle = 0 \\ s' = s \rightarrow \langle s',m|\frac{\partial^2}{\partial z}|s,n\rangle = \langle m|\frac{\partial^2}{\partial z^2}|n\rangle \end{cases}$$

$$\langle m|\frac{\partial^2}{\partial z^2}|n\rangle = -\frac{2}{L}\frac{n^2\pi^2}{L^2}\int_0^L \sin\left(\frac{m\pi z}{L}\right)\sin\left(\frac{n\pi z}{L}\right)dz \begin{cases} m = n \to -\frac{2n^2\pi^2}{L^3}\int_0^L \sin^2\left(\frac{n\pi z}{L}\right)dz = -\frac{n^2\pi^2}{L^2} \\ m \neq n \to \int_0^L \sin\left(\frac{m\pi z}{L}\right)\sin\left(\frac{n\pi z}{L}\right)dz = 0 \end{cases}$$

And so:

$$\langle s', m|\frac{\partial^2}{\partial z^2}|s, n\rangle = -\frac{n^2\pi^2}{L^2}\delta_{mn}\delta_{s's} \qquad \text{(A.89)}$$

For the matrix elements of the first derivative term*:*

$$\langle s', m|\bar{A}\frac{\partial}{\partial z}|s, n\rangle = \langle s'|\bar{A}|s\rangle\langle m|\frac{\partial}{\partial z}|n\rangle = A_{s's}\frac{2n\pi}{L^2}\int_0^L \sin\left(\frac{m\pi z}{L}\right)\cos\left(\frac{n\pi z}{L}\right)dz$$

$$\int_0^L \sin\left(\frac{m\pi z}{L}\right)\cos\left(\frac{n\pi z}{L}\right)dz = \begin{cases} m = n \to \int_0^L \sin\left(\frac{m\pi z}{L}\right)\cos\left(\frac{n\pi z}{L}\right)dz = 0 \\ m \neq n \text{ and same parity} \to \int_0^L \sin\left(\frac{m\pi z}{L}\right)\cos\left(\frac{n\pi z}{L}\right)dz = 0 \\ m \neq n \text{ and different parity} \to \int_0^L \sin\left(\frac{m\pi z}{L}\right)\cos\left(\frac{n\pi z}{L}\right)dz = \frac{2mL}{(m^2-n^2)\pi} \end{cases}$$

$$\langle s', m|\bar{A}\frac{\partial}{\partial z}|s, n\rangle = A_{s's}\cdot\begin{cases} \frac{4nm}{L(m^2-n^2)} & \text{if } n, m \text{ are of different parity} \\ 0 & \text{if } n, m \text{ are of same parity} \end{cases} \qquad \text{(A.90)}$$

Lastly, for the $\bar{B}_1$ and B_0 matrix elements:

$$\langle s', m|\bar{B}_1|s, n\rangle = \langle s'|\bar{B}_1|s\rangle\langle m||n\rangle = (B_1)_{s's}\frac{2}{L}\int_0^L \sin\left(\frac{m\pi z}{L}\right)\sin\left(\frac{n\pi z}{L}\right)dz = \begin{cases} m = n \to (B_1)_{s's} \\ m \neq n \to 0 \end{cases}$$

$$\langle s', m|\bar{B}_1|s, n\rangle = (B_1)_{s's}\delta_{mn} \qquad \text{(A.91)}$$

and similarly, for B_0:

$$\langle s', m|\bar{B}_0|s, n\rangle = (B_0)_{s's}\delta_{mn} \tag{A.92}$$

For $k_x = k_y = 0$, the elements of matrices $\bar{A}$ and $\bar{B}_1$ are zero $\bar{A} = \bar{B}_1 = 0$. Equation (A.88) then becomes:

$$-\frac{\partial^2}{\partial z^2}\boldsymbol{E} = k_0^2 \bar{B}_0 \boldsymbol{E}$$

$$\sum_{\substack{s=1\\s'=1}}^{2}\sum_{\substack{n=1\\m=1}}^{N,M} f_{sn}\left(-\langle s', m|\frac{\partial^2}{\partial z^2}|s, n\rangle\right) = \sum_{\substack{s=1\\s'=1}}^{2}\sum_{\substack{n=1\\m=1}}^{N,M} k_0^2 f_{sn}\langle s', m|\bar{B}_0|s, n\rangle$$

$$\frac{n^2\pi^2}{L^2}\delta_{mn}\delta_{s's} = k_0^2 (B_0)_{s's}\delta_{mn} \tag{A.93}$$

- For x axis polarization $s' = s = 1$ and $m = n$:

$$\frac{m^2\pi^2}{L^2} = k_{1m}^2 (B_0)_{11}$$

$$\frac{m^2\pi^2}{L^2} = k_{1m}^2 \tilde{\varepsilon}_{xx} = k_{1m}^2 \tilde{n}_{xx}^2 \tag{A.94}$$

$$\omega_{1m} = ck_{1m} = \frac{c\pi m}{L\tilde{n}_{xx}} \tag{A.95}$$

- For y axis polarization $s' = s = 2$ and $m = n$:

$$\frac{n^2\pi^2}{L^2} = k_{2n}^2 (B_0)_{22}$$

$$\frac{n^2\pi^2}{L^2} = k_{2n}^2 \tilde{\varepsilon}_{yy} = k_{2n}^2 \tilde{n}_{yy}^2 \tag{A.96}$$

$$\omega_{2n} = ck_{2n} = \frac{c\pi n}{L\tilde{n}_{yy}} \tag{A.97}$$

  with k_{1m}, ω_{1m} and k_{2n}, ω_{2n} being the wavevectors and frequencies of electric field modes polarized along the x and y axis, respectively.

For small k vectors and well separated polarization modes $\omega_{1m} \neq \omega_{2n}$ there is no interaction between modes and equation (A.88) with $m = n$ becomes:

$$\sum_{\substack{s=1\\s'=1}}^{2}\sum_{\substack{n=1\\m=1}}^{N,M} f_{sn}\left(-\langle s',m|\frac{\partial^2}{\partial z^2}|s,n\rangle\right)+\sum_{\substack{s=1\\s'=1}}^{2}\sum_{\substack{n=1\\m=1}}^{N,M} f_{sn}\langle s',m|\bar{B}_1|s,n\rangle$$
$$=\sum_{\substack{s=1\\s'=1}}^{2}\sum_{\substack{n=1\\m=1}}^{N,M} k_0^2 f_{sn}\langle s',m|\bar{B}_0|s,n\rangle$$

$$\frac{n^2\pi^2}{L^2}\delta_{mn}\delta_{s's}+(B_1)_{s's}\delta_{mn}=k_0^2(B_0)_{s's}\delta_{mn} \tag{A.96}$$

- For x axis polarization $s'=s=1$:

$$\frac{m^2\pi^2}{L^2}+(B_1)_{11}=k_0^2(B_0)_{11}$$

By using equation (A.94) for small wavevectors we have:

$$k_{1m}^2\tilde{\varepsilon}_{xx}+\frac{1}{\varepsilon_{zz}}\left(\varepsilon_{xx}k_x^2+\tilde{\varepsilon}_{zzx}k_y^2+\tilde{\varepsilon}_{xy}k_xk_y\right)=k_0^2\tilde{\varepsilon}_{xx}$$

$$(k_0^2-k_{1m}^2)\tilde{\varepsilon}_{xx}=\frac{1}{\varepsilon_{zz}}\left(\varepsilon_{xx}k_x^2+\tilde{\varepsilon}_{zzx}k_y^2+\tilde{\varepsilon}_{xy}k_xk_y\right) \tag{A.97}$$

- For y axis polarization $s'=s=2$:

$$\frac{n^2\pi^2}{L^2}+(B_1)_{22}=k_0^2(B_0)_{22}$$

By using equation (A.96) for small wavevectors we have:

$$k_{2n}^2\tilde{\varepsilon}_{yy}+\frac{1}{\varepsilon_{zz}}\left(\tilde{\varepsilon}_{zzy}k_x^2+\varepsilon_{yy}k_y^2+\tilde{\varepsilon}_{xy}k_xk_y\right)=k_0^2\tilde{\varepsilon}_{yy}$$

$$(k_0^2-k_{2n}^2)\tilde{\varepsilon}_{yy}=\frac{1}{\varepsilon_{zz}}\left(\tilde{\varepsilon}_{zzy}k_x^2+\varepsilon_{yy}k_y^2+\tilde{\varepsilon}_{xy}k_xk_y\right) \tag{A.98}$$

For $\omega_{1m}\approx\omega_{2n}$ we must also take into consideration the possible interaction between the modes of different polarization. In this case we have:

$$\omega_{1m}\approx\omega_{2n}\rightarrow ck_{1m}\approx ck_{2n}\rightarrow\frac{c\pi m}{L\sqrt{\tilde{\varepsilon}_{xx}}}\approx\frac{c\pi n}{L\sqrt{\tilde{\varepsilon}_{yy}}}\rightarrow\frac{m}{n}=\sqrt{\frac{\tilde{\varepsilon}_{xx}}{\tilde{\varepsilon}_{yy}}} \tag{A.99}$$

For $\omega_{1m} \approx \omega_{2n}$ and $m = n$ for different polarization s, equation (A.88) would be:

$$\sum_{\substack{s=1\\s'=1}}^{2}\sum_{\substack{n=1\\m=1}}^{N,M} f_{sn}\left(-\langle s',m|\frac{\partial^2}{\partial z^2}|s,n\rangle\right) + \sum_{\substack{s=1\\s'=1}}^{2}\sum_{\substack{n=1\\m=1}}^{N,M} f_{sn}\langle s',m|\bar{B}_1|s,n\rangle = \sum_{\substack{s=1\\s'=1}}^{2}\sum_{\substack{n=1\\m=1}}^{N,M} k_0^2 f_{sn}\langle s',m|\bar{B}_0|s,n\rangle$$

$$\sum_{\substack{s=1\\s'=1}}^{2}\sum_{\substack{n=1\\m=1}}^{N,M} f_{sn}\frac{n^2\pi^2}{L^2}\delta_{mn}\delta_{s's} + \sum_{\substack{s=1\\s'=1}}^{2}\sum_{\substack{n=1\\m=1}}^{N,M} f_{sn}(B_1)_{s's}\delta_{mn} = \sum_{\substack{s=1\\s'=1}}^{2}\sum_{\substack{n=1\\m=1}}^{N,M} k_0^2 f_{sn}(B_0)_{s's}\delta_{mn}$$

$$\sum_{m=1}^{M} f_{1m}\frac{m^2\pi^2}{L^2}\delta_{s'1} + \sum_{n=1}^{N} f_{2n}\frac{n^2\pi^2}{L^2}\delta_{s'2} + \sum_{m=1}^{M} f_{1m}(B_1)_{s'1} + \sum_{n=1}^{N} f_{2n}(B_1)_{s'2} = \sum_{m=1}^{M} k_0^2 f_{1m}(B_0)_{s'1} + \sum_{n=1}^{N} k_0^2 f_{2n}(B_0)_{s'2}$$

- For $s' = 1$:

$$f_{1m}\frac{m^2\pi^2}{L^2} + f_{1m}(B_1)_{11} + f_{2n}(B_1)_{12} = k_0^2 f_{1m}(B_0)_{11} + k_0^2 f_{2n}(B_0)_{12} \qquad \text{(A.100)}$$

- For $s' = 2$:

$$f_{2n}\frac{n^2\pi^2}{L^2} + f_{1m}(B_1)_{21} + f_{2n}(B_1)_{22} = k_0^2 f_{1m}(B_0)_{21} + k_0^2 f_{2n}(B_0)_{22} \qquad \text{(A.101)}$$

Equations (A.100) and (A.101) can be rearranged into:

$$f_{1m}(B_1)_{11} + f_{2n}(B_1)_{12} = f_{1m}k_0^2(B_0)_{11} - f_{1m}\frac{m^2\pi^2}{L^2} + f_{2n}k_0^2(B_0)_{12} \qquad \text{(A.102)}$$

$$f_{1m}(B_1)_{21} + f_{2n}(B_1)_{22} = f_{1m}k_0^2(B_0)_{21} + f_{2n}k_0^2(B_0)_{22} - f_{2n}\frac{n^2\pi^2}{L^2} \qquad \text{(A.103)}$$

and represented into matrix form as:

$$\begin{bmatrix}(B_1)_{11} & (B_1)_{12}\\(B_1)_{21} & (B_1)_{22}\end{bmatrix}\begin{bmatrix}f_{1m}\\f_{2n}\end{bmatrix}=\begin{bmatrix}k_0^2(B_0)_{11}-\frac{m^2\pi^2}{L^2} & k_0^2(B_0)_{12}\\k_0^2(B_0)_{21} & k_0^2(B_0)_{22}-\frac{n^2\pi^2}{L^2}\end{bmatrix}\begin{bmatrix}f_{1m}\\f_{2n}\end{bmatrix} \quad (A.104)$$

Analytically equation (A.104) can be written as:

$$\frac{1}{\varepsilon_{zz}}\begin{bmatrix}\varepsilon_{xx}k_x^2+\tilde{\varepsilon}_{zzx}k_y^2+\tilde{\varepsilon}_{xy}k_xk_y & \varepsilon_{xy}k_x^2+\frac{\varepsilon_{xz}\varepsilon_{zy}}{\varepsilon_{zz}}k_y^2+\left(\varepsilon_{yy}-\tilde{\varepsilon}_{zzy}\right)k_xk_y\\\frac{\varepsilon_{yz}\varepsilon_{zx}}{\varepsilon_{zz}}k_x^2+\varepsilon_{yx}k_y^2+(\varepsilon_{xx}-\tilde{\varepsilon}_{zzx})k_xk_y & \tilde{\varepsilon}_{zzy}k_x^2+\varepsilon_{yy}k_y^2+\tilde{\varepsilon}_{xy}k_xk_y\end{bmatrix}\boldsymbol{F}=$$
$$\begin{bmatrix}(k_0^2-k_{1m}^2)\tilde{\varepsilon}_{xx} & k_0^2\tilde{\varepsilon}_{xy}\\k_0^2\tilde{\varepsilon}_{xy} & (k_0^2-k_{2n}^2)\tilde{\varepsilon}_{yy}\end{bmatrix}\boldsymbol{F} \quad (A.105)$$

where $\boldsymbol{F}$ is the vector containing the polarization coefficients of the electric field:

$$\boldsymbol{F}=\begin{bmatrix}f_{1m}\\f_{2n}\end{bmatrix} \quad (A.106)$$

For $\omega_{1m}\approx\omega_{2n}$ and $m\neq n$ for different polarizations s, with m,n having the same parity, the calculations are the same as previously but because of the presence of δ_{mn} in equation (A.88), when $s\neq s'\rightarrow m\neq n$, the terms $(B_1)_{s's}$ and $(B_0)_{s's}$ will be zero.

- Thus, for $s'=1$ equation (A.88) becomes:

$$f_{1m}\frac{m^2\pi^2}{L^2}+f_{1m}(B_1)_{11}=k_0^2f_{1m}(B_0)_{11} \quad (A.107)$$

- And for $s'=2$:

$$f_{2n}\frac{n^2\pi^2}{L^2}+f_{2n}(B_1)_{22}=k_0^2f_{2n}(B_0)_{22} \quad (A.108)$$

The equation for the coefficients of the electric field then will be:

$$\frac{1}{\varepsilon_{zz}}\begin{bmatrix}\varepsilon_{xx}k_x^2+\tilde{\varepsilon}_{zzx}k_y^2+\tilde{\varepsilon}_{xy}k_xk_y & 0\\0 & \tilde{\varepsilon}_{zzy}k_x^2+\varepsilon_{yy}k_y^2+\tilde{\varepsilon}_{xy}k_xk_y\end{bmatrix}\boldsymbol{F}=$$
$$\begin{bmatrix}(k_0^2-k_{1m}^2)\tilde{\varepsilon}_{xx} & 0\\0 & (k_0^2-k_{2n}^2)\tilde{\varepsilon}_{yy}\end{bmatrix}\boldsymbol{F} \quad (A.109)$$

For $\omega_{1m} \approx \omega_{2n}$ and $m \neq n$ for different polarization s, with m, n having different parity the situation is similar but now the $A_{s's}$ term is not zero by default. This term is:

$$\sum_{\substack{s=1\\s'=1}}^{2}\sum_{\substack{n=1\\m=1}}^{N,M} f_{sn}\langle s', m|\bar{A}\frac{\partial}{\partial z}|s, n\rangle = \sum_{\substack{s=1\\s'=1}}^{2}\sum_{\substack{n=1\\m=1}}^{N,M} f_{sn}\, A_{s's}\frac{4nm}{L(m^2-n^2)}$$

(A.110)

with the property:

$$f_{sn}\langle s', m|\bar{A}\frac{\partial}{\partial z}|s, n\rangle = f_{sn}A_{s's}\frac{4nm}{L(m^2-n^2)} = -f_{sn}A_{s's}\frac{4nm}{L(n^2-m^2)} \quad \text{(A.111)}$$

The terms of $A_{s's}$ will be zero when $m \neq n$ with different parity or $s \neq s'$. Equation (A.110) is then:

$$\sum_{\substack{s=1\\s'=1}}^{2}\sum_{\substack{n=1\\m=1}}^{N,M} f_{sn}\langle s', m|\bar{A}\frac{\partial}{\partial z}|s, n\rangle = \sum_{\substack{n=1\\m=1}}^{N,M} f_{1m}A_{s'1}\frac{4nm}{L(n^2-m^2)} + \sum_{\substack{n=1\\m=1}}^{N,M} f_{2n}A_{s'2}\frac{4nm}{L(m^2-n^2)}$$

- Thus, equation (A.88) for $s' = 1$ can be written as:

$$f_{1m}\frac{m^2\pi^2}{L^2} + f_{1m}(B_1)_{11} + f_{2n}A_{12}\frac{4nm}{L(m^2-n^2)} = k_0^2 f_{1m}(B_0)_{11} \quad \text{(A.112)}$$

- And for $s' = 2$:

$$f_{2n}\frac{n^2\pi^2}{L^2} + f_{2n}(B_1)_{22} + f_{1m}A_{21}\frac{4nm}{L(n^2-m^2)} = k_0^2 f_{2n}(B_0)_{22} \quad \text{(A.113)}$$

Equation (A.109), using the property of equation (A.111), will be modified into the form:

$$\frac{1}{\varepsilon_{zz}}\begin{bmatrix} \varepsilon_{xx}k_x^2 + \tilde{\varepsilon}_{zzx}k_y^2 + \tilde{\varepsilon}_{xy}k_xk_y & -i\frac{4nm}{L(m^2-n^2)}\left(k_y\varepsilon_{xz} + k_x\varepsilon_{zy}\right) \\ i\frac{4nm}{L(m^2-n^2)}\left(k_x\varepsilon_{yz} + k_y\varepsilon_{zx}\right) & \tilde{\varepsilon}_{zzy}k_x^2 + \varepsilon_{yy}k_y^2 + \tilde{\varepsilon}_{xy}k_xk_y \end{bmatrix}\boldsymbol{F} =$$
$$\begin{bmatrix} (k_0^2 - k_{1m}^2)\tilde{\varepsilon}_{xx} & 0 \\ 0 & (k_0^2 - k_{2n}^2)\tilde{\varepsilon}_{yy} \end{bmatrix}\boldsymbol{F} \quad \text{(A.114)}$$

By multiplying equation (A.114) with $c^2/2\omega'$ we get:

$$\frac{c^2}{2\omega'\varepsilon_{zz}}\begin{bmatrix}\varepsilon_{xx}k_x^2+\tilde{\varepsilon}_{zzx}k_y^2+\tilde{\varepsilon}_{xy}k_xk_y & -i\frac{4nm}{L(m^2-n^2)}\left(k_y\varepsilon_{xz}+k_x\varepsilon_{zy}\right)\\ i\frac{4nm}{L(m^2-n^2)}\left(k_x\varepsilon_{yz}+k_y\varepsilon_{zx}\right) & \tilde{\varepsilon}_{zzy}k_x^2+\varepsilon_{yy}k_y^2+\tilde{\varepsilon}_{xy}k_xk_y\end{bmatrix}\boldsymbol{F}=$$

$$\frac{c^2}{2\omega'}\begin{bmatrix}(k_0^2-k_{1m}^2)\tilde{\varepsilon}_{xx} & 0\\ 0 & (k_0^2-k_{2n}^2)\tilde{\varepsilon}_{yy}\end{bmatrix}\boldsymbol{F} \qquad \text{(A.115)}$$

with $\omega=\omega'+\Delta\omega$, $\omega'=\sqrt{\frac{\omega_{1m}^2+\omega_{2n}^2}{2}}$ and assuming $\Delta\omega \ll \omega'$. Neglecting terms of $\Delta\omega^2/\omega'$, the matrix elements of equation (A.115) are modified into:

$$\begin{cases}\frac{c^2}{2\omega'}(k_0^2-k_{1m}^2)=\frac{c^2}{2\omega'}\left(\frac{\omega^2}{c^2}-k_{1m}^2\right)=\frac{c^2}{2\omega'}\left[\frac{(\omega'+\Delta\omega)^2}{c^2}-k_{1m}^2\right]\approx\\ \frac{c^2}{2\omega'}(k_0^2-k_{2n}^2)=\frac{c^2}{2\omega'}\left(\frac{\omega^2}{c^2}-k_{2n}^2\right)=\frac{c^2}{2\omega'}\left[\frac{(\omega'+\Delta\omega)^2}{c^2}-k_{2n}^2\right]\approx\end{cases}$$

$$\begin{cases}\frac{c^2}{2\omega'}\left[\frac{(\omega')^2+2\omega'\Delta\omega}{c^2}-k_{1m}^2\right]=\frac{c^2}{2\omega'}\left(\frac{\omega_{1m}^2+\omega_{2n}^2}{2c^2}+\frac{2\omega'\Delta\omega}{c^2}-k_{1m}^2\right)=\\ \frac{c^2}{2\omega'}\left[\frac{(\omega')^2+2\omega'\Delta\omega}{c^2}-k_{2n}^2\right]=\frac{c^2}{2\omega'}\left(\frac{\omega_{1m}^2+\omega_{2n}^2}{2c^2}+\frac{2\omega'\Delta\omega}{c^2}-k_{2n}^2\right)=\end{cases}$$

$$\begin{cases}\frac{c^2}{2\omega'}\left(\frac{k_{1m}^2}{2}+\frac{k_{2n}^2}{2}-k_{1m}^2\right)+\Delta\omega=\frac{c^2}{4\omega'}(k_{2n}^2-k_{1m}^2)+\Delta\omega\\ \frac{c^2}{2\omega'}\left(\frac{k_{1m}^2}{2}+\frac{k_{2n}^2}{2}-k_{2n}^2\right)+\Delta\omega=\frac{c^2}{4\omega'}(k_{1m}^2-k_{2n}^2)+\Delta\omega\end{cases} \qquad \text{(A.116)}$$

Thus, equation (A.115) becomes:

$$\frac{c^2}{2\omega'\varepsilon_{zz}}\begin{bmatrix}\varepsilon_{xx}k_x^2+\tilde{\varepsilon}_{zzx}k_y^2+\tilde{\varepsilon}_{xy}k_xk_y & -i\frac{4nm}{L(m^2-n^2)}\left(k_y\varepsilon_{xz}+k_x\varepsilon_{zy}\right)\\ i\frac{4nm}{L(m^2-n^2)}\left(k_x\varepsilon_{yz}+k_y\varepsilon_{zx}\right) & \tilde{\varepsilon}_{zzy}k_x^2+\varepsilon_{yy}k_y^2+\tilde{\varepsilon}_{xy}k_xk_y\end{bmatrix}\boldsymbol{F}+$$

$$\frac{c^2}{4\omega'}(k_{1m}^2-k_{2n}^2)\begin{bmatrix}\tilde{\varepsilon}_{xx} & 0\\ 0 & -\tilde{\varepsilon}_{yy}\end{bmatrix}\boldsymbol{F}=\Delta\omega\begin{bmatrix}\tilde{\varepsilon}_{xx} & 0\\ 0 & \tilde{\varepsilon}_{yy}\end{bmatrix}\boldsymbol{F} \qquad \text{(A.116)}$$

Using equation (A.94) for small wavevectors we have:

$$\frac{m^2\pi^2}{L^2}=k_{1m}^2\tilde{\varepsilon}_{xx}\rightarrow m=\frac{L}{\pi c}\omega_{1m}\sqrt{\tilde{\varepsilon}_{xx}}$$

$$\frac{n^2\pi^2}{L^2} = k_{2n}^2\tilde{\varepsilon}_{yy} \rightarrow n = \frac{L}{\pi c}\omega_{2n}\sqrt{\tilde{\varepsilon}_{yy}}$$

$$\frac{mn}{m^2 - n^2} = \frac{\omega_{1m}\omega_{2n}\sqrt{\tilde{\varepsilon}_{yy}\tilde{\varepsilon}_{xx}}}{\omega_{1m}^2\tilde{\varepsilon}_{xx} - \omega_{2n}^2\tilde{\varepsilon}_{yy}}$$

(A.117)

By changing the basis functions to $|1,m\rangle' = \frac{1}{\sqrt{\tilde{\varepsilon}_{xx}}}|1,m\rangle$ and $|2,n\rangle' = \frac{1}{\sqrt{\tilde{\varepsilon}_{yy}}}|2,n\rangle$, the matrix elements with the same s, s', $\langle s',m|\frac{\partial^2}{\partial z^2}|s,n\rangle$, $\langle s',m|\bar{B}_1|s,n\rangle$ and $\langle s',m|\bar{B}_0|s,n\rangle$, will acquire an extra coefficient term, $\frac{1}{\sqrt{\tilde{\varepsilon}_{xx}}}\cdot\frac{1}{\sqrt{\tilde{\varepsilon}_{xx}}} = \frac{1}{\tilde{\varepsilon}_{xx}}$ for $s = 1$ and $\frac{1}{\sqrt{\tilde{\varepsilon}_{yy}}}\cdot\frac{1}{\sqrt{\tilde{\varepsilon}_{yy}}} = \frac{1}{\tilde{\varepsilon}_{yy}}$ for $s = 2$. Thus, $f_{1m} = \frac{f_{1m}'}{\tilde{\varepsilon}_{xx}}$ and $f_{2n} = \frac{f_{2n}'}{\tilde{\varepsilon}_{yy}}$ with $\boldsymbol{E}(z) = f_{1m}'|1,m\rangle' + f_{2n}'|2,n\rangle'$. For the matrix elements $\langle s',m|\bar{A}\frac{\partial}{\partial z}|s,n\rangle$, where $s \neq s'$, the extra coefficients will be $\frac{1}{\sqrt{\tilde{\varepsilon}_{xx}}\sqrt{\tilde{\varepsilon}_{yy}}}$. After this transformation and using equation (A.117), equation (A.116) can be written as:

$$\frac{\hbar c^2}{2\omega'}\begin{bmatrix} \frac{\varepsilon_{xx}k_x^2 + \tilde{\varepsilon}_{zzx}k_y^2 + \tilde{\varepsilon}_{xy}k_xk_y}{\varepsilon_{zz}\tilde{\varepsilon}_{xx}} & 0 \\ 0 & \frac{\tilde{\varepsilon}_{zzy}k_x^2 + \varepsilon_{yy}k_y^2 + \tilde{\varepsilon}_{xy}k_xk_y}{\varepsilon_{zz}\tilde{\varepsilon}_{yy}} \end{bmatrix}\boldsymbol{F}'$$
$$+\frac{\hbar c^2}{2\omega'}\begin{bmatrix} 0 & -4i\frac{\omega_{1m}\omega_{2n}}{L\left(\omega_{1m}^2\tilde{\varepsilon}_{xx} - \omega_{2n}^2\tilde{\varepsilon}_{yy}\right)}\frac{\left(k_x\varepsilon_{yz} + k_y\varepsilon_{xz}\right)}{\varepsilon_{zz}} \\ 4i\frac{\omega_{1m}\omega_{2n}}{L\left(\omega_{1m}^2\tilde{\varepsilon}_{xx} - \omega_{2n}^2\tilde{\varepsilon}_{yy}\right)}\frac{\left(k_x\varepsilon_{yz} + k_y\varepsilon_{xz}\right)}{\varepsilon_{zz}} & 0 \end{bmatrix}\boldsymbol{F}'$$
$$+\frac{\hbar}{4\omega'}\left(\omega_{1m}^2 - \omega_{2n}^2\right)\begin{bmatrix} 1 & 0 \\ 0 & -1 \end{bmatrix}\boldsymbol{F}' = \hbar\Delta\omega\begin{bmatrix} 1 & 0 \\ 0 & 1 \end{bmatrix}\boldsymbol{F}'$$

(A.118)

with $\boldsymbol{F}'$ being the new coefficient vector:

$$\boldsymbol{F}' = \begin{bmatrix} f_{1m}' \\ f_{2n}' \end{bmatrix} \quad \text{(A.119)}$$

Equation (A.118) can also be presented as:

$$\frac{\hbar c^2}{2\omega'}\begin{bmatrix} \frac{\varepsilon_{xx}k_x^2 + \tilde{\varepsilon}_{zzx}k_y^2 + \tilde{\varepsilon}_{xy}k_xk_y}{\varepsilon_{zz}\tilde{\varepsilon}_{xx}} & 0 \\ 0 & \frac{\tilde{\varepsilon}_{zzy}k_x^2 + \varepsilon_{yy}k_y^2 + \tilde{\varepsilon}_{xy}k_xk_y}{\varepsilon_{zz}\tilde{\varepsilon}_{yy}} \end{bmatrix}\boldsymbol{F}' + \frac{2\hbar c^2}{\omega' L}\frac{\omega_{1m}\omega_{2n}}{\left(\omega_{1m}^2\tilde{\varepsilon}_{xx} - \omega_{2n}^2\tilde{\varepsilon}_{yy}\right)}\frac{\left(k_x\varepsilon_{yz} + k_y\varepsilon_{xz}\right)}{\varepsilon_{zz}}\hat{\sigma}_y\boldsymbol{F}' + \frac{\hbar}{4\omega'}(\omega_{1m}^2 - \omega_{2n}^2)\hat{\sigma}_z\boldsymbol{F}' = \hbar\Delta\omega I\boldsymbol{F}'$$

(A.120)

with $\hat{\sigma}_y$, $\hat{\sigma}_z$ the Pauli matrices and I the 2×2 identity matrix:

$$\hat{\sigma}_y = \begin{bmatrix} 0 & -i \\ i & 0 \end{bmatrix}, \quad \hat{\sigma}_z = \begin{bmatrix} 1 & 0 \\ 0 & -1 \end{bmatrix}, \quad I = \begin{bmatrix} 1 & 0 \\ 0 & 1 \end{bmatrix}$$

The second term on the left-hand side of equation (A.120) can be changed into:

$$\frac{2\hbar c^2}{\omega' L}\frac{\omega_{1m}\omega_{2n}}{\left(\omega_{1m}^2\tilde{\varepsilon}_{xx} - \omega_{2n}^2\tilde{\varepsilon}_{yy}\right)}\frac{\left(k_x\varepsilon_{yz} + k_y\varepsilon_{xz}\right)}{\varepsilon_{zz}}\hat{\sigma}_y = \frac{2\hbar c^2}{\omega' L}\frac{mn}{m^2 - n^2}\frac{1}{\sqrt{\tilde{\varepsilon}_{xx}}\sqrt{\tilde{\varepsilon}_{yy}}}\frac{\left(k_x\varepsilon_{yz} + k_y\varepsilon_{xz}\right)}{\varepsilon_{zz}}\hat{\sigma}_y = -2a_xk_x\hat{\sigma}_y - 2a_yk_y\hat{\sigma}_y$$

(A.121)

where we have set the coefficients a_x , a_y and the photon mass for the central frequency ω'as:

$$a_x = -\frac{\hbar^2}{m_0'L}\frac{mn}{(m^2-n^2)}\frac{\varepsilon_{yz}}{\varepsilon_{zz}\sqrt{\tilde{\varepsilon}_{xx}\tilde{\varepsilon}_{yy}}} \tag{A.122}$$

$$a_y = -\frac{\hbar^2}{m_0'L}\frac{mn}{(m^2-n^2)}\frac{\varepsilon_{xz}}{\varepsilon_{zz}\sqrt{\tilde{\varepsilon}_{xx}\tilde{\varepsilon}_{yy}}} \tag{A.123}$$

$$m_0' = \frac{\hbar\omega'}{c^2} \tag{A.124}$$

Assuming $\omega_{1m} \approx \omega_{2n}$ we have:

$$\omega_{1m} \approx \omega_{2n} \rightarrow (\omega_{1m} - \omega_{2n})^2 \approx 0 \rightarrow \omega_{1m}^2 + \omega_{2n}^2 \approx 2\omega_{1m}\omega_{2n} \rightarrow \frac{\omega_{1m}^2 + \omega_{2n}^2}{2\omega_{1m}\omega_{2n}} \approx 1$$

(A.125)

Equation (A.125) enables us to simplify the last term on the left-hand side of equation (A.120) as:

$$\frac{\hbar}{4\omega'}(\omega_{1m}^2-\omega_{2n}^2)=\frac{\hbar}{4}\frac{(\omega_{1m}-\omega_{2n})(\omega_{1m}+\omega_{2n})}{\sqrt{\frac{\omega_{1m}^2+\omega_{2n}^2}{2}}}=\frac{\hbar\sqrt{2}}{4}(\omega_{1m}-\omega_{2n})\sqrt{\frac{1}{\frac{\omega_{1m}^2+\omega_{2n}^2}{(\omega_{1m}^2+\omega_{2n}^2)^2}}}$$

$$=\frac{\hbar\sqrt{2}}{4}(\omega_{1m}-\omega_{2n})\sqrt{\frac{1}{\frac{(\omega_{1m}-\omega_{2n})^2}{(\omega_{1m}^2+\omega_{2n}^2)^2}+\frac{2\omega_{1m}\omega_{2n}}{(\omega_{1m}^2+\omega_{2n}^2)^2}}}$$

$$\approx\frac{\hbar\sqrt{2}}{4}(\omega_{1m}-\omega_{2n})\sqrt{\frac{1}{\frac{2\omega_{1m}\omega_{2n}}{(\omega_{1m}^2+\omega_{2n}^2)^2}}}=\frac{\hbar\sqrt{2}}{4}(\omega_{1m}-\omega_{2n})\sqrt{\frac{(\omega_{1m}^2+\omega_{2n}^2)^2}{2\omega_{1m}\omega_{2n}}}$$

$$=\frac{\hbar\sqrt{2}}{4}(\omega_{1m}-\omega_{2n})\sqrt{1+\frac{\omega_{1m}^2+\omega_{2n}^2}{2\omega_{1m}\omega_{2n}}}\approx\frac{\hbar\sqrt{2}}{4}(\omega_{1m}-\omega_{2n})\sqrt{2}$$

$$=\frac{\hbar}{2}(\omega_{1m}-\omega_{2n})=\frac{1}{2}(E_X-E_Y)$$

(A.126)

where E_X and E_Y are the energies of the x and y axis polarization frequencies respectively:

$$E_X=\hbar\omega_{1m} \qquad E_Y=\hbar\omega_{2n} \qquad \text{(A.127-128)}$$

The matrix on the left-hand side of equation (A.120) can be divided into two:

$$\frac{\hbar c^2}{2\omega'}\begin{bmatrix}\frac{\varepsilon_{xx}k_x^2+\tilde{\varepsilon}_{zzx}k_y^2+\tilde{\varepsilon}_{xy}k_xk_y}{\varepsilon_{zz}\tilde{\varepsilon}_{xx}} & 0\\ 0 & \frac{\tilde{\varepsilon}_{zzy}k_x^2+\varepsilon_{yy}k_y^2+\tilde{\varepsilon}_{xy}k_xk_y}{\varepsilon_{zz}\tilde{\varepsilon}_{yy}}\end{bmatrix}$$

$$=\frac{\hbar^2}{2m_0'}\begin{bmatrix}\frac{\varepsilon_{xx}k_x^2+\tilde{\varepsilon}_{zzx}k_y^2}{\varepsilon_{zz}\tilde{\varepsilon}_{xx}} & 0\\ 0 & \frac{\tilde{\varepsilon}_{zzy}k_x^2+\varepsilon_{yy}k_y^2}{\varepsilon_{zz}\tilde{\varepsilon}_{yy}}\end{bmatrix}+\frac{\hbar^2}{2m_0'}\begin{bmatrix}\frac{\tilde{\varepsilon}_{xy}k_xk_y}{\varepsilon_{zz}\tilde{\varepsilon}_{xx}} & 0\\ 0 & \frac{\tilde{\varepsilon}_{xy}k_xk_y}{\varepsilon_{zz}\tilde{\varepsilon}_{yy}}\end{bmatrix}$$

(A.129)

For the first matrix of equation (A.129), by setting:

$$\frac{\hbar^2}{2m_0'}\begin{bmatrix}\frac{\varepsilon_{xx}k_x^2+\tilde{\varepsilon}_{zzx}k_y^2}{\varepsilon_{zz}\tilde{\varepsilon}_{xx}} & 0\\ 0 & \frac{\tilde{\varepsilon}_{zzy}k_x^2+\varepsilon_{yy}k_y^2}{\varepsilon_{zz}\tilde{\varepsilon}_{yy}}\end{bmatrix}$$

$$=\begin{bmatrix}\frac{\hbar^2k_x^2}{2m_x}+\frac{\hbar^2k_y^2}{2m_y} & 0\\ 0 & \frac{\hbar^2k_x^2}{2m_x}+\frac{\hbar^2k_y^2}{2m_y}\end{bmatrix}+\begin{bmatrix}\delta_xk_x^2+\delta_yk_y^2 & 0\\ 0 & -\delta_xk_x^2-\delta_yk_y^2\end{bmatrix}$$

$$=\begin{bmatrix}\left(\frac{\hbar^2}{2m_x}+\delta_x\right)k_x^2+\left(\frac{\hbar^2}{2m_y}+\delta_y\right)k_y^2 & 0\\ 0 & \left(\frac{\hbar^2}{2m_x}-\delta_x\right)k_x^2+\left(\frac{\hbar^2}{2m_y}-\delta_y\right)k_y^2\end{bmatrix}$$

results into four equations for the coefficients m_x, m_y, δ_x and δ_y:

$$\frac{\hbar^2}{2m_x}+\delta_x=\frac{\hbar^2}{2m_0'}\frac{\varepsilon_{xx}}{\varepsilon_{zz}\tilde{\varepsilon}_{xx}} \tag{A.130}$$

$$\frac{\hbar^2}{2m_y}+\delta_y=\frac{\hbar^2}{2m_0'}\frac{\tilde{\varepsilon}_{zzx}}{\varepsilon_{zz}\tilde{\varepsilon}_{xx}} \tag{A.131}$$

$$\frac{\hbar^2}{2m_x}-\delta_x=\frac{\hbar^2}{2m_0'}\frac{\tilde{\varepsilon}_{zzy}}{\varepsilon_{zz}\tilde{\varepsilon}_{yy}} \tag{A.132}$$

$$\frac{\hbar^2k_x^2}{2m_y}-\delta_y=\frac{\hbar^2}{2m_0'}\frac{\varepsilon_{yy}}{\varepsilon_{zz}\tilde{\varepsilon}_{yy}} \tag{A.133}$$

According to these, the first matrix can be written as:

$$\frac{\hbar^2}{2m_0'}\begin{bmatrix}\frac{\varepsilon_{xx}k_x^2+\tilde{\varepsilon}_{zzx}k_y^2}{\varepsilon_{zz}\tilde{\varepsilon}_{xx}} & 0\\ 0 & \frac{\tilde{\varepsilon}_{zzy}k_x^2+\varepsilon_{yy}k_y^2}{\varepsilon_{zz}\tilde{\varepsilon}_{yy}}\end{bmatrix}=\left(\frac{\hbar^2k_x^2}{2m_x}+\frac{\hbar^2k_y^2}{2m_y}\right)I+\left(\delta_xk_x^2+\delta_yk_y^2\right)\hat{\sigma}_z \tag{A.134}$$

and the coefficients are defined as:

$$\frac{1}{m_x}=\frac{1}{2m_0'}\frac{\varepsilon_{xx}\tilde{\varepsilon}_{yy}+\tilde{\varepsilon}_{zzy}\tilde{\varepsilon}_{xx}}{\varepsilon_{zz}\tilde{\varepsilon}_{xx}\tilde{\varepsilon}_{yy}} \tag{A.135}$$

$$\delta_x=\frac{\hbar^2}{4m_0'}\frac{\varepsilon_{xx}\tilde{\varepsilon}_{yy}-\tilde{\varepsilon}_{zzy}\tilde{\varepsilon}_{xx}}{\varepsilon_{zz}\tilde{\varepsilon}_{xx}\tilde{\varepsilon}_{yy}} \tag{A.136}$$

$$\frac{1}{m_y} = \frac{1}{2m_0'}\frac{\tilde{\varepsilon}_{zzx}\tilde{\varepsilon}_{yy}+\varepsilon_{yy}\tilde{\varepsilon}_{xx}}{\varepsilon_{zz}\tilde{\varepsilon}_{xx}\tilde{\varepsilon}_{yy}} \tag{A.137}$$

$$\delta_y = \frac{\hbar^2}{4m_0'}\frac{\tilde{\varepsilon}_{zzx}\tilde{\varepsilon}_{yy}-\varepsilon_{yy}\tilde{\varepsilon}_{xx}}{\varepsilon_{zz}\tilde{\varepsilon}_{xx}\tilde{\varepsilon}_{yy}} \tag{A.138}$$

Similarly, for the second matrix of equation (A.129), we set:

$$\frac{\hbar^2}{2m_0'}\begin{bmatrix}\frac{\tilde{\varepsilon}_{xy}k_xk_y}{\varepsilon_{zz}\tilde{\varepsilon}_{xx}} & 0 \\ 0 & \frac{\tilde{\varepsilon}_{xy}k_xk_y}{\varepsilon_{zz}\tilde{\varepsilon}_{yy}}\end{bmatrix} = \begin{bmatrix}\frac{\hbar^2k_xk_y}{2m_{xy}} & 0 \\ 0 & \frac{\hbar^2k_xk_y}{2m_{xy}}\end{bmatrix} + \begin{bmatrix}\delta_{xy}k_xk_y & 0 \\ 0 & -\delta_{xy}k_xk_y\end{bmatrix}$$
$$= \begin{bmatrix}\left(\frac{\hbar^2}{2m_{xy}} + \delta_{xy}\right)k_xk_y & 0 \\ 0 & \left(\frac{\hbar^2}{2m_{xy}} - \delta_{xy}\right)k_xk_y\end{bmatrix}$$

and result into two equations for the constants m_{xy} and δ_{xy}:

$$\frac{\hbar^2}{2m_{xy}} + \delta_{xy} = \frac{\hbar^2}{2m_0'}\frac{\tilde{\varepsilon}_{xy}}{\varepsilon_{zz}\tilde{\varepsilon}_{xx}} \tag{A.139}$$

$$\frac{\hbar^2}{2m_{xy}} - \delta_{xy} = \frac{\hbar^2}{2m_0'}\frac{\tilde{\varepsilon}_{xy}}{\varepsilon_{zz}\tilde{\varepsilon}_{yy}} \tag{A.140}$$

Using these, the second matrix of equation (A.129) can be presented as:

$$\frac{\hbar^2}{2m_0'}\begin{bmatrix}\frac{\tilde{\varepsilon}_{xy}k_xk_y}{\varepsilon_{zz}\tilde{\varepsilon}_{xx}} & 0 \\ 0 & \frac{\tilde{\varepsilon}_{xy}k_xk_y}{\varepsilon_{zz}\tilde{\varepsilon}_{yy}}\end{bmatrix} = \frac{\hbar^2k_xk_y}{2m_{xy}}I + \delta_{xy}k_xk_y\hat{\sigma}_z \tag{A.141}$$

with the coefficients defined as:

$$\frac{1}{m_{xy}} = \frac{1}{2m_0'}\frac{\tilde{\varepsilon}_{xy}\left(\tilde{\varepsilon}_{xx}+\tilde{\varepsilon}_{yy}\right)}{\varepsilon_{zz}\tilde{\varepsilon}_{xx}\tilde{\varepsilon}_{yy}} \tag{A.142}$$

$$\delta_{xy} = \frac{\hbar^2}{4m_0'}\frac{\tilde{\varepsilon}_{xy}(\tilde{\varepsilon}_{yy}-\tilde{\varepsilon}_{xx})}{\varepsilon_{zz}\tilde{\varepsilon}_{xx}\tilde{\varepsilon}_{yy}} \tag{A.143}$$

Finally, after all the modifications, equation (A.120) can be written as:

$$\left(\frac{\hbar^2 k_x^2}{2m_x}+\frac{\hbar^2 k_y^2}{2m_y}\right)\boldsymbol{I}\boldsymbol{F}' + \frac{\hbar^2 k_x k_y}{2m_{xy}}\boldsymbol{I}\boldsymbol{F}' + \left(\delta_x k_x^2 + \delta_y k_y^2\right)\hat{\sigma}_z\boldsymbol{F}' + \delta_{xy}k_x k_y\hat{\sigma}_z\boldsymbol{F}' - 2a_x k_x\hat{\sigma}_y\boldsymbol{F}' - 2a_y k_y\hat{\sigma}_y\boldsymbol{F}' + \frac{1}{2}(E_X - E_Y)\hat{\sigma}_z\boldsymbol{F}' = EI\boldsymbol{F}' \tag{A.144}$$

with $E = \hbar\Delta\omega$

From the form of equation (A.144), the effective Hamiltonian of the system in the linear polarization basis can be written as:

$$H = \left(\frac{\hbar^2 k_x^2}{2m_x}+\frac{\hbar^2 k_y^2}{2m_y}+\frac{\hbar^2 k_x k_y}{2m_{xy}}\right)\hat{I} + \left(\delta_x k_x^2 + \delta_y k_y^2 + \delta_{xy}k_x k_y\right)\hat{\sigma}_z - 2\left(a_x k_x + a_y k_y\right)\hat{\sigma}_y + \frac{1}{2}(E_X - E_Y)\hat{\sigma}_z \tag{A.145}$$

By making the basis transformation using $F_\pm = f_1 \mp i f_2$, where the two eigen-bases become the time-reversal conjugates of each other, the effective Hamiltonian can be written in circularly polarized basis as:

$$H = \left(\frac{\hbar^2 k_x^2}{2m_x}+\frac{\hbar^2 k_y^2}{2m_y}+\frac{\hbar^2 k_x k_y}{2m_{xy}}\right)\hat{I} + \left(\delta_x k_x^2 + \delta_y k_y^2 + \delta_{xy}k_x k_y\right)\hat{\sigma}_x - 2\left(a_x k_x + a_y k_y\right)\hat{\sigma}_z + \frac{1}{2}(E_X - E_Y)\hat{\sigma}_x \tag{A.146}$$

S.10 Transfer matrix method for a birefringent cavity

The theoretical framework for studying a birefringent perovskite material using an extended 4×4 transfer matrix method follows Berreman [94], Yeh [95] and Landry [96]. For an electromagnetic wave, with electric and magnetic field amplitudes E_i and H_i, incident on a multilayered structure at an angle u_i with respect to the axis perpendicular to the surface, such as that presented in **Figure S.37** , there would be a reflected (at an angle $u_r = u_i$) and transmitted beam (at an angle u_t) with electric field magnitudes E_r and E_t respectively.

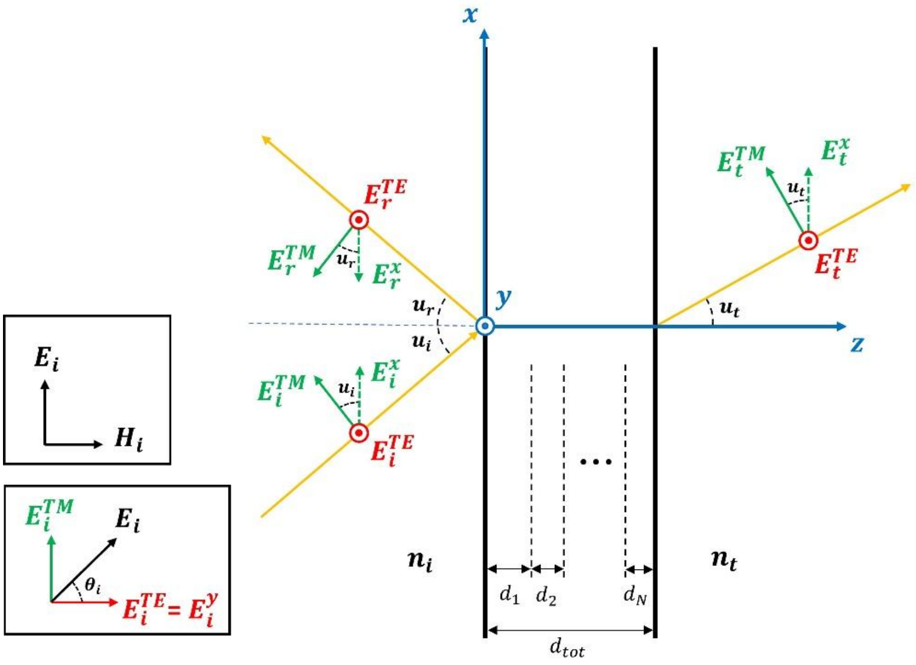

Figure S.37: Sketch of the incident, reflected and transmitted beams of an electromagnetic wave incident on a layered structure. The corresponding angles with respect to the axis normal to the surface are u_i, u_r and u_t, respectively. The lab coordinates are set such that the z axis is perpendicular to the surface of the structure, while x and y axes are parallel to it. The structure is composed of N layers, each having d_N width, with a total width d_{tot}. The electric E and magnetic field H magnitude of the wave can be decomposed into the x, y components and the TE-TM polarizations on the surface of the structure. Lastly, n_i and n_t are the refractive indices of the media before and after the structure, respectively.

The surface of the structure is assumed to lie on the xy plane of the laboratory coordinates, while the z axis is perpendicular to it. The structure is composed of individual N layers with widths d_N and has a total width of d_{tot}. The electric fields E_i , E_r and E_t can be analyzed into TE (s-polarization) and TM (p-polarization) components on the surface of the structure, with θ_i being the angle between the vectors $\boldsymbol{E}_i$ and $\boldsymbol{E}_i^{TE}$. Similar decompositions can be done for the magnetic field H_i, H_r and H_t. The relation of the field components on the x and y axes can be calculated as:

$$\boldsymbol{E}_i^x = \boldsymbol{E}_i^{TM} \cos u_i \qquad \boldsymbol{E}_r^x = -\boldsymbol{E}_r^{TM} \cos u_i \qquad \boldsymbol{E}_t^x = \boldsymbol{E}_t^{TM} \cos u_t$$

$$\boldsymbol{E}_i^y = \boldsymbol{E}_i^{TE} \qquad \boldsymbol{E}_r^y = \boldsymbol{E}_r^{TE} \qquad \boldsymbol{E}_t^y = \boldsymbol{E}_t^{TE}$$

$$\boldsymbol{H}_i^x = \boldsymbol{H}_i^{TM} \cos u_i \;//\; -\boldsymbol{E}_i^{TM} \cos u_i \qquad \boldsymbol{H}_r^x = \boldsymbol{H}_r^{TM} \cos u_i \;//\; -\boldsymbol{E}_r^{TM} \cos u_i$$

$$\boldsymbol{H}_t^x = \boldsymbol{H}_t^{TM} \cos u_t \;//\; -\boldsymbol{E}_t^{TM} \cos u_t$$

$$\boldsymbol{H}_i^y = \boldsymbol{H}_i^{TE} \;//\; \boldsymbol{E}_i^{TE} \qquad \boldsymbol{H}_r^y = \boldsymbol{H}_r^{TE} \;//\; \boldsymbol{E}_r^{TE} \qquad \boldsymbol{H}_t^y = \boldsymbol{H}_t^{TE} \;//\; \boldsymbol{E}_t^{TE}$$

$$\text{with} \quad n|\boldsymbol{E}| = \eta_0 |\boldsymbol{B}|$$

(A.145-157)

If the layers d_N are composed of birefringent materials, the propagation of light through each one of them in z direction can be described by equation A.52 as:

$$\frac{\partial}{\partial z}\boldsymbol{\Psi}_m(z) = ik_0\bar{\Delta}\boldsymbol{\Psi}_m(z) \qquad \text{(A.158)}$$

$$\text{with } \Psi_m(z) = \begin{bmatrix} E_x(z) \\ E_y(z) \\ H_x(z) \\ H_y(z) \end{bmatrix} \text{ for every layer m}$$

$\Psi_m(z)$ can be written as:

$$\boldsymbol{\Psi}_m(z) = exp(ik_{zm}z)\boldsymbol{\Psi}_m(0) \qquad \text{(A.159)}$$

and equation (A.158) is then:

$$k_{zm}\boldsymbol{\Psi}_m(0) = k_0\bar{\Delta}\boldsymbol{\Psi}_m(0) \qquad \text{(A.160)}$$

which is an eigenvalue problem of $k_0\bar{\Delta}$, a 4×4 matrix, and so it has 4 eigenvalues k_{zm} and 4 eigenvectors $\boldsymbol{\Psi}_m(0)$. After the solution of the eigenvalue problem for every layer m with width d_m, a transfer matrix $\bar{P}$ can be written that relates the electric and magnetic fields at the entrance and exit of the layer as:

$$\bar{\Psi}\bar{K} = \bar{P}\bar{\Psi} \rightarrow \bar{P} = \bar{\Psi}\bar{K}\bar{\Psi}^{-1} \qquad \text{(A.161)}$$

where $\bar{K}$ is a diagonal matrix with elements $exp(ik_{zm}d_m)$ and $\bar{\Psi}$ is a 4×4 matrix with columns the eigenvectors $\boldsymbol{\Psi}_m$. For all N layers, the electric and magnetic fields at the entrance $\boldsymbol{\Psi}_i$ and the exit $\boldsymbol{\Psi}_t$ of the structure can be related as:

$$\boldsymbol{\Psi}_t = \bar{P}_{tot}\boldsymbol{\Psi}_i = \bar{\Psi}_N\bar{K}_N\bar{\Psi}_N^{-1} \ldots \bar{\Psi}_2\bar{K}_2\bar{\Psi}_2^{-1}\bar{\Psi}_1\bar{K}_1\bar{\Psi}_1^{-1}\boldsymbol{\Psi}_i \qquad \text{(A.162)}$$

According to equations (A.145-157), the fields $\boldsymbol{\Psi}(z)$ can be written in terms of the TE and TM polarization of the electric field as:

$$\boldsymbol{\Psi}_a = \bar{A}_a \boldsymbol{\Phi}_a \qquad \text{(A.163)}$$

with $\boldsymbol{\Phi}_i = \begin{bmatrix} E_i^{TM} \\ E_r^{TM} \\ E_i^{TE} \\ E_r^{TE} \end{bmatrix}$, $\boldsymbol{\Phi}_t = \begin{bmatrix} E_t^{TM} \\ 0 \\ E_t^{TE} \\ 0 \end{bmatrix}$ and $\bar{A}_a = \begin{bmatrix} \cos u_a & -\cos u_a & 0 & 0 \\ 0 & 0 & 1 & 1 \\ 0 & 0 & -\frac{n_a}{\eta_0}\cos u_a & \frac{n_a}{\eta_0}\cos u_a \\ \frac{n_a}{\eta_0} & \frac{n_a}{\eta_0} & 0 & 0 \end{bmatrix}$

(A.164-166)

having $a \in \{i, t\}$ and n_i, n_t the refractive indices for the mediums before and after the structure. In that way, equation (A.162) can be written as:

$$\boldsymbol{\Phi}_t = \bar{A}_t^{-1} \bar{\Psi}_N \bar{K}_N \bar{\Psi}_N^{-1} \ldots \bar{\Psi}_2 \bar{K}_2 \bar{\Psi}_2^{-1} \bar{\Psi}_1 \bar{K}_1 \bar{\Psi}_1^{-1} \bar{A}_i \boldsymbol{\Phi}_i = \bar{T} \boldsymbol{\Phi}_i \qquad \text{(A.167)}$$

The elements of the transfer matrix $\bar{T}$ can be used to calculate the reflection and transmission coefficients of the structure as:

$$r_{TE-TE} = \left|\frac{E_r^{TE}}{E_i^{TE}}\right|_{E_i^{TM}=0} = \frac{T_{43}T_{11} - T_{41}T_{13}}{T_{33}T_{11} - T_{13}T_{31}} \qquad r_{TE-TM} = \left|\frac{E_r^{TM}}{E_i^{TE}}\right|_{E_i^{TM}=0} = \frac{T_{23}T_{11} - T_{21}T_{13}}{T_{33}T_{11} - T_{13}T_{31}}$$

$$r_{TM-TM} = \left|\frac{E_r^{TM}}{E_i^{TM}}\right|_{E_i^{TE}=0} = \frac{T_{33}T_{21} - T_{31}T_{23}}{T_{33}T_{11} - T_{13}T_{31}} \qquad r_{TM-TE} = \left|\frac{E_r^{TE}}{E_i^{TM}}\right|_{E_i^{TE}=0} = \frac{T_{33}T_{41} - T_{31}T_{43}}{T_{33}T_{11} - T_{13}T_{31}}$$

$$t_{TE-TE} = \left|\frac{E_t^{TE}}{E_i^{TE}}\right|_{E_i^{TM}=0} = \frac{T_{11}}{T_{33}T_{11} - T_{13}T_{31}} \qquad t_{TE-TM} = \left|\frac{E_t^{TM}}{E_i^{TE}}\right|_{E_i^{TM}=0} = \frac{-T_{13}}{T_{33}T_{11} - T_{13}T_{31}}$$

$$t_{TM-TE} = \left|\frac{E_t^{TE}}{E_i^{TM}}\right|_{E_i^{TE}=0} = \frac{-T_{31}}{T_{33}T_{11} - T_{13}T_{31}} \qquad t_{TM-TM} = \left|\frac{E_t^{TM}}{E_i^{TM}}\right|_{E_i^{TE}=0} = \frac{T_{33}}{T_{33}T_{11} - T_{13}T_{31}}$$

(A.168-175)

Where the first and second subscripts signify the input and output polarizations, respectively. The total reflection and transmission coefficients of the structure for every polarization are:

$$t_{TE} = t_{TE-TE} \cos\theta_i + t_{TM-TE} \sin\theta_i \qquad \text{(A.176)}$$

$$t_{TM} = t_{TE-TM} \cos\theta_i + t_{TM-TM} \sin\theta_i \tag{A.177}$$

$$r_{TE} = r_{TE-TE} \cos\theta_i + t_{TM-TE} \sin\theta_i \tag{A.178}$$

$$r_{TM} = r_{TE-TM} \cos\theta_i + t_{TM-TM} \sin\theta_i \tag{A.179}$$

Finally, the reflectivity and transmission of the structure is given by:

$$R_{TE} = |r_{TE}^2|,\ R_{TM} = |r_{TM}^2|,\ T_{TE} = |t_{TE}^2|,\ R_{TM} = |t_{TM}^2| \qquad \text{(A.180-183)}$$

In our case of the perovskite microcavity, we assume the birefringence only occurs in the perovskite material, whose dielectric tensor is presented in **section S.2G**, while the DBRs are optically isotropic with dielectric constants $\epsilon_1 = 2.085$ for Ta_2O_5 and $\epsilon_2 = 1.464$ for SiO_2. For clarification, in this section, $\boldsymbol{E}^{TE}$ and $\boldsymbol{E}^{TM}$ refer to s and p polarization while in the main text $\boldsymbol{E}^{x}$ and $\boldsymbol{E}^{y}$ are defined as $\boldsymbol{E}^{TE}$ and $\boldsymbol{E}^{TM}$.

S.11 Supplementary Videos

Supplementary videos accompany the manuscript on the Light: Science & Applications website (http://www.nature.com/lsa)

Supplementary Video 1 description
Effective magnetic field, Berry curvature and diabolical points dependance on the rotation of the birefringent perovskite crystal inside the microcavity.

Supplementary Video 2 description
Angle and circular polarization resolved photoluminescence of multiple cavity modes inside the microcavity. Signal is acquired after placing a λ/4 waveplate and a polarizer at the collection path. The angles correspond to variable λ/4 waveplate rotation.

References

60. Saparov, B. & Mitzi, D. B. Organic-Inorganic Perovskites: Structural Versatility for Functional Materials Design. *Chemical Reviews* **116**, 4558-4596 (2016).

61. Leng, K. et al. Molecularly thin two-dimensional hybrid perovskites with tunable optoelectronic properties due to reversible surface relaxation. *Nature Materials* **17**, 908–914 (2018).

62. Katan, C., Mercier, N. & Even, J. Quantum and Dielectric Confinement Effects in Lower-Dimensional Hybrid Perovskite Semiconductors. *Chemical Reviews* **119**, 3140–3192 (2019).

63. Brivio, F., Butler, K. T., Walsh, A. & van Schilfgaarde, M. Relativistic quasiparticle self-consistent electronic structure of hybrid halide perovskite photovoltaic absorbers. *Physical Review B* **89**, 155204 (2014).

64. Stranks, S. D. & Snaith, H. J. Metal-halide perovskites for photovoltaic and light-emitting devices. *Nature Nanotechnology* **10**, 391-402 (2015).

65. Sutherland, B. R. & Sargent, E. H. Perovskite photonic sources. *Nature Photonics* **10**, 295-302 (2016).

66. Huo, C. X., Cai, B., Yuan, Z., Ma, B. & Zeng, H. B. Two-Dimensional Metal Halide Perovskites: Theory, Synthesis, and Optoelectronics. *Small Methods* **1**, 1600018 (2017).

67. Papavassiliou, G. Three- and low-dimensional inorganic semiconductors. *Progress in Solid State Chemistry* **25**, 125-270 (1997).

68. Mitzi, D. B. Synthesis, Structure, and Properties of Organic-Inorganic Perovskites and Related Materials. *Progress in Inorganic Chemistry* **48**, 1-121 (1999).

69. Ishihara, T. Optical properties of PbI-based perovskite structures. *Journal of Luminescence* **60-61**, 269-274 (1994).

70. Gippius, N. A., Muljarov, E. A., Tikhodeev, S. G., Ishihara, T. & Keldysh, L. V. Dielectrically confined excitons and polaritons in natural superlattices - perovskite lead iodide semiconductors. *Journal de Physique IV* **3**, C5-437-C5-440 (1993).

71. Muljarov, E. A., Tikhodeev, S. G., Gippius, N. A. & Ishihara, T. Excitons in self-organized semiconductor/insulator superlattices: PbI-based perovskite compounds. *Physical Review B* **51**, 14370-14378 (1995).

72. Su, R. et al. Room-Temperature Polariton Lasing in All-Inorganic Perovskite Nanoplatelets. *Nano Letters* **17**, 3982–3988 (2017).

73. Wang, J. et al. Room Temperature Coherently Coupled Exciton–Polaritons in Two-Dimensional Organic–Inorganic Perovskite. *ACS Nano* **12**, 8382–8389 (2018).

74. Su, R. et al. Room temperature long-range coherent exciton polariton condensate flow in lead halide perovskites. *Science Advances* **4**, eaau0244 (2018).

75. Fieramosca, A. et al. Two-dimensional hybrid perovskites sustaining strong polariton interactions at room temperature. *Science Advances* **5**, eaav9967 (2019).

76. Su, R. et al. Observation of exciton polariton condensation in a perovskite lattice at room temperature. *Nature Physics* **16**, 301–306 (2020).

77. Polimeno, L. et al. Observation of Two Thresholds Leading to Polariton Condensation in 2D Hybrid Perovskites. *Advanced Optical Materials* **8**, 2000176 (2020).

78. Wu, J. et al. Nonlinear Parametric Scattering of Exciton Polaritons in Perovskite Microcavities. *Nano Letters* **21**, 3120–3126 (2021).

79. Park, J. E. et al. Polariton Dynamics in Two-Dimensional Ruddlesden–Popper Perovskites Strongly Coupled with Plasmonic Lattices. *ACS Nano* **16**, 3917-3925 (2022).

80. Blancon, J. C. et al. Scaling law for excitons in 2D perovskite quantum wells. *Nature Communications* **9**, 2254 (2018).

81. Kepenekian, M. et al. Concept of Lattice Mismatch and Emergence of Surface States in Two-dimensional Hybrid Perovskite Quantum Wells. *Nano Letters* **18**, 5603-5609 (2018).

82. Kepenekian, M. et al. Rashba and Dresselhaus Effects in Hybrid Organic-Inorganic Perovskites From Basics to Devices. *ACS Nano* **9**, 11557–11567 (2015).

83. Zheng, F., Tan, L. Z., Liu, S. & Rappe, A. M. Rashba Spin−Orbit Coupling Enhanced Carrier Lifetime in $CH_3NH_3PbI_3$. *Nano Letters* **16**, 7316 (2015).

84. Kepenekian, M. & Even, J. Rashba and Dresselhaus Couplings in Halide Perovskites: Accomplishments and Opportunities for Spintronics and Spin–Orbitronics. *The Journal of Physical Chemistry Letters* **8**, 3362–3370 (2017).

85. Zhai, Y. et al. Giant Rashba splitting in 2D organic-inorganic halide perovskites measured by transient spectroscopies. *Science Advances* **3**, e1700704 (2017).

86. Sercel, P. S., Vardeny, Z. V. & Efros, A. L. Circular dichroism in non-chiral metal halide perovskites. *Nanoscale* **12**, 18067-18078 (2020).

87. Saouma, F. O., Stoumpos, C. C., Wong, J., Kanatzidis, M. G. & Jang, J. I. Selective enhancement of optical nonlinearity in two-dimensional organic-inorganic lead iodide perovskites. *Nature Communications* **8**, 742 (2017).

88. Tsai, H. et al. High efficiency two dimensional Ruddlesden-Popper perovskite solar cells. *Nature* **536**, 312–316 (2016).

89. Mitzi, D. B., Synthesis, Crystal Structure, and Optical and Thermal properties of $(C_4H_9NH_3)_2MI_4$ (M=Ge, Sn, Pb). *Chemistry of Materials* **8**, 791–800 (1996).

90. Yaffe, O. et al. Excitons in ultrathin organic-inorganic perovskite crystals. *Physical Review B* **92**, 045414 (2015).

91. Pedesseau, L. et al. Advances and Promises of Layered Halide Hybrid Perovskite Semiconductors. *ACS Nano* **10**, 9776–9786 (2016).

92. Pan, D. et al. Deterministic fabrication of arbitrary vertical heterostructures of two-dimensional Ruddlesden–Popper halide perovskites. *Nature Nanotechnology* **16**, 159–165 (2021).

93. Panzarini, G. et al. Exciton-light coupling in single and coupled semiconductor microcavities: Polariton dispersion and polarization splitting. *Physical Review B* **59**, 5082–5089 (1999).

94. Berreman, D. W. Optics in Stratified and Anisotropic Media: 4×4-Matrix Formulation. *Journal of the Optical Society of America* **62**, 502–510 (1972).

95. Yeh, P. Electromagnetic propagation in birefringent layered media. *Journal of the Optical Society of America* **69**, 742–756 (1979).

96. Landry, G. D. & Maldonado, T. A. Gaussian beam transmission and reflection from a general anisotropic multilayer structure. *Applied Optics* **35**, 5870–5879 (1996).
