# Supplementary figures and images for: Unveiling asymmetric topological photonic states in anisotropic 2D perovskite microcavities
Source: Light Sci Appl. 2025 May 29;14:207. doi: 10.1038/s41377-025-01852-8 (PMC12122894; doi:10.1038/s41377-025-01852-8)

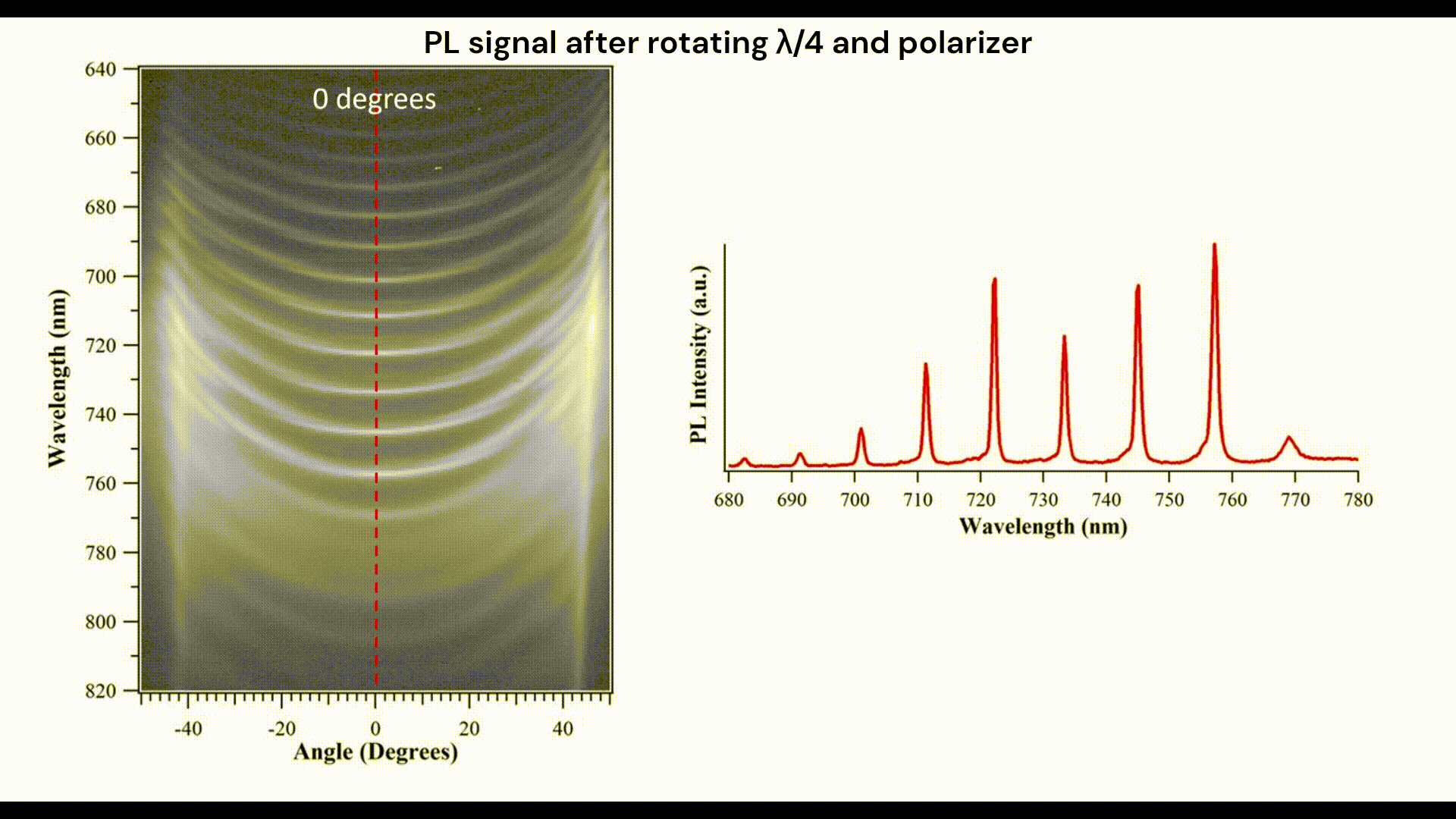

Supplement: Supplementary file 3 — Supplementary Video 2 [file 41377_2025_1852_MOESM3_ESM.gif]
